# Systems serology identifies FcR-related autoantibody signatures and functions for Sjögren’s syndrome

**DOI:** 10.1038/s44321-026-00458-w

**Published:** 2026-06-17

**Authors:** Martin Killian, Suzanne K Shoffner-Beck, Timon Damelang, Kevin John Selva, Kade E Wong, Samantha K Davis, Ebene R Haycroft, Bruce D Wines, P Mark Hogarth, Stephen J Kent, Lucile Grange, Baptiste Gramont, Flora Schein, Héloïse Munoz-Pons, Isabelle Guichard, Jean-Baptiste Gaultier, Anne-Emmanuelle Berger, Alice Haccourt, Blandine Chanut, Fabienne Jospin, Alexis Bocquet, Laurence Bouillet, Marc Ruivard, Yvan Jamilloux, Pascal Sève, Isabelle Durieu, Quitterie Reynaud, Jean-Christophe Lega, Amélie Servettaz, Hubert Marotte, Pascal Cathébras, Rémy Harlé, Edouard Ollier, Stéphane Paul, Kelly B Arnold, Amy W Chung

**Affiliations:** 1 Team GIMAP, CIRI, Université de Lyon, UJM, UCBL1, INSERM U1111, CNRS UMR530, Saint-Etienne, France; 2 Department of Internal Medicine, Saint-Etienne University Hospital, Saint-Etienne, France; 3 Department of Microbiology and Immunology, The Peter Doherty Institute for Infection and Immunity, The University of Melbourne, Melbourne, VIC Australia; 4 Clinical Investigation Center, Inserm CIC1408, Saint-Étienne, France; 5 Department of Biomedical Engineering, University of Michigan, Ann Arbor, MI USA; 6 Immune Therapies Group, Burnet Institute, Melbourne, VIC Australia; 7 Department of Immunology and Pathology, Monash University, Melbourne, VIC Australia; 8 Department of Clinical Pathology, University of Melbourne, Melbourne, VIC Australia; 9 Melbourne Sexual Health Centre and Department of Infectious Diseases, Alfred Hospital and Central Clinical School, Monash University, Melbourne, VIC Australia; 10 Laboratory of Immunology, Saint-Etienne University Hospital, Saint-Etienne, France; 11 Department of Internal Medicine, Grenoble University Hospital, Grenoble, France; 12 Univ. Grenoble Alpes, T-RAIG unit, CNRS, UMR 5525, VetAgro Sup, Grenoble INP, CHU Grenoble Alpes, TIMC, Grenoble, France; 13 Department of Internal Medicine, Hôpital Estaing, Clermont-Ferrand University Hospital, Clermont-Ferrand, France; 14 Department of Internal Medicine, Hôpital de la Croix-Rousse, Hospices Civils de Lyon, Lyon, France; 15 Department of Internal and Vascular Medicine, Hôpital Lyon Sud, Hospices Civils de Lyon, Pierre Bénite, France; 16 RESearch on HealthcAre PErformance (RESHAPE), INSERM U1290, Université Claude Bernard Lyon 1, Lyon, France; 17 University of Lyon, UMR - CNRS 5558, Laboratoire de Biométrie et Biologie Évolutive, Lyon, France; 18 Lyon Immunopathology Federation, Lyon, France; 19 Department of Internal Medicine, Clinical Immunology, Reims University Hospital, and University Reims Champagne Ardenne, IRMAIC, Reims, France; 20 Department of Rheumatology, Saint-Etienne University Hospital, Saint-Etienne, France; 21 Université Jean Monnet Saint-Étienne, Mines Saint-Etienne, INSERM, SAINBIOSE U1059, Saint-Etienne, France; 22 Unité de Recherche Clinique, Saint-Etienne University Hospital, Saint-Etienne, France; 23 Present Address: Univ. Grenoble Alpes, T-RAIG unit, CNRS, UMR 5525, VetAgro Sup, Grenoble INP, CHU Grenoble Alpes, TIMC, Grenoble, France

**Keywords:** Genetics, Gene Therapy & Genetic Disease, Immunology

## Abstract

Sjögren’s Syndrome (SjS) has historically been associated with classical anti-Ro60/SSA, Ro52/SSA and La/SSB, however they are lacking in one third of the patients, which induces delays in diagnosis, and their disease-contributing role is debated. Here we have applied a SjS-tailored Systems Serology approach to a cohort of 58 SjS and 16 non-SjS sicca syndrome patients, and 40 healthy individuals, involving a multiplex assay measuring antibody isotype, subclass, Fc Receptor and complement engagement to 14 SjS-related autoantigens, an antibody-glycosylation profiling assay and a phagocytosis cell-based assay. Via a machine learning approach, we have identified unique autoantibody signatures, including classical and non-classical autoantigens-related features especially involving autoantigen-specific Fc Receptor binding, with apparent functional consequences. These findings provide interesting insights into the autoantibody responses in SjS, possibly paving the way for improved diagnostics, especially in difficult-to-diagnose patients (e.g., seronegative SjS and non-SjS sicca syndrome patients), and novel therapeutic options targeting autoantibody-specific Fc/Fc Receptor-related effector functions.

## Synopsis

This study reports that a Systems Serology approach, dedicated to the evaluation multiple Sjögren’s syndrome autoantibodies’ Fab and Fc related features, could possibly improve its diagnosis, and reveal novel pathogenic mechanisms.

- Autoantibody-specific Fcγ receptor engagement was a major component of all the identified autoantibody signatures in SjS.
- Anti-Ro60 FcγRIIa binding in multiplex strongly correlated with antigen-specific antibody-dependent cellular phagocytosis.
- Altered total IgG glycosylation patterns (low fucosylation and galactosylation) were identified, in line with enhanced Fc receptor binding.
- Autoantibodies targeting the “classical” autoantigens (Ro60, Ro52, La) were the main contributors of the signatures, but multiple “non-classical” autoantibodies (e.g., anti-Chmr3, anti-Cfl1, anti-Rgi2, anti-Ifi16 and/or anti-Ca6) were also reported.

## Abstract (web summary)

This study reports that a Systems Serology approach, dedicated to the evaluation multiple Sjögren’s syndrome autoantibodies’ Fab and Fc related features, could possibly improve its diagnosis, and reveal novel pathogenic mechanisms.

## Graphical abstract

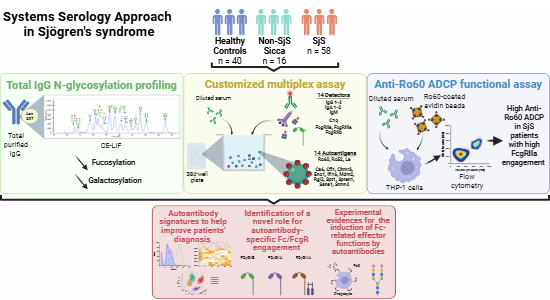

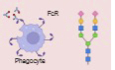

The paper explained **Problem**

Sjögren’s syndrome (SjS) is a complex systemic autoimmune disease characterized by exocrine gland dysfunction and chronic inflammation. Its diagnosis relies heavily on anti-Ro60/SSA, anti-Ro52/SSA and anti-La/SSB autoantibodies, however up to one third of the patients are seronegative, leading to delayed diagnosis and invasive investigations. Moreover, the contribution of autoantibodies to SjS’ pathogenesis remains poorly understood.

**Results**

We applied a SjS-tailored Systems Serology approach combining multiplex autoantibody profiling, Fc receptor engagement and complement activation analyses, IgG glycosylation profiling, and functional phagocytosis assays. Via Machine learning methods, we identified distinct autoantibody signatures that discriminated SjS patients from healthy controls and non-SjS sicca syndrome patients. Besides classical anti-Ro60/SSA, anti-Ro52/SSA and anti-La/SSB IgG responses, the identified signatures included non-classical autoantibodies, and specific Fcγ receptor engagement. Importantly, altered IgG glycosylation patterns associated with enhanced Fc receptor binding were observed, and anti-Ro60 FcγRIIa engagement in multiplex strongly correlated with antibody-dependent cellular phagocytosis, supporting a functional role for Fc-mediated autoantibody activity in SjS

**Impact**

These findings confirmed that autoantibodies are pivotal in SjS, and revealed that autoantibody Fc-related functions could be major features of SjS-associated immune responses. Including evaluations of broader autoantibodies-related features to larger studies, in addition to their specificities, may improve diagnostic stratification, and help identify novel therapeutic targets aimed at modulating pathogenic autoantibody-Fc/Fc receptor interactions.

## Introduction

Sjögren’s Syndrome (SjS) is an autoimmune disorder in which the exocrine glands are infiltrated by immune cells. Its key feature is sicca syndrome, which manifests as dry eyes (keratoconjunctivitis sicca) and dry mouth (xerostomia). Some patients experience articular symptoms or other more serious systemic involvement of other major organs, including the renal, pulmonary, and nervous systems (Mariette and Criswell, 2018; Ramos-Casals et al, 2012, 2014). The disease can be primary or co-exist with another autoimmune disease, such as systemic lupus erythematosus (SLE), rheumatoid arthritis, or scleroderma. The disease pathogenesis is thought to be a multistep process, beginning with an environmental trigger, such as viral infection, in a genetically predisposed person, leading to the production of autoantibodies by autoreactive B cell clones (Sandhya et al, 2017). B cells play a pivotal role in the pathogenesis of SjS, however the precise pathogenic role of the autoantibodies has yet to be confirmed, as the chronic inflammation of the lacrimal and salivary glands and other affected organs is a complex multifactorial process, involving multiple other cell types and immune pathways (Nocturne and Mariette, 2018; Brito-Zerón et al, 2016). Anti-Ro/SSA (present in 60–80% of patients) and anti-La/SSB (40–60% patients) immunoglobulins G (IgG) have been historically identified as important in SjS (Reichlin, 1998; Vílchez-Oya et al, 2022). Anti-Ro/SSA antibodies can be specific to Ro60/TROVE2 (Hung et al, 2015), an RNA binding protein involved in removing misfolded RNA transcripts, and Ro52/TRIM21, an intracellular fragment crystallizable (Fc) Receptor also bearing an E3 ubiquitin ligase activity (Jones et al, 2021; Decker et al, 2022; Trzeciak et al, 2018; Szczerba et al, 2016). Anti-Ro60 and Anti-Ro52 IgG can appear before symptom onset and have a high positive predictive value for development of disease (Fayyaz et al, 2016). Anti-Ro/SSA and La/SSB IgG are also widely used to aid with diagnosis via serologic tests which have been included in all sets of classification criteria to date (Vílchez-Oya et al, 2022; Shiboski et al, 2017; Vitali et al, 2002). While many studies have associated individual autoantibodies with SjS, the role of antibodies in the pathogenesis of disease is still largely unknown.

Antibodies are composed of two Fab (Fragment antigen binding) regions, which can bind to antigenic epitopes and a Fc region, which can bind to Fc receptors (FcRs) on innate immune cells or complement to induce Fc-related effector functions, including antibody-dependent cellular phagocytosis (ADCP), cytotoxicity, release of cytokines leading to inflammatory cascades and/or complement activation. There are multiple antibody isotypes (i.e., IgG, IgA, IgM, IgD, IgE) and subclasses (IgG1-4, IgA1-2), with specific activating (e.g., FcγRIIa for IgG, which is important for ADCP (Richards et al, 2008)) and inhibitory FcRs (e.g. FcγRIIb for IgG, whose expression is down-regulated in active SLE and SjS (Su et al, 2007; Chen et al, 2013)), that balance pro-inflammatory and anti-inflammatory effector responses to provide protection against pathogens, though when dysregulated can lead to disease (Chalayer et al, 2022).

Aberrant expression or allelic variants of FcγRs have been previously associated with autoimmune diseases (Tsang-A-Sjoe et al, 2016), possibly via impaired effector functions (Takai, 2002) or impaired clearance of immune complexes (Koene et al, 1998), but their role in SjS is not yet fully understood (Ben Mkaddem et al, 2019). One study found FcγR-mediated clearance to be abnormal in SjS’ patients (Hamburger et al, 1979), though others have not found SjS-linked FcγR-coding gene variants (Haldorsen et al, 2013). Further evaluation of SjS-associated autoantibodies and related Fc effector activity may lead to the identification of novel specific therapeutic strategies or support the repurposing of currently developed autoantibody-targeting drugs.

Here, we applied systems serology, a fusion of high-throughput experimental antibody assays with computational approaches that was originally applied to identify antibody features and functions associated with control of infectious diseases (Chung et al, 2015; Lu et al, 2016; Arnold and Chung, 2018; Aitken et al, 2021). This approach, however, can also be applied to any antibody-associated disease, including certain autoimmune diseases (Arnold and Chung, 2018; Chung et al, 2020). Thus, here we applied high-throughput assays to assess antibody binding to 14 autoantigens, FcγR engagement, complement activation and glycosylation, in a cohort of SjS, non-SjS sicca syndrome and healthy individuals and applied a machine learning approach that identified interesting autoantigen-specific antibody and FcγR signatures associated with SjS.

## Results

### IgG and FcγR autoantibody signatures distinguish between healthy individuals and those with Sjögren’s syndrome

**Figure 1:**
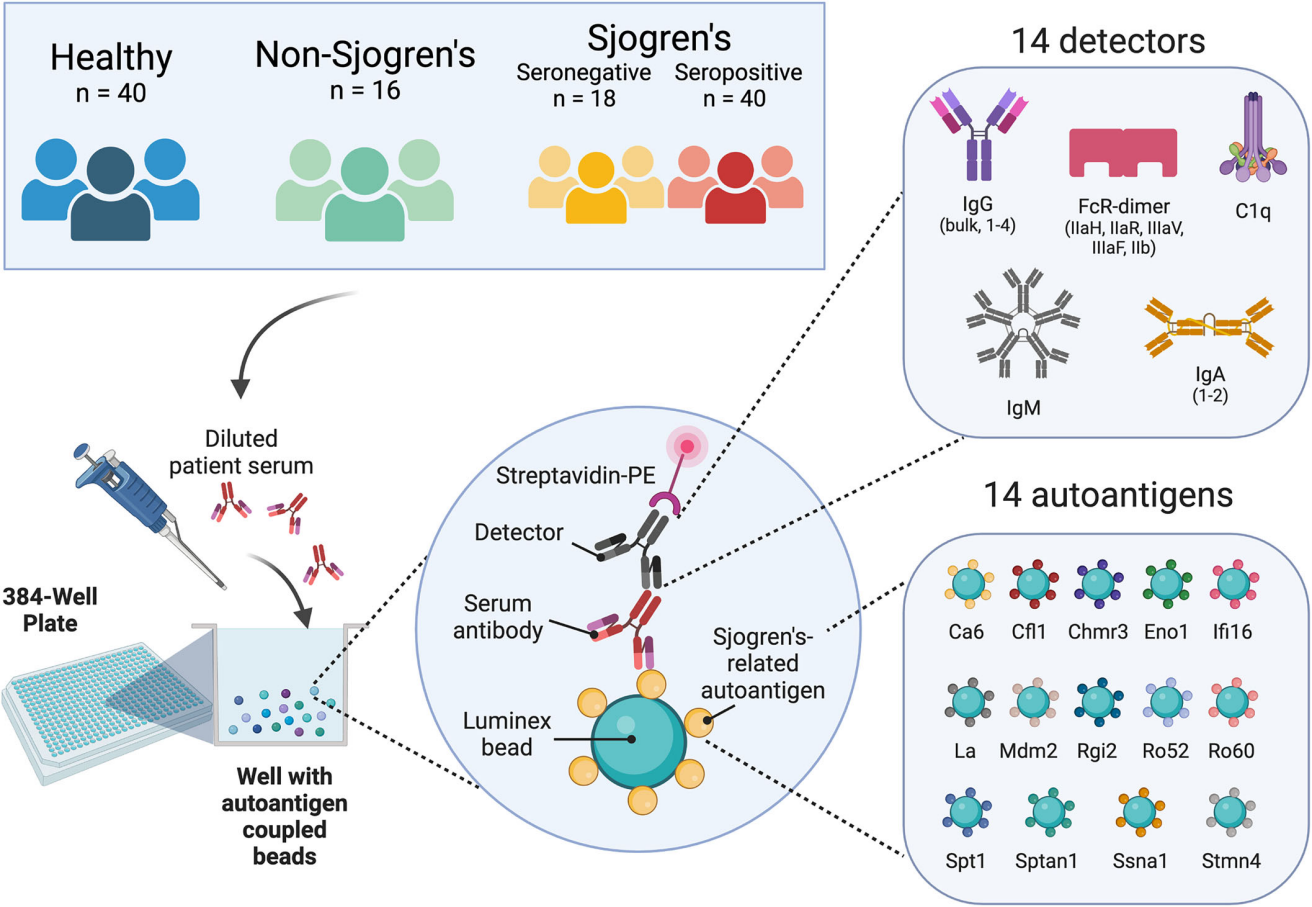
Schematic of bead-based multiplex assay. Patient (Sjögren’s syndrome [SjS] and non-SjS sicca syndrome patients) and control (healthy blood donors) serum are added to wells containing beads coupled to 14 different SjS-related autoantigens. Serum antibodies bind the beads and 14 different detectors directly or indirectly coupled to a fluorophore (phycoerythrin; PE) are added to measure specific autoantibody responses in the serum.

To examine the autoantibody response in SjS versus healthy controls, we designed a multiplex assay as shown in the experimental schematic overview (Fig. 1).

The assay allowed us to assess antibody isotypes (IgG, IgM, IgA), their subclasses (IgG1, IgG2, IgG3, IgG4), C1q binding (classical complement pathway), and FcγR soluble dimer engagement responses to 14 autoantigens relevant to SjS (Ca6, Cfl1, Chmr3, Eno1, Ifi16, La, Mdm2, Rgi2, Ro52, Ro60, Spt1, Sptan1, Ssna1, Stmn4) (Table 1).

**Table 1:** Sjogren’s related autoantigens, their known functions and the reported clinical manifestations in the respective seropositive patients.

Autoantigen	Name	Use	Known function of the protein	Clinical association with the autoantibody	References
Ca6	Carbonic Anhydrase 6	Investigative/non-classical	Ionic exchange in acinar cells, potentially involved in salivary and lacrimal fluid production	Early form of SjS, seronegativity for Ro/La, glandular manifestations	Shen et al, 2012
Cfl1	Cofilin 1	Investigative/non-classical	Regulates organization of the actin cytoskeleton	MALT lymphoma	Cui et al, 2017
Chmr3	Muscarinic receptor M3	Investigative/non-classical	Acetylcholine-dependent glandular secretion	Glandular manifestations	Mona et al, 2021
Eno1	Enolase 1	Investigative/non-classical	Glycolysis, fibrinolysis, immunoglobulin production	MALT lymphoma	Cui et al, 2017
Ifi16	IFN gamma inducible protein 16	Investigative/non-classical	DNA sensor related to IFN pathway and inflammasome, anti-inflammatory	Glandular manifestations	Baer et al, 2016
La/SSB	Sjogren’s Syndrome B	Clinical/classical	RNA protection from degradation	Systemic manifestations, lymphoma	Deroo et al, 2022
Mdm2	Mouse double minute 2 homolog	Investigative/non-classical	Nuclear-localized E3 ubiquitin ligase, binds to p53	Cytopenia, systemic manifestations	Liu et al, 2017
Rgi2	Rho GDP-dissociation inhibitor 2	Investigative/non-classical	Regulates organization of the actin cytoskeleton	MALT lymphoma	Cui et al, 2017
Ro52/SSA/Trim21	Sjogren’s Syndrome A/Tripartite motif containing-21	Clinical/classical	Cytosolic Fc receptor, ubiquitin E3 ligase, type 1 IFN pathway	Systemic manifestations, lymphoma	Deroo et al, 2022
Ro60/SSA/Trove2	Sjogren’s Syndrome A/Trove domain family member 2	Clinical/classical	RNA-binding protein, type 1 IFN pathway	Systemic manifestations, lymphoma	Hung et al, 2015; Deroo et al, 2022
Spt1	Salivary gland protein-1	Investigative/non-classical	Isolated from salivary glands	Early form of SjS, glandular manifestations, seronegativity for Ro/La	Shen et al, 2012
Sptan1	Alpha fodrin	Investigative/non-classical	Movement of the cytoskeleton, interaction with calmodulin	Low specificity, neuropathy, seronegativity for Ro/La	Ulbricht et al, 2003; Zheng et al, 2023
Ssna1/NA14	Sjogren syndrome nuclear autoantigen 1	Investigative/non-classical	Microtubule-stabilizing and damage-sensing protein	Seronegativity for Ro/La	Lawrence et al, 2021
Stmn4	Stathmin 4	Investigative/non-classical	Microtubule-associated protein, role in neurons	Neuropathy	Duda et al, 2017

Overall, 196 autoantibodies-related features (14 antigens × 14 detectors) were measured for 40 healthy controls, 16 patients with non-SjS sicca syndrome and 58 patients with SjS (Table 2).

**Table 2:** Cohort demographics.

Group	*N*	Sex F/M # (%)	Age mean (range)	Time from symptom onset (months) mean (range)	ESSDAI mean (range)	ESSPRI mean (range)	Sialadenitis (Focus Score ≥ 1) *n* (%)
Healthy	40	34/6 (85%)	37.8 (19– 68)	N/A	N/A	N/A	N/A
Non-SjS Sicca syndrome	16	14/2 (87.5%)	53.4 (33– 75)	86 (2–342)	N/A	N/A	2 (12.5%)
Seronegative SjS	18	16/2 (94.3%)	58.7 (28– 84)	98.8 (14–308)	5.6 (0–23)	4.9 (0.3– 10)	18 (100%)
Seropositive SjS	40	36/4 (90%)	57.8 (30– 91)	98.8 (2–477)	5.4 (0–31)	5 (1.3–8.3)	38 (95%)

**Figure 2:**
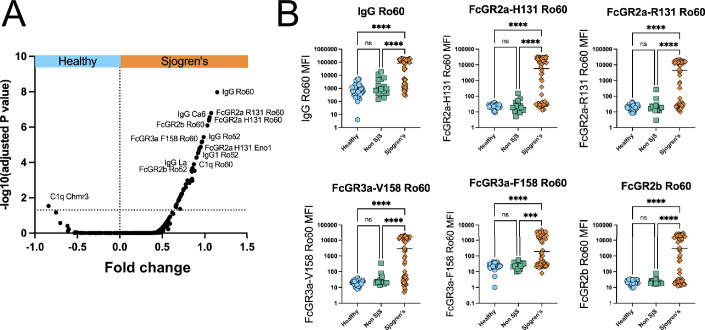
Serologic signature identified for Sjögren’s (SjS) patients. (**A**) Volcano plot of healthy controls (*n* = 40; blue) versus SjS patients (*n* = 58; orange) is presented. Data were z-scored prior to analysis using multiple unpaired *t* test with Welch correction, and the Holm-Sidak method to determine significance, with α  =  0.05 adjusted for 196 tests (autoantibodies-related features). (**B**) Univariate analysis across groups (40 healthy controls in blue, 16 non-SjS sicca syndrome in green, and 58 SjS patients in orange) were performed with multiplex. All significant *P* values were <0.0001 except for the comparison of FcGR3a-F158 Ro60 engagement between non-SjS sicca syndrome and SjS patients whose *P* = 0.0002). Multiplex assays were repeated in duplicates, statistical comparisons were performed using one-way ANOVA (Kruskal–Wallis with Dunn’s multiple comparisons) and significant differences denoted with asterisks (ns = non-significant; *P < 0.05; **P < 0.01; ***P < 0.001; ****P < 0.0001). Source data are available online for this figure.

**Figure EV1:**
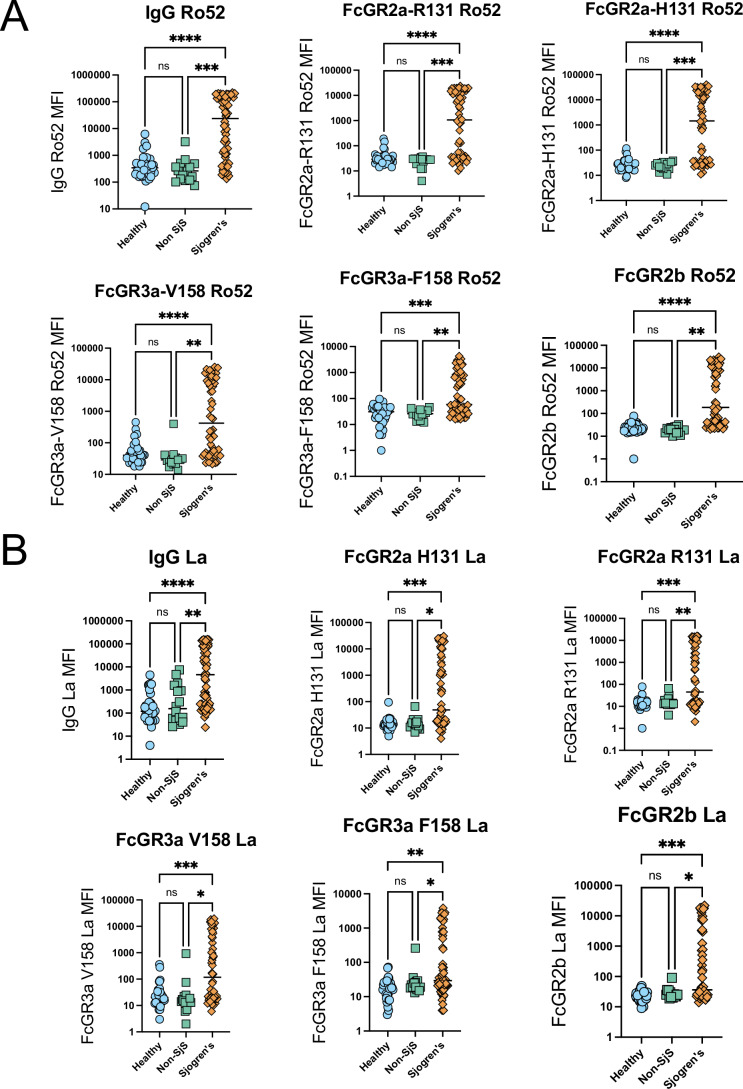
Anti-Ro52 and anti-La specific responses. Univariate analysis across groups (40 healthy controls in blue, 16 non-SjS sicca syndrome in green, and 58 Sjögren’s syndrome [SjS] patients in orange) were performed with multiplex for (**A**) anti-Ro52 and (**B**) anti-La specific responses. Anti-Ro52 features were higher in SjS patients than in non-SjS sicca syndrome patients and healthy controls: anti-Ro52 IgG (*P* = 0.0001 and *P* < 0.0001 respectively), FcGR2a-R131 Ro52 (*P* = 0.0004 and *P* < 0.0001 respectively), FcGR2a-H131 Ro52 (p = 0.0007 and p < 0.0001 respectively), FcGR3a-V158 Ro52 (*P* = 0.0018 and *P* < 0.0001 respectively), FcGR3a-F158 Ro52 (*P* = 0.0099 and *P* = 0.0003 respectively) and FcGR2b Ro52 (*P* = 0.0018 and *P* < 0.0001 respectively) engagements. Anti-La features were higher in SjS patients than in non-SjS sicca syndrome patients and healthy controls: anti-La IgG (*P* = 0.0020 and *P* < 0.0001, respectively), FcGR2a-R131 La (*P* = 0.0077 and *P* = 0.0002, respectively), FcGR2a-H131 La (*P* = 0.0133 and *P* = 0.0004, respectively), FcGR3a-V158 La (*P* = 0.0146 and *P* = 0.0004, respectively), FcGR3a-F158 La (*P* = 0.0293 and *P* = 0.0011, respectively) and FcGR2b La (*P* = 0.0164 and *P* = 0.0006, respectively) engagements. Multiplex assays were repeated in duplicates, statistical comparisons were performed using one-way ANOVA (Kruskal–Wallis with Dunn’s multiple comparisons) and significant differences denoted with asterisks (ns = non-significant; *P < 0.05; **P < 0.01; ***P < 0.001; ****P < 0.0001).

Univariate analysis confirmed previously observed increases in anti-Ro60 (Fig. 2A,B), anti-Ro52 (Fig. EV1A), and anti-La IgG (Fig. EV1B) autoantibodies and revealed novel corresponding elevated FcγR responses in SjS relative to healthy controls (Fig. 2A,B). Of particular interest, high activation of FcγRIIa was also observed, suggesting potential high ADCP to these antigens. Moreover, autoreactive responses to several other explorative non-classical autoantigens (e.g., Ca6 and Eno1) and respective FcγR engagement were identified in SjS patients (Fig. 2A).

**Figure EV2:**
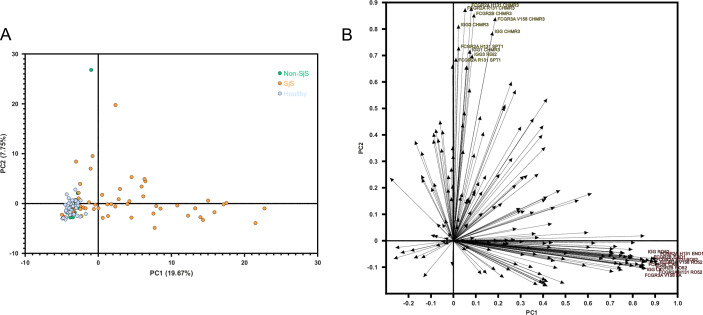
Principal component analysis using the 196 autoantibodies-related features. (**A**) The projection of samples from SjS patients (*n* = 58; orange), healthy controls (*n* = 40; blue), and non-SjS sicca syndrome patients (*n* = 16; green) are presented, and variance explained by each principal component (PC) in parenthesis. SjS patients are correctly separated from healthy controls and non-SjS sicca syndrome patients, mainly across LV1 (top 10 loadings including anti-La FcγRIIa H131, R131, IgG, FcγRIIIa V158, and FcγRIIb; anti-Eno1 FcγRIIa H131 and FcγRIIb; anti- FcγRIIb Ca6; anti-La FcγRIIIa V158 and IgG) and a subset of SjS patients is distinguishable across LV2 (top 10 loadings including anti-Chmr3 FcγRIIa H131, R131, FcγRIIb, FcγRIIIa V158, IgG3, IgG1 and IgG; anti-Rgi2 IgG3; anti-Spt1 FcγRIIa R131, and IgG3). (**B**) The corresponding loadings plot is shown, with the top 10 contributors to PC1 labelled in red, and top 10 contributors to PC2 in yellow. Multiplex assays were repeated in duplicates

A Principal Component Analysis has been performed, as a first unsupervised machine learning approach, using all the 196 autoantibodies-related features of the dataset and including the whole cohort. Projection of samples onto the PCA space revealed a good separation between SjS patients and healthy controls, with patients predominantly distributed along Principal Component 1 (PC1; which accounted for 19.7% of the total variance), and whose main drivers were IgG responses targeting classical antigens (but also Ca6 and Eno1) and the corresponding FcγR engagement. A subset of SjS patients also showed displacement along PC2 (which accounted for 7.8% of the total variance), mainly driven by IgG responses targeting non-classical antigens (Chmr3, Spt1, Cfl1), and FcγR engagement as well. Most healthy controls and non-SjS sicca syndrome patients were clustered together (Fig. EV2A). The corresponding loadings plot for PC1 and PC2 is also shown, with the top 10 contributors for each PC (Fig. EV2B).

**Figure 3:**
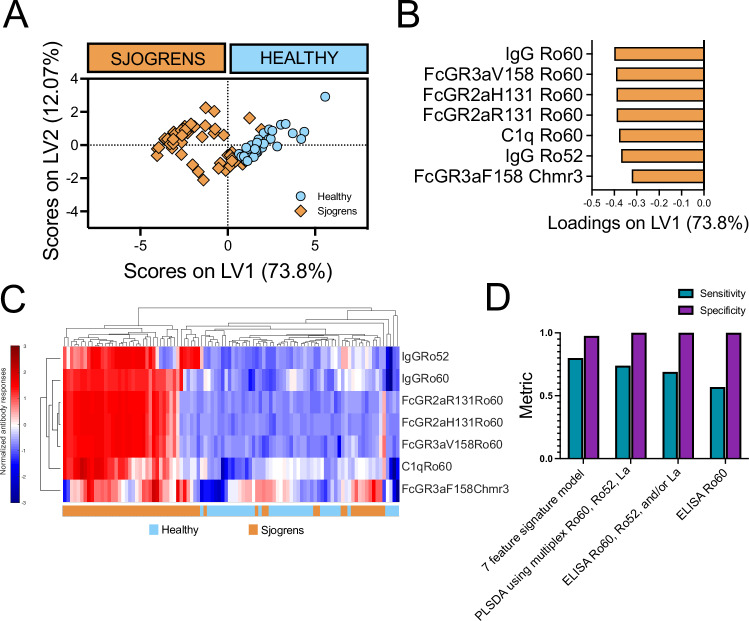
Comparison of autoantibody signatures between Sjögren’s syndrome (SjS) patients and healthy donors. (**A**) PLSDA scores and (**B**) Elastic-net selected feature loadings of SjS patients (*n* = 58; orange) versus healthy controls (*n* = 40; blue) are presented with variance explained by each LV in parentheses. (**C**) Hierarchical clustering was performed using the seven-feature Elastic-Net-selected antibody signature. Data were z-scored prior to analysis. (**D**) Sensitivity and specificity are presented for the 7-feature antibody signature (including the selected features in (**B**)) versus a 3-feature antibody signature (limited to the classical anti-Ro60, anti-Ro52 and anti-La multiplex-based IgG responses) versus clinically-used ELISA-like assay results (either for all 3 classical autoantibodies or just anti-Ro60) for SjS’ diagnosis. Multiplex assays were repeated in duplicates. Source data are available online for this figure.

**Figure EV3:**
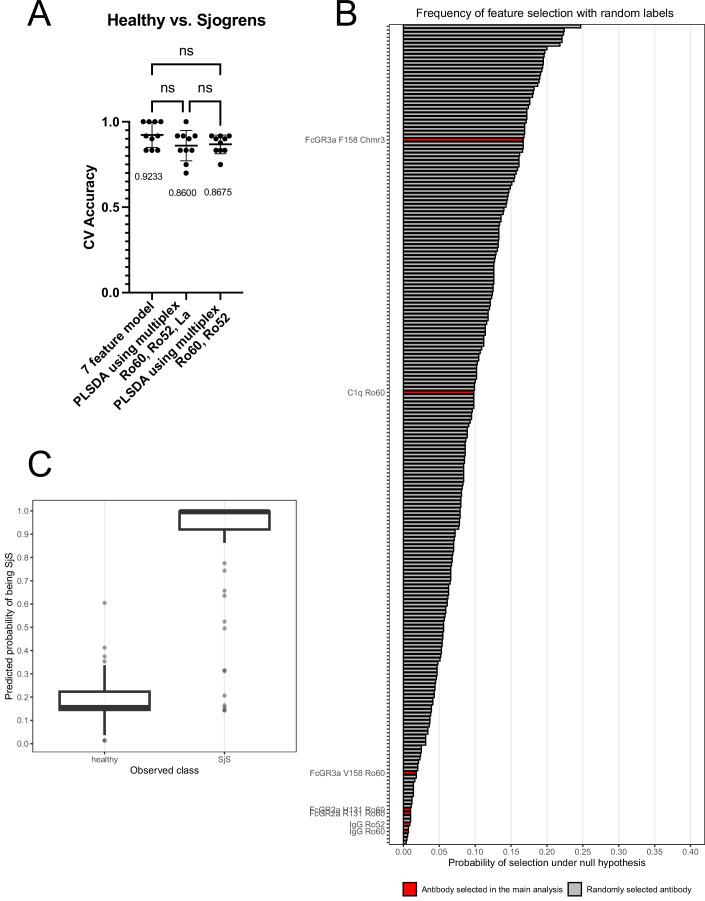
Performances of the 7-feature signature in Sjögren’s syndrome. (**A**) Comparison of cross-validation errors for the Elastic-Net-selected 7-feature antibody signature versus a 3-feature antibody signature (limited to the classical multiplex-based anti-Ro60, anti-La and anti-La IgG responses) versus a signature including only multiplex-based anti-Ro60 and anti-La IgG responses for Sjögren’s syndrome (SjS; *n* = 58) diagnosis compared to healthy controls (*n* = 40). Results are presented as means ± standard deviations. Statistical comparisons were performed using one-way ANOVA (Kruskal–Wallis with Dunn’s multiple comparisons), and significant differences denoted with asterisks (ns = non-significant; *P < 0.05; **P < 0.01; ***P < 0.001; ****P < 0.0001). (**B**) Representation of the selection probabilities for each autoantibody-related feature under the null hypothesis (i.e., after permutation of class labels), to evaluate the performance of the 7-feature model. (**C**) Model calibration analysis evaluating concordance between predicted probabilities of being a SjS patient, and observed frequencies for healthy controls and SjS patients. Multiplex assays were repeated in duplicates.

To identify a minimal autoantibody signature that best distinguished SjD patients from healthy controls, we performed feature selection using Elastic-Net regularization followed by a supervised classification method (PLSDA). The identified signature included 7 autoantibodies-related features and was able to accurately discriminate between SjS patients and healthy controls with 91.4% calibration and 92.2% cross-validation accuracy. Interestingly, the signature included C1q, FcγRIIa-H131/R131 and FcγRIIIa-V158 engagement specific to Ro60 and Ro60- and Ro52-specific IgG (Fig. 3A,B). Moreover, hierarchical clustering showed good separation between healthy controls and SjS patients based on the 7-feature antibody signature identified (Fig. 3C). Sensitivity and specificities (Fig. 3D) were calculated from model performance for this 7-feature antibody signature, with 80.0% sensitivity and 97.5% specificity, and for a PLSDA model including only multiplex-based IgG responses to all 3 classical antigens (i.e., Ro60, Ro52 and La) with 73.9% sensitivity and 100% specificity. Comparatively, seropositivity for any of the classical autoantibody (either anti-Ro60, anti-Ro52, or anti-La IgG), based on ELISA-like assay results, had a sensitivity of 69.0% (*n* = 40) and seropositivity for Ro60 (based on ELISA-like assay as well) resulted in a sensitivity of 56.9% (*n* = 33) within our cohort. When using multiplex assay data, 77.6% (*n* = 45) patients with SjS were considered seropositive, with only one (2.5%) healthy individual positive for anti-La and another positive for anti-Ro52 (compared to none with ELISA-like assay). Calibration accuracy of our seven-feature signature was 91.4% and cross-validation accuracy was 92.3% compared to 86.0% and 86.8%, respectively, for the PLSDA model including only multiplex-based IgG responses to Ro60, Ro52 and La) and for a PLSDA model including only multiplex-based IgG responses to Ro60 and Ro52 (Fig. EV3A). The 7-feature autoantibody model performance was analyzed using a permutation test (i.e., by shuffling the class labels and rerunning the full pipeline repeatedly to assess whether the identified features were selected beyond chance levels), without clear signs of overfitting (Fig. EV3B). Moreover, a model calibration analysis reported a good concordance between predicted probabilities and observed frequencies for healthy controls and SjS patients (Fig. EV3C).

### Unique autoantibody responses between non-Sjögren’s sicca syndrome and Sjögren’s syndrome patients differentiate these individuals from healthy controls

**Figure 4:**
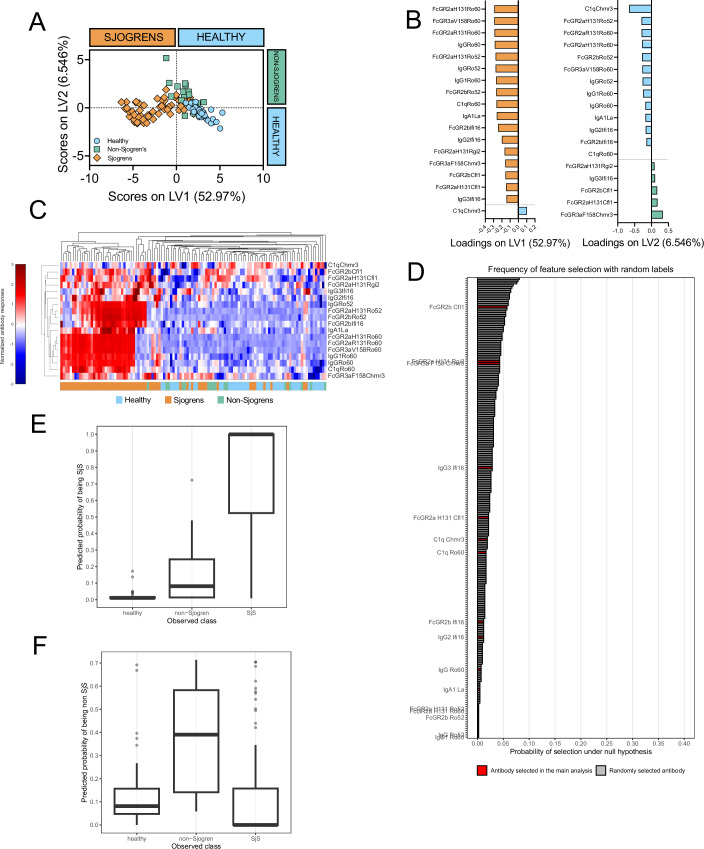
Comparison of autoantibody signatures between Sjögren’s syndrome (SjS) patients, non-SjS sicca syndrome patients and healthy donors. (**A**) PLSDA scores and (**B**) Elastic-net selected feature loadings of SjS patients (*n* = 58; orange), healthy controls (*n* = 40; blue), and non-SjS sicca syndrome patients (*n* = 16; green) are presented with variance explained by each LV in parentheses. Scores plot shows separation of SjS patients from healthy controls and non-SjS sicca syndrome patients across LV1 while healthy controls and non-SjS sicca syndrome patients are distinguishable across LV2. (**C**) Hierarchical clustering was performed using Elastic-Net-selected antibody features. Data were z-scored prior to analysis. (**D**) Representation of the selection probabilities for each autoantibody-related feature under the null hypothesis (i.e., after permutation of class labels), to evaluate the performance of the model. Model calibration analysis evaluating concordance between predicted probabilities of being (**E**) a SjS, or (**F**) a non-SjS sicca syndrome patient, and observed frequencies for healthy controls, non-SjS sicca syndrome and SjS patients. Multiplex assays were repeated in duplicates. Source data are available online for this figure.

We next investigated differences in autoantibody signatures between individuals with SjS and non-SjS sicca syndrome patients. Univariate analysis observed many non-significant differences in antibody responses between the non-SjS sicca syndrome group and healthy controls (Fig. 2B). In comparing minimal multivariate signatures using the feature selection/PLSDA analysis pipeline described above, results indicated that non-SjS sicca syndrome patients had autoantibody signatures more similar to healthy controls than individuals with SjS, as indicated by separation across latent variable 1 (LV1) (Fig. 4A,B). As for previous analyses, SjS was associated with anti-Ro60 and anti-Ro52 IgG, FcγRIIa, IIb, and IIIa engagement. Interestingly, the results indicated that the non-SjS sicca syndrome patients separated from the healthy population across the second LV2, which involved increases in non-classical autoantibodies-related features including anti-Chmr3, anti-Cfl1, anti-Rgi2, and anti-Ifi16 (Fig. 4A,C). In line with this, 25% (*n* = 4/16) of the non-SjS sicca syndrome patients (who are all seronegative according to ELISA-like assay data) were IgG-positive to at least one of the non-classical autoantigens (Chmr3, Cfl1, Rgi2, Ifi16 and/or Ca6) according to multiplex assay data, including two who were not positive for any other autoantibody, and with that addition both would be “reclassified” as SjS according to the international criteria. Despite not being identified as major drivers of the non-SjS sicca syndrome patients’ autoantibody signature via the feature selection/PLSDA analysis pipeline, 25% (*n* = 4/16) individuals in this group were positive for anti-Ro60 IgG, and 12.5% (*n* = 2/16) for anti-La IgG, compared to 0% and 2.5% (*n* = 1/40) of the healthy controls, respectively, likely due to these features mainly driving LV1.

When including multiplex assay data regarding classical (i.e., anti-Ro60 and anti-La, as none were anti-Ro52 IgG positive) and non-classical (i.e., anti-Cfl1, anti-Rgi2, anti-Spt1, anti-Ca6, anti-Chmr3) IgG autoantibody positivity into account, a total of eight non-SjS sicca syndrome patients would be considered seropositive, including 75% (*n* = 6/8) who would be “reclassified” as SjS according to the international criteria. Interestingly, low levels of corresponding autoantigen-specific FcγR binding were detected in 66.7% of these six patients, but none of the other non-SjS sicca syndrome patients with multiplex assay-only autoantigen IgG positivity. Hierarchical clustering enabled visualization of clear separation of SjS patients from healthy and non-SjS sicca syndrome individuals using the LV1 feature-selected antibody signature (Fig. 4C). The model performance was also analyzed using the previously described permutation test, without clear signs of overfitting either (Fig. 4D). Moreover, the model calibration analysis reported good concordances between predicted probabilities of being a SjS (Fig. 4E) or a non-SjS sicca syndrome patient, and the observed frequencies for healthy controls, non-SjS sicca syndrome and SjS patients (Fig. 4F).

### N-linked glycosylation profiling reveals variations in IgG glycans associated with Sjögren’s syndrome

**Figure 5:**
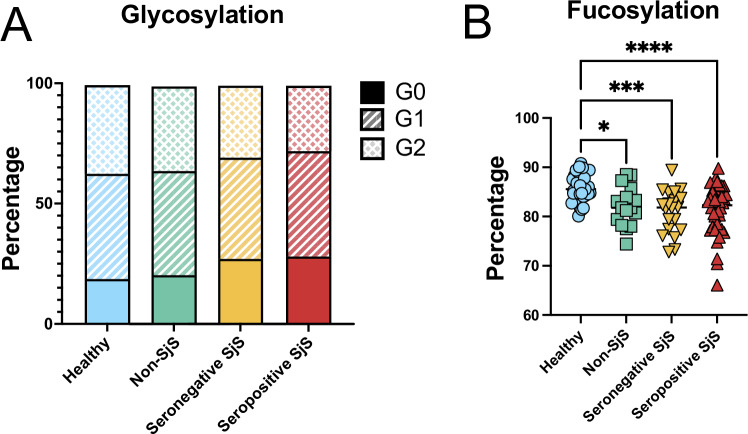
Total IgG glycosylation profiling. Total IgG (**A**) galactosylation profiles (G0 = no galactose residue; G1 = 1 galactose residue; G2 = 2 galactose residues) are shown in seropositive (i.e., positive for anti-Ro/SSA and/or anti-La/SSB according to clinically-used ELISA-like assay results) Sjögren’s syndrome (SjS) patients (*n* = 40; red), seronegative SjS patients (*n* = 18; yellow), non-SjS sicca syndrome patients (*n* = 16; green) and healthy controls (*n* = 40; blue). (**B**) Total IgG fucose residue relative abundance were compared in the same groups: seropositive SjS (*P* < 0.0001), seronegative SjS (*P* = 0.0001), and non-SjS sicca syndrome patients (*P* = 0.0103) having lower fucose residue relative abundance than healthy controls. Glycosylation profiling were repeated in duplicates, statistical comparisons were performed using one-way ANOVA (Kruskal–Wallis with Dunn’s multiple comparisons) and significant differences denoted with asterisks (ns = non-significant; *P < 0.05; **P < 0.01; ***P < 0.001; ****P < 0.0001). Source data are available online for this figure.

**Figure EV4:**
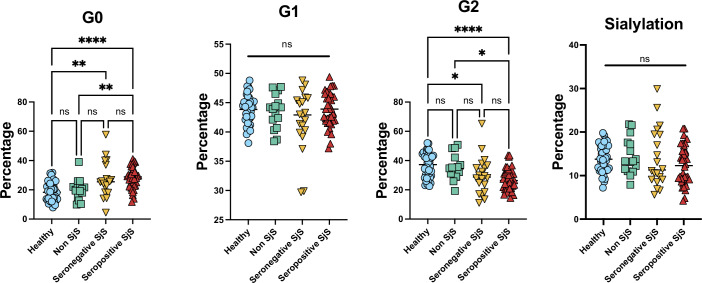
Total IgG galactosylation and sialylation profiling. Comparisons of the relative abundance of the different galactosylation profiles (G0 = no galactose residue; G1 = 1 galactose residue; G2 = 2 galactose residues) and sialylation of total IgG were performed between seropositive (i.e., positive for anti-Ro/SSA and/or anti-La/SSB according to clinically-used ELISA-like assay results) Sjögren’s syndrome (SjS) patients (*n* = 40; red), seronegative SjS patients (*n* = 18; yellow), non-SjS sicca syndrome patients (*n* = 16; green) and healthy controls (*n* = 40; blue). Total IgG G0 residue relative abundance was higher in seropositive SjS patients compared to non-SjS sicca syndrome patients (*P* = 0.0075) and healthy controls (*P* < 0.0001). Total IgG G0 residue relative abundance was also higher in seronegative SjS patients compared to healthy controls (*P* = 0.0012). Total IgG G2 residue relative abundance was lower in seropositive SjS patients compared to non-SjS sicca syndrome patients (*P* = 0.0172) and healthy controls (*P* < 0.0001). Total IgG G2 residue relative abundance was also lower in seronegative SjS patients compared to healthy controls (*P* = 0.0307). Glycosylation profiling were repeated in duplicates, statistical comparisons were performed using one-way ANOVA (Kruskal–Wallis with Dunn’s multiple comparisons) and significant differences denoted with asterisks (ns = non-significant; *P < 0.05; **P < 0.01; ***P < 0.001; ****P < 0.0001).

As autoantigen-specific FcγRIIa, FcγRIIb and FcγRIIIa binding was identified as an important contributor to the autoantibody signatures associated with SjS, we examined total IgG N-linked glycosylation, a known important factor in the modulation of the strength of IgG-Fc/FcγR engagement, with a special focus on seropositive (i.e., either anti-Ro60, anti-Ro52, and/or anti-La IgG-positive based on ELISA-like assay results) and seronegative patients, among the SjS group. Regarding galactosylation (Fig. 5A), total IgG from seropositive SjS patients had the higher amount of G0 (i.e., ungalactosylated) IgG (28.3% ± 1.3), then seronegative SjS patients (25.3% ± 2.8), non-SjS sicca syndrome patients (20.4% ± 1.8) and healthy controls (18.6% ± 1.0). The increase in G0 relative abundance in seropositive SjS patients was statistically significant when compared to non-SjS sicca syndrome patients (*P* = 0.0036) and healthy controls (*P* < 0.0001), whereas seronegative SjS were only significantly increased compared to healthy controls (*P* = 0.0446) (Fig. EV4). Accordingly, the relative abundance of highly galactosylated IgG (referred to as G2) followed the exact opposite trend in the three groups. Healthy controls had significantly higher amount of fucosylated IgG (classically associated with reduced FcγRIII binding, compared to afucosylated IgG) compared to all the other groups, including the non-SjS sicca syndrome group (Fig. 5B).

### Cell-based phagocytosis assay suggests autoantibody-associated FcγR binding is linked to elevated autoantigen-specific phagocytosis in Sjögren’s syndrome

**Figure 6:**
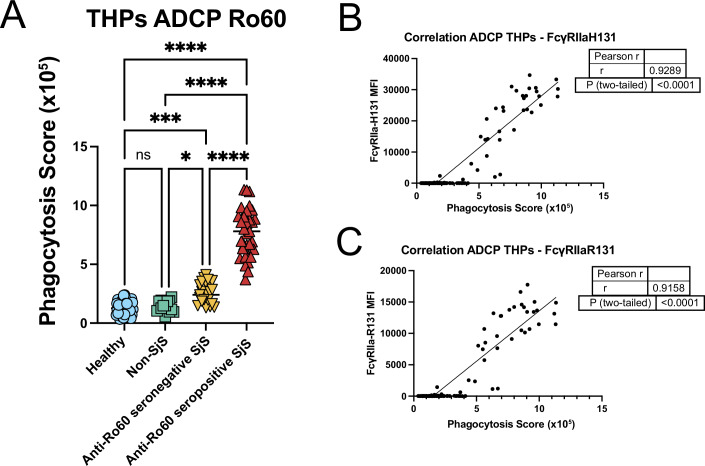
THP-1 Ro60-coated bead-based antibody-dependent phagocytosis assay. (**A**) Comparisons of anti-Ro60 specific antibody-dependent cellular phagocytosis were performed in anti-Ro60 IgG seropositive (according to anti-Ro60 IgG ELISA-like assay) SjS patients (*n* = 33, red), anti-Ro60 IgG seronegative patients (*n* = 25, yellow), non-SjS sicca syndrome patients (*n* = 16; green) and healthy controls (*n* = 40; blue), using the THP-1 monocyte cell line antibody-mediated uptake of Ro60-coated bead assay. Phagocytosis scores were calculated by multiplying the percentage of Ro60-coated-bead-positive cells and their median fluorescence intensity. Anti-Ro60 IgG seropositive SjS patients had higher phagocytosis scores (× 10^5^) than all the other groups (*P* < 0.0001). Anti-Ro60 IgG seronegative SjS patients had higher phagocytosis scores than non-SjS sicca syndrome patients (*P* = 0.0347) and healthy donors (*P* = 0.0011). Correlation analyses (measured by Pearson correlation) with anti-Ro60 (**B**) FcγRIIa-H131 (*P* < 0.0001) and (**C**) FcγRIIa-R131 (*P* < 0.0001) dimers engagement in multiplex assay were performed in the whole cohort. THP-1 bead-based phagocytosis and multiplex assays were repeated in duplicates, statistical comparisons were performed using one-way ANOVA (Kruskal–Wallis with Dunn’s multiple comparisons) and significant differences denoted with asterisks (ns = non-significant; *P < 0.05; **P < 0.01; ***P < 0.001; ****P < 0.0001). Source data are available online for this figure.

As FcγRIIa (both the H131 and R131 polymorphisms) binding of anti-Ro60 autoantibodies was a critical feature associated within all SjS-associated autoantibody signatures, we evaluated their functionality via an anti-Ro60 bead-based ADCP assay (Fig. 6A). Anti-Ro60 IgG seropositive (according to ELISA-like assay) SjS patients (*n* = 33 anti-Ro60 seropositive SjS patients out of the 40 SjS patients who are seropositive for anyone of the 3 classical autoantibodies) had higher phagocytosis scores (×10^5^) among all groups, with a mean score of 7.7 ± 0.4 versus 2.5 ± 0.2 (*P* < 0.0001) for anti-Ro60 IgG seronegative SjS patients (*n* = 25), 1.4 ± 0.1 (*P* < 0.0001) for non-SjS sicca syndrome patients (*n* = 16) and 1.3 ± 0.1 (*P* < 0.0001) for healthy controls (*n* = 40). Anti-Ro60 IgG seronegative SjS patients also had higher phagocytosis scores than non-SjS sicca syndrome patients (*P* = 0.0347) and healthy donors (*P* = 0.0011), whose respective phagocytosis scores were however not statistically different (*P* = 0.98). In comparison, SjS patients who were anti-Ro52 IgG only (*n* = 6) or anti-La IgG only (*n* = 1) positive, without anti-Ro60 IgG positivity, had low phagocytosis scores, comparable with seronegative patients (i.e., a mean phagocytosis score of 2.3 ± 0.3 and a phagocytosis score of 3.6, respectively). Finally, phagocytosis scores from the whole cohort were significantly correlated with FcγRIIa-H131 (Pearson correlation coefficient *r* = 0.93; *P* < 0.0001) and FcγRIIa-R131 (*r* = 0.92; *P* < 0.0001) specific engagement of anti-Ro60 autoantibodies (Fig. 6B,C), but also with anti-Ro60 total IgG (*r* = 0.91), and FcγRIIIa-V158 (*r* = 0.93), FcγRIIIa-F158 (*r* = 0.87) and FcγRIIb (*r* = 0.91) specific engagement (all *P* < 0.0001). The correlation was not as strong for anti-Ro60 IgG2 (*r* = 0.42) and IgG4 (*r* = 0.38; both *P* < 0.0001), known for their limited ability to engage effector functions.

### Seronegative SjS patients can be differentiated from healthy controls based on an autoantibody signature driven by non-classical antigens

**Figure EV5:**
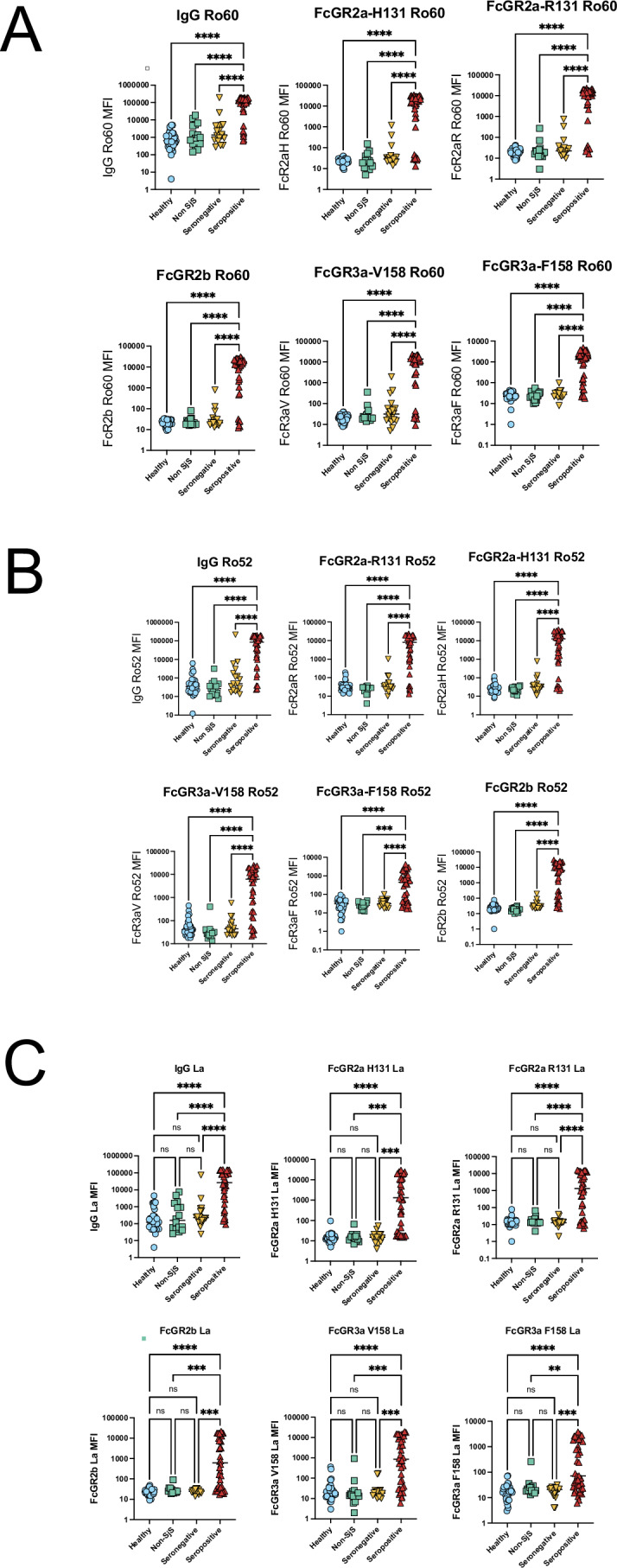
Anti-Ro60, anti-Ro52 and anti-La specific responses depending on the serostatus in Sjögren’s syndrome. Univariate analysis across seropositive (i.e., positive for anti-Ro/SSA and/or anti-La/SSB according to clinically-used ELISA-like assay results) Sjögren’s syndrome (SjS) patients (*n* = 40; red), seronegative SjS patients (*n* = 18; yellow), non-SjS sicca syndrome patients (*n* = 16; green) and healthy control (*n* = 40; blue) groups were performed with multiplex for (**A**) anti-Ro60, (**B**) anti-Ro52 and (**C**) anti-La specific responses. All anti-Ro60 and anti-Ro52 features (specific IgG, and specific engagements of FcGR2a-R131, FcGR2a-H131, FcGR3a-V158, FcGR3a-F158 and FcGR2b) were higher in seropositive SjS patients than in seronegative SjS, non-SjS sicca syndrome patients and healthy controls (all *P* < 0.0001). Anti-La features were higher in seronegative SjS patients than in seronegative SjS, non-SjS sicca syndrome patients and healthy controls: anti-La IgG (*P* < 0.0001 for the 3 comparisons), FcGR2a-R131 La (*P* < 0.0001 for the 3 comparisons), FcGR2a-H131 La (*P* = 0.0001, *P* = 0.0002, and *P* < 0.0001 respectively), FcGR3a-V158 La (*P* = 0.0001, *P* = 0.0002, and *P* < 0.0001, respectively), FcGR3a-F158 La (*P* = 0.0004, *P* = 0.0010, and *P* < 0.0001, respectively) and FcGR2b La (*P* = 0.0002, *P* = 0.0003, and *P* < 0.0001, respectively) engagements. Multiplex assays were repeated in duplicates, statistical comparisons were performed using one-way ANOVA (Kruskal–Wallis with Dunn’s multiple comparisons) and significant differences denoted with asterisks (ns = non-significant; *P < 0.05; **P < 0.01; ***P < 0.001; ****P < 0.0001).

**Figure 7:**
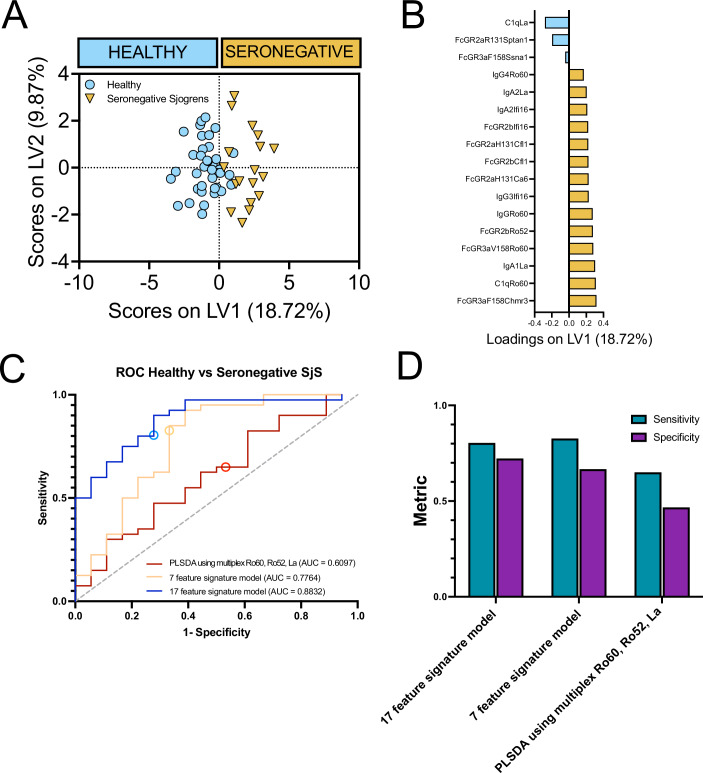
Comparison of autoantibody signatures between seronegative Sjögren’s syndrome (SjS) patients and healthy donors. (**A**) PLSDA scores and (**B**) loadings of seronegative (i.e., negative for anti-Ro/SSA and anti-La/SSB according to clinically-used ELISA-like assay results) SjS patients (*n* = 18; light orange) versus healthy controls (*n* = 40; blue) are presented with variance explained by each LV in parentheses. (**C**) Area Under the Curve (AUC) of Receiver operating characteristic (ROC) curves, as well as (**D**) sensitivity and specificity, are presented for the Elastic-Net-selected 17-feature “seronegative SjS” antibody signature versus the 7-feature signature, versus a 3-feature antibody signature (limited to the classical multiplex-based anti-Ro60, anti-Ro52 and anti-La IgG responses) for SjS diagnosis in seronegative SjS. Multiplex assays were repeated in duplicates Source data are available online for this figure.

A major challenge in SjS diagnosis remains recognizing individuals who are not seropositive for classical antigens, requiring performing an invasive minor salivary gland biopsy to meet the international classification criteria for SjS. Thus, we wanted to explore what autoantibodies might drive this difference between healthy controls and seronegative SjS patients. Univariate results revealed significantly higher anti-Ro60 (Fig. EV5A), Ro52 (Fig. EV5B), and La (Fig. EV5C) responses in seropositive SjS patients compared to seronegative SjS patients, but the differences between healthy controls and seronegative SjS was non-significant. Using feature selection and PLSDA, we were able to identify an autoantibody signature that successfully distinguished between healthy individuals and seronegative patients (Fig. 7A). The 17 selected features in this model included some anti-Ro60, anti-Ro52, and anti-La autoantibody-related features, such as anti-Ro60 IgG, anti-Ro52 FcγRIIb, and anti-Ro60 FcγRIIIa-V158 (Fig. 7B). In line with this, based on multiplex assay instead of ELISA assay data, some patients would have been considered seropositive among the seronegative group: three for anti-Ro60 IgG, three for anti-Ro52 IgG and two for anti-La IgG.

**Figure EV6:**
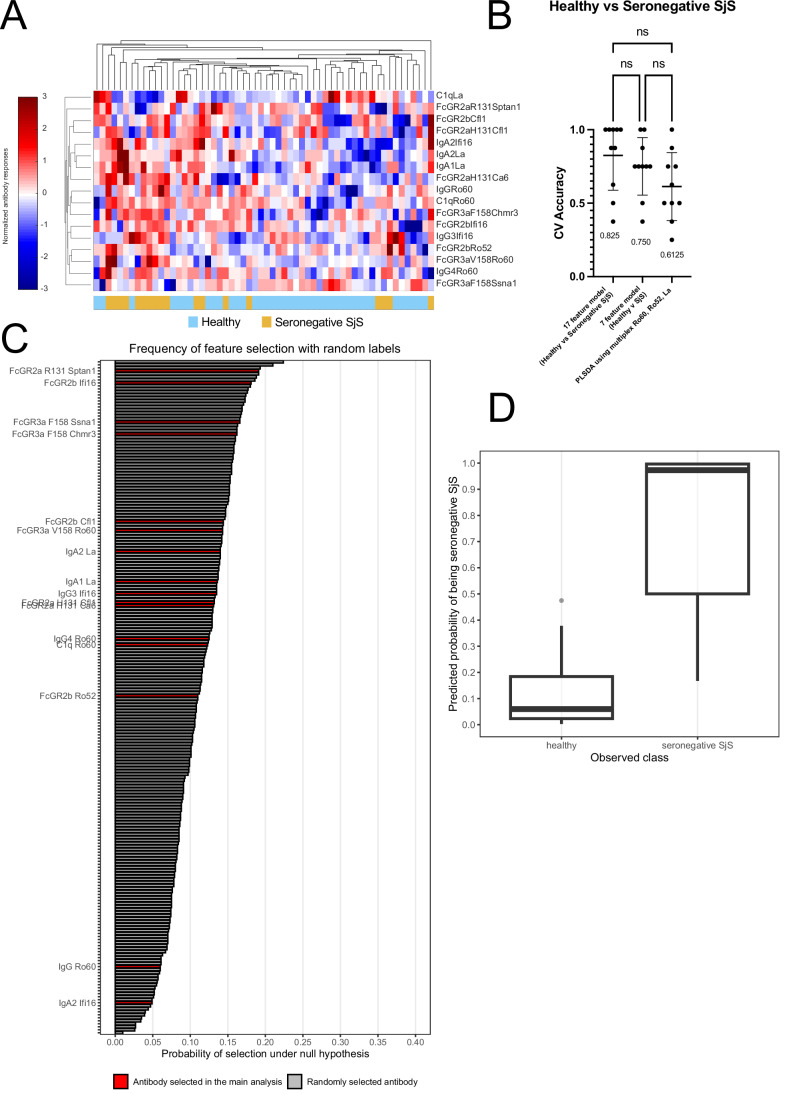
Performances of the 17-feature signature in seronegative Sjögren’s syndrome. (**A**) Hierarchical clustering was performed using the Elastic-Net-selected 17-feature signature identified by comparing seronegative (i.e., negative for anti-Ro/SSA and anti-La/SSB according to clinically-used ELISA-like assay results) Sjögren’s syndrome (SjS) patients (*n* = 18; light orange) versus healthy controls (*n* = 40; blue). Data were z-scored prior to analysis. (**B**) Comparison of cross-validation errors for the Elastic-Net-selected 17-feature “seronegative SjS” antibody signature versus the 7-feature signature, versus a 3-feature antibody signature (limited to the classical multiplex-based anti-Ro60, anti-La and anti-La IgG responses) for SjS diagnosis in seronegative SjS. Results are presented as means ± standard deviations. Statistical comparisons were performed using one-way ANOVA (Kruskal–Wallis with Dunn’s multiple comparisons), and significant differences denoted with asterisks (ns = non-significant; *P < 0.05; **P < 0.01; ***P < 0.001; ****P < 0.0001). (**C**) Representation of the selection probabilities for each autoantibody-related feature under the null hypothesis (i.e., after permutation of class labels), to evaluate the performance of the 17-feature model. (**D**) Model calibration analysis evaluating concordance between predicted probabilities of being a seronegative SjS patient, and observed frequencies for healthy controls and seronegative SjS patients. Multiplex assays were repeated in duplicates.

Interestingly, there are also many non-classical autoantibody-related features that are part of the 17-feature signature, including anti-Ifi16 IgG3, anti-Ca6 FcγRIIa-H131, and anti-Cfl1 FcγRIIb and FcγRIIa-H131 engagement. This suggests that elevated autoantibodies to non-classical antigens may be important features in seronegative SjS patients. By plotting ROC curves (Fig. 7C), we were able to show the good sensitivity and specificity of the 17-feature autoantibody signature in comparison to a PLSDA model including only multiplex-based IgG responses to all 3 classical antigens (i.e., Ro60, Ro52 and La), and the seven-feature signature previously identified by comparing healthy donors versus SjS patients (Fig. 3). Hierarchical clustering between healthy controls and seronegative SjS patients based on the 17-feature antibody signature identified is shown in Fig. EV6A. Our model had a cross-validation accuracy of 82.5% in comparison to cross-validation accuracies of 75.0% using the seven-feature signature and 61.3% using the PLSDA model including only multiplex-based anti-Ro60, Ro52 and La IgG responses (Fig. EV6B). The 17-feature autoantibody model performance was analyzed using the permutation test, and identified some degree of overfitting (Fig. EV6C). On the other hand, the model calibration analysis reported a good concordance between predicted probabilities and observed frequencies for healthy controls and seronegative SjS patients (Fig. EV6D). The sensitivities and specificities for each model are shown in Fig. 7D, with 80.4% sensitivity and 72.2% specificity for the 17-feature signature, 82.7% sensitivity and 66.7% specificity for the 7-feature signature, and 65% sensitivity and 46.7% specificity for the PLSDA model with only Ro60, Ro52 and La.

**Figure EV7:**
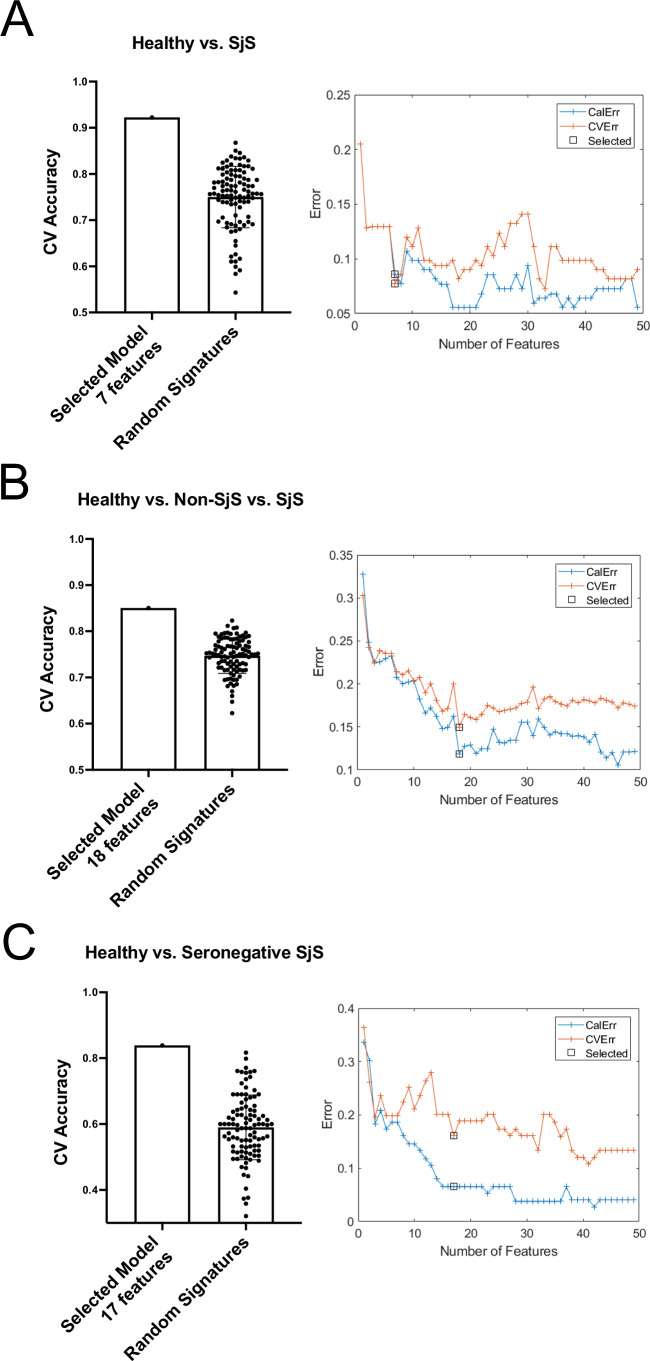
Performances of the 3 main selected signatures. Presentation of the calibration and cross-validation accuracy for the three identified autoantibody signatures (left) in (**A**) Sjögren’s syndrome (SjS) patients and healthy donors, (**B**) SjS patients, non-SjS sicca syndrome patients and healthy donors, or (**C**) seronegative SjS patients and healthy donors to 100 randomly generated signatures with the same number of features, and the respective charts (right) showing the selected features.

Additional description of the performance (calibration and cross-validation accuracies) of the three main identified autoantibody signatures are presented in Fig. EV7A,B,C.

## Discussion

In this study, we have applied a novel SjS-dedicated systems serology approach to study differences in autoantibody responses between SjS patients, non-SjS sicca syndrome patients and healthy controls. Systems serology approaches have almost exclusively been applied in the field of infectious diseases to date (Chung et al, 2015; Arnold and Chung, 2018; Chung and Alter, 2017) but have the ability to provide new insight in understanding complex autoantibody responses and improve autoimmune patients’ care (Chung et al, 2020; Selva et al, 2021a; McLean et al, 2021).

Without any surprise, we confirm the key role of anti-Ro60/SSA, anti-Ro52/SSA and anti-La/SSB IgG autoantibodies in SjS, but we have also identified high levels of corresponding FcγR and C1q engagement. Fc-related effector functions have been scarcely studied in autoimmune diseases (Chalayer et al, 2022), and never in SjS at a system level. In this study, FcγR binding has been identified as prominent components of all autoantibody signatures associated with SjS. The inclusion of FcγR and C1q engagement, along with IgG measurement, as in the identified SjS-associated seven-feature autoantibody signature, seemed to have a higher sensitivity for SjS diagnosis compared to the routinely used ELISA-like assay, but it will need to be confirmed in dedicated studies. Moreover, as anti-Ro60-associated autoantibody features were the most represented in this seven-feature signature (accounting for 5 of them), but also in the other more complex signatures presented here, such an assay could eventually be adapted for clinical settings (contrary to its current version) with a curated set of autoantigens, including Ro60, potentially alleviating the need for patient biopsies in some cases. Moreover, in line with the literature (Bizzaro et al, 2022), bead-based multiplex instead of classical solid phase ELISA or ELISA-like technology has been associated with improved sensitivity for classical autoantibodies’ detection (+ 8.6% anti-Ro/SSA and/or anti-La/SSB seropositivity in the SjS group), again possibly allowing for quicker and less invasive diagnostic procedures, without significant loss of specificity. Accordingly, multiplex-associated improved sensitivity for classical autoantibodies’ detection would allow for the reclassification of 4 non-SjS sicca syndrome patients (25% of the group) and none of the healthy individuals, as having SjS according to at least one set of international classification criteria (Shiboski et al, 2017; Vitali et al, 2002).

Apart from the classical autoantibodies, the SjS-dedicated custom multiplex assay from this study was designed to include several previously described non-classical autoantibodies, to evaluate their potential value in identifying SjS and non-SjS patients. Multiple non-classical autoantibodies (anti-Chmr3, anti-Cfl1, anti-Rgi2, anti-Ifi16 and/or anti-Ca6) were included in all signatures associated with SjS, but also in non-SjS sicca syndrome patients, with two (12.5%) additional individuals who would be “reclassified” as SjS when taking non-classical autoantibodies’ data into account, but their diagnostic’ value will need further evaluation as well. Bridging the “seronegative gap” has been a key goal in SjS—and more globally in systemic autoimmune diseases—for many years, with different approaches, including the use of human proteome assays in recent publications (Longobardi et al, 2023). Such approach allows for wider autoantigen screening, such as the proteome array comprising 19,500 proteins that cover >75% of the human proteome, that identified 11 novel SjS autoantibodies with low individual frequencies (using the same threshold determining method as we did), as we similarly found for non-classical autoantibodies in this study. In contrast to proteomic approaches, current multiplex bead technologies are limited to a maximum of 500 antigens but are more rapid and easily portable to the clinical setting. This approach also allowed for the simultaneous evaluation of the autoantigen-specific FcγR engagement, which were identified as prominent for classical autoantibodies but also detected in more than 2/3 of the non-classical autoantibody-seropositive patients in our cohort. Using similar machine learning approaches, Longobardi et al (Longobardi et al, 2023) have identified a 30-feature (only including autoantibodies-specificities) signature which was able to identify seronegative SjS from healthy individuals, with an AUC of 0.79 (95% CI 0.64–0.93), compared to our 17-feature signature (including both Fab and Fc-related features) whose AUC was 0.88 (95% CI 0.79–0.97) for the same purpose. Regarding autoantibodies’ specificities, there were overlapping antigens between both approaches, as they also detected anti-Cfl1 and anti-Stmn4 in their cohort, but others were not, either because the autoantigens were absent from the array (e.g., Spt1, Sptan1) or because of technical differences in the detection methods, especially protein conformation issues as raised by the authors themselves (Longobardi et al, 2023). Due to its very nature, the SjS-dedicated custom multiplex assay we have designed and evaluated in this study used native or recombinant proteins, without conformational issues, however its intrinsic qualities are necessarily highly dependent on the protein quality themselves.

As FcγR engagement was associated with all the identified autoantibodies and as an important feature in all the selected signature in our cohort, we investigated the potential role of IgG N-linked glycosylation, a well-known factor in modulating IgG Fc binding to their receptors (Chung et al, 2014; Damelang et al, 2024; Dekkers et al, 2018; Yamaguchi and Barb, 2020). Previous SjS-dedicated total antibody glycosylation studies are more than 30-year-old and few, but have observed decreases in sialylation (Youinou et al, 1992), which was not observed in our cohort, and decreases in galactosylation (Parekh et al, 1989). Reduced levels of total IgG galactosylation in both seronegative and seropositive SjS patients in this study is in line with previous publications, along with a range of autoimmune diseases and thought to be reflective of their common proinflammatory state (Seeling et al, 2017; Dekkers et al, 2018; Flevaris and Kontoravdi, 2022). Counterintuitively, it has been experimentally demonstrated that antibody galactosylation, was significantly increasing the affinity for FcγRIIIa (at least in afucosylated antibodies) (Dekkers et al, 2018; Damelang et al, 2024) and C1q activation, hence the in vivo real impact of antibody galactosylaton through FcγR/IgG binding, is impossible to settle without comprehensive data about antigen-specific antibody glycosylation (which could not be performed here due to limited available sample volumes), along with total IgG data which can compete for the same receptors (Dekkers et al, 2018). Increased binding of afucosylated IgG to FcγR (especially FcγRIIIa) has also been well demonstrated, leading to beneficial or detrimental effects (particularly in autoimmune haemolytic and thrombocytopenic diseases) depending on the setting (Oosterhoff et al, 2022), and interestingly fucosylation of total IgG was the lowest in seropositive SjS patients in our cohort. Moreover, seronegative SjS and non-SjS sicca syndrome patients also had lower levels of IgG fucosylation than healthy controls. This is in line with our observation of higher FcγR engagement to autoantibodies in both groups, but further studies including multiple Fc-related effector function assays and antigen-specific autoantibody glycan profiling will be necessary in SjS to address this complex question.

To functionally explore the important role for autoantibody/FcγR engagement suggested by multiplex and IgG glycosylation data in SjS, we designed a Ro60-specific ADCP assay using the THP-1 monocytic cell line (Ackerman et al, 2011). As expected, anti-Ro60 seropositive SjS patients had the highest anti-Ro60 phagocytosis scores, suggesting a possible novel pathogenic role for these autoantibodies which could contribute to disease via inflammatory cytokine production by phagocytes after Ro60 internalisation. Ro60 is an ubiquitous and mainly intracellular ribonucleoprotein, however it can be exposed to the immune system through cellular death and tissue damage (Burbelo et al, 2021). If confirmed by further studies, including other SjS-related autoantibodies and ADCP assays using primary cells, this finding could strengthen the rationale of targeting autoantibodies-producing cells and/or autoantibodies in clinical trials (Chalayer et al, 2022).

An important limitation of this proof-of-concept study is the relatively limited number of individuals in each group, especially for the subgroup analysis in seronegative patients, preventing us to formally conclude about a statistically significant improvement of our approach compared to the currently used diagnostic methods. It is important to note that some degree of overfitting was identified in the 17-feature seronegative model, which was derived from a limited number of seronegative SjS patients (*n* = 18) and healthy controls (*n* = 40), however all 3 models’ results seemed consistent. Moreover, at the biological level, the association of FcγR engagement and related effector functions were consistently identified for classical and non-classical autoantibodies in SjS, and via different technical approaches including total IgG glycosylation profiling and an ADCP functional assay. Nevertheless, additional studies in bigger (especially including more seronegative SjS patients) independent cohorts, including a validation one, are needed to deepen and confirm our findings, better understand their underlying mechanisms, and determine their possible clinical applications.

Overall, our study provides further insight into the autoantibody responses in Sjögren’s syndrome, especially identifying a novel association and presumed pivotal role for classical, but also non-classical, autoantibody-specific Fc/FcγR-related effector functions, unveiling novel areas of research for known and lesser-known autoantibodies, with possible practical beneficial consequences for patients’ diagnosis and treatment.

## Methods

**Table:** Reagents and tools table

Reagent/resource	Reference or source	Identifier or catalog number
**Experimental models**		
THP-1 monocytes	ATCC	TIB-202
**Recombinant DNA**		
**Antibodies**		
Mouse anti-Human IgG Fc-PE	Southern Biotech	9042-09
Mouse anti-Human IgG1 Fc-PE	Southern Biotech	9054-09
Mouse anti-Human IgG2 Fc-PE	Southern Biotech	9070-09
Mouse anti-Human IgG3 Hinge-PE	Southern Biotech	9210-09
Mouse anti-Human IgG4 Fc-PE	Southern Biotech	9200-09
Mouse anti-Human IgA1-PE	Southern Biotech	9130-09
Mouse anti-Human IgA2-PE	Southern Biotech	9140-09
PE-conjugated mouse anti-human IgM	Southern Biotech	9020-09
**Oligonucleotides and other sequence-based reagents**		
**Chemicals, enzymes and other reagents**		
FcγRIIa-H131, FcγRIIa-R131, FcγRIIb, FcγRIIIa-V158, and FcγRIIIa-F158 Fc receptor dimers	Courtesy of Bruce Wines and Mark Hogarth, Immune Therapies Group, Brunet Institute; Wines et al J Immunol 2016	
MES Buffer, 0.5 M, pH 5	Thermo Fisher Scientific	J62081-AE
Ro60/SSA, Ro52/SSA, La/SSB proteins	AROTEC Diagnostics	ATR02, ATR05, ALA01
Interferon Gamma Inducible Protein 16 Recombinant Protein (Ifi16)	MyBioSource.com	MBS2030339
Muscarinic acetylcholine receptor M3 Recombinant Protein (Chmr3)	MyBioSource.com	MBS9422241
Alpha-Fodrin Recombinant Protein (Sptan1)	MyBioSource.com	MBS2010596
Carbonic Anhydrase VI Recombinant Protein (Ca6)	MyBioSource.com	MBS2105437
Alpha-enolase Active Protein (Eno1)	MyBioSource.com	MBS203689
Stathmin-4 Recombinant Protein (Stmn4)	MyBioSource.com	MBS1324761
16.5 kDa submandibular gland glycoprotein Recombinant Protein (Spt1)	MyBioSource.com	MBS1111963
Sjogren syndrome nuclear autoantigen 1 Recombinant Protein (Ssna1)	MyBioSource.com	MBS1118394
Mdm2 p53 Binding Protein Homolog Recombinant Protein (Mdm2)	MyBioSource.com	MBS2030022
Cofilin-1 Recombinant Protein (Cfl1)	MyBioSource.com	MBS286542
Rho GDP Dissociation Inhibitor Beta Recombinant Protein (Rgi2)	MyBioSource.com	MBS2030776
Tetanus toxoid	Sigma-Aldrich	T3194
Influenza A H1N1 (A/California/07/2009) Hemagglutinin / HA Protein	Sino Biological	11085-V08H
MagPlex-C Microspheres, Region XX, 1 mL	Luminex	MC100XX-01
Sulfo-N-hydroxysulfosuccinimide, format No-Weigh	Thermo Fisher Scientific	A39269
1-Ethyl-3-(3-dimethylaminopropyl) carbodiimide	Thermo Fisher Scientific	A35391
Melon Gel IgG Spin Purification Kit	Thermo Fisher Scientific	45206
Amicon Ultra-0.5 Centrifugal Filter Devices	Merck	UFC500324, UFC501024, UFC503024, UFC505024, UFC510024
Human IgG ELISA development kit	Mabtech	3850-1AD-6
EZ-Link Sulfo-NHS-LC biotinylation kit	Thermo Scientific	21435
1 μm fluorescent NeutrAvidin Fluospheres beads	Invitrogen	F8776
Streptavidin-R-Phycoerythrin conjugate (SAPE)	Invitrogen	S866
C1q protein	MP Biomedicals	80295-33-6
**Software**		
Prism 10	GraphPad	
LabChip GX Reviewer	PerkinElmer	
MATLAB	MathWorks	
Eigenvector PLS Toolbox	Eigenvector Research	
FlowJo	BD	Version 10.7.1
xPONENT Software Solutions	Luminex	
**Other**		
FlexMap 3D Instrument System	Luminex	
LabChip GXII Touch instrument	PerkinElmer	
BD LSR Fortessa with HTS	Becton Dickinson	
384-well clear-bottom plate	Greiner Bio-One	781076

### Methods and protocols

#### Study participants

Samples were collected as part of the I GET DRY (NCT03003572) multicentric cohort, whose patients were recruited in the Auvergne-Rhône-Alpes region in France between March 2018 and December 2019, after Ethics Committee (Comité de Protection des Personnes Est-III) approval (IDRCB 2016-A01851-50). The cohort (Table 2) contains healthy blood donors (all provided written consent; *n* = 40), non-SjS sicca syndrome patients (i.e., patients presenting with sicca syndrome but not fulfilling any classification criteria for SjS; *n* = 16), and SjS patients (i.e., patients fulfilling the 2002 American-European Consensus Group [AECG] (Vitali et al, 2002) and/or the 2016 American College of Rheumatology [ACR]/European League Against Rheumatism [EULAR] classification criteria; *n* = 58) (Shiboski et al, 2017). Among non-SjS sicca syndrome patients and SjS syndrome patients, EULAR Sjögren’s Syndrome Disease Activity Index (ESSDAI), EULAR Sjögren’s Syndrome Patient Reported Index (ESSPRI) and focus score (i.e., number of mononuclear cell infiltrates containing at least 50 inflammatory cells in a 4 mm^2^ minor salivary glandular section) were available (Seror et al, 2016). Recommended biological tests for SjS workup were available for all non-SjS sicca syndrome and SjS patients, including anti-Ro/SSA and anti-La/SSB IgG. Patients were classified as seropositive (*n* = 40) or seronegative (*n* = 18) using the ELISA-like Phadia™ 250 system (Thermo Fisher Scientific), based on positivity (according to the threshold provided by the manufacturer) for ≥1 autoantibody among the classical Ro/SSA or La/SSB antigens. All experiments involving human subjects conformed to the principles set out in the WMA Declaration of Helsinki and the Department of Health and Human Services Belmont Report.

#### Customized Sjögren’s syndrome bead-based multiplex assay (Selva et al, 2021a; Tosif et al, 2020; Zhang et al, 2022)

A customized multiplex assay was designed including an array of relevant autoantigens (Vílchez-Oya et al, 2022) (Table 1): the classical autoantigens (Ro60/SSA, Ro52/SSA, La/SSB; AROTEC Diagnostics), as well as non-classical “investigative” autoantigens previously identified to have some association with SjS in the literature (carbonic anhydrase 6 [Ca6], cofilin 1 [Cfl1], cholinergic muscarinic receptor 3 [Chmr3], enolase 1 [Eno1], interferon gamma-inducible protein 16 [Ifi16], La, mouse double minute 2 homolog [Mdm2], rho GDP-dissociation inhibitor 2 [Rgi2], salivary gland protein 1 [Spt1], alpha fodrin [Sptan1], Sjogren’s syndrome nuclear autoantigen 1 [Ssna1], stathmin 4 [Stmn4]; MyBioSource Inc., USA). Tetanus toxoid (Sigma) and influenza hemagglutinin (H1Cal2009; Sino Biological) were also added to the assay as positive controls. Magnetic carboxylated beads (Luminex) were covalently coupled to antigens using a two-step carbodiimide reaction, as per manufacturer’s instructions (Selva et al, 2021a; Lopez et al, 2021). Briefly, beads were washed and activated in 100 mM monobasic sodium phosphate, pH 6.2, followed by the addition of Sulfo-N-hydroxysulfosuccinimide and 1-Ethyl-3-(3-dimethylaminopropyl) carbodiimide (Thermo Fisher Scientific). After incubation at room temperature for 30 min, the activated microspheres were washed three times and resuspended in (Thermo Fisher Scientific). The respective antigens were added to the activated beads and the mixture was incubated at room temperature for 3 h on a rotator in the dark. Subsequently, the beads were washed with phosphate buffered saline (PBS) and blocked with blocking buffer (PBS, 0.1% BSA, 0.02% Tween-20, 0.05% sodium azide, pH 7) for 30 min. Finally, beads were washed in PBS 0.05% sodium azide and resuspended as one million beads per 100 µl. The isotypes and subclasses of SjS-specific autoantibodies present in the collected serum were assessed using a multiplex assay as described (Fig. 1) (Selva et al, 2021a). Using a black, clear-bottom 384-well plate (Greiner Bio-One), 20 µl of working bead mixture containing 1000 beads per bead region and 20 µl of diluted serum were added per well. From validation experiments in which cross-reactive antibodies present in healthy individuals were titrated, an optimal concentration of 1:800 working dilution of serum was selected for downstream assays. The plate was covered and incubated overnight at 4 °C on a shaker and was then washed with PBS containing 0.05% Tween-20.

SjS-specific autoantibodies were detected using phycoerythrin (PE)-conjugated mouse anti-human pan-IgG, IgG1-4, IgA1-2, IgM (Southern Biotech), at 1.3 µg/ml, 25 µl per well. After incubation at room temperature for 2 h on a shaker, the plate was washed before the beads were resuspended in 50 µl of sheath fluid. For the detection of FcγR, biotinylated soluble recombinant FcγR dimers (higher affinity polymorphism FcγRIIa-H131, lower affinity polymorphism FcγRIIa-R131, FcγRIIb, higher affinity polymorphism FcγRIIIa-V158, lower affinity polymorphism FcγRIIIa-F158; provided by Bruce Wines and Mark Hogarth, Burnet Institute), previously described to correlate with a range of Fc functions (McLean et al, 2017; Lee et al, 2021), were added at 1.3 µg/ml, 25 µl per well. After incubation at room temperature for 2 h on a shaker, the plate was washed, and streptavidin-R-Phycoerythrin conjugate (SAPE, Invitrogen) was added at 1 µg/ml, 25 µl per well. The plate was then incubated at room temperature for 2 h on a shaker before being washed as mentioned above. For the detection of C1q, C1q protein (MP Biomedicals) was first biotinylated (Thermo Fisher Scientific), washed and resuspended in PBS and tertramerized with SAPE. Tetrameric C1q-PE were added at 1 µg/ml, 25 µl per well, incubated at room temperature for 2 h on a shaker and then washed as mentioned above. After the last washing step, the plates were incubated at room temperature for 10 min on a shaker before being read by the FlexMap 3D (Luminex). The binding of the PE-coupled detectors was measured to calculate the median fluorescence intensity (MFI) for each feature among the 196 autoantibodies-related measured features. Background subtraction was conducted by removing background of blank (buffer and beads only) wells. Threshold for positivity for each feature was determined as the mean MFI + 2 standard deviations in the healthy donors (*n* = 40). Assays were repeated in duplicate.

#### Total IgG antibody purification

Total IgG were purified from the collected serum via Melon Gel chromatography according to manufacturer’s protocol as previously described (Haycroft et al, 2023) (Melon Gel IgG Purification Kit, Thermo Fisher Scientific). Total IgG antibody samples were centrifugated through 100 kDa Amicon Ultra filters (Merck) at 14,000 × *g* for 10 min to remove excess albumin proteins and buffer exchanged into PBS. The IgG concentration and purity were quantitated using a human IgG ELISA development kit (Mabtech). Total IgG antibody samples were diluted in PBS to adjust concentration to 0.25 mg/mL. The samples were stored at −20 °C until further use.

#### Total IgG N-linked glycosylation profiling

*N*-linked glycosylation profiles of total purified IgG antibodies were measured on the LabChip^®^ GXII Touch instrument (PerkinElmer) according to the ProfilerPro glycan profiling LabChip^®^ GXII Touch protocol as previously described (Haycroft et al, 2023). Microchip capillary electrophoresis-laser-induced fluorescence (CE-LIF) analysis of digested and labelled *N*-linked glycans was performed. The relative prevalence of several glycosylation profiles of total IgG antibodies were analyzed using the LabChip GX Reviewer (PerkinElmer) software. Peaks were assigned based on migration of known standards and glycan digests (Mahan et al, 2015). Peak area and therefore the relative prevalence of each glycan pattern was calculated, and results were presented as mean ± standard error of the mean.

#### Bead-based THP-1 antibody-dependent phagocytosis assay

To examine ADCP mediated by SjS’ autoantibodies, a previously described bead-based assay (Ackerman et al, 2011) was adapted to the Ro60/SSA antigen. Ro60/SSA was biotinylated using EZ-Link Sulfo-NHS-LC biotinylation kit (Thermo Scientific) with 20 mmol excess according to the manufacturer’s instructions and buffer exchanged using 30 kDa Ultra filters (Merck) to remove free biotin. The binding sites of 1 μm fluorescent NeutrAvidin Fluospheres beads (Invitrogen) were coated with biotinylated Ro60/SSA at a 1:1 ratio overnight at 4 °C. Ro60/SSA-conjugated beads were washed four times with 1% BSA/PBS to remove excess antigen and incubated with serum (1:1600 working dilution) for 2 h at 37 °C in a 96-well U-bottom plate. THP-1 monocytes (100,000/well) were then added to opsonized beads and incubated for 22 h under cell culture conditions. Cells were fixed with 2% formaldehyde and acquired on a BD LSR Fortessa with a HTS (Becton Dickinson). The data was analyzed using FlowJo 10.7.1, and a phagocytosis score was calculated as previously described (Darrah et al, 2007) using the formula: (%bead-positive cells × median fluorescent intensity). Results were presented as mean ± standard error of the mean. To account for non-specific uptake of Ro60/SSA-conjugated beads, the phagocytosis scores for each serum sample were subtracted with that of the “no serum” control.

For this assay, anti-Ro60 IgG seropositive (*n* = 33) and anti-Ro60 IgG seronegative (*n* = 25) SjS patients were considered, along with non-SjS sicca syndrome patients and healthy controls.

#### Statistical analysis

The SjS versus healthy controls Volcano plot was conducted using Prism 10. Statistical significance determined using the Holm-Sidak method, with α  =  0.05 adjusted for 196 tests (autoantibodies-related features). Each feature was analysed individually, without assuming a consistent standard deviation. The overall multiplex dataset was analysed for normal distribution using the Shapiro–Wilk test by Prism 10. The data were further analyzed using one-way ANOVA (Kruskal–Wallis one-way analysis with Dunn’s multiple comparison) using Prism 10.

#### Principal component analysis

PCA, performed using Prism 10, is an unsupervised technique that was used to visualize the variance in the samples based on all of the measured features. Every feature is assigned a loading, the linear combinations of these loadings create a principal component (PC). Loadings and PCs are calculated to describe the maximum amount of variance in the data. Each sample is then scored and plotted using their individual response measurements expressed through the PCs. The percent of variance described by each PC is a measure of the amount of variance in antibody response explained by that respective PC. Separation of groups on the scores plot indicates unsupervised separation of groups based on all features. Vectors (one per autoantibodies-related feature) on the loadings plot represent the correlation between the variables and the principal components.

#### Feature selection using elastic Net/PLSDA

To determine the minimal set of features (signatures) needed to distinguish cohorts (healthy, non-SjS sicca syndrome patients, seronegative SjS, seropositive SjS) a three-step process was used as described before (Selva et al, 2021b; Zhang et al, 2022), based on Gunn et al (Gunn et al, 2018). First, the data were randomly sampled without replacement to generate 2000 subsets. The resampled subsets spanned 80% of the original sample size or sampled all classes at the size of the smallest class for categorical outcomes, which corrected for any potential effects of class size imbalances during regularization. Elastic-Net regularization was then applied to each of the 2000 resampled subsets to reduce and select features most associated with the outcome variables. The Elastic-Net hyperparameter, α, was set to have equal weights between the L1 norm and L2 norm associated with the penalty function for least absolute shrinkage and selection (LASSO) and ridge regression, respectively. By using both penalties, Elastic-Net provides sparsity and promotes group selection. The frequency at which each feature was selected across the 2000 iterations was used to determine the signatures by using a sequential step-forward algorithm that iteratively added a single feature into the PLSDA model starting with the feature that had the highest frequency of selection, to the lowest frequency of selection. Model prediction performance was assessed at each step and evaluated by 10-fold cross-validation classification error. The model with the lowest classification error within a 0.01 difference between the minimum classification error was selected as the minimum signature. If multiple models fell within this range, the one with the least number of features was selected.

To explore the diagnostic ability of binary classifiers, Receiver operating characteristic (ROC) curves for PLSDA antibody classification signatures were generated.

#### Partial least squares discriminant analysis

PLSDA, performed in Eigenvectors PLS toolbox in Matlab, was used in conjunction with Elastic-Net, described above, to identify and visualize signatures that distinguish categorical outcomes. This supervised method assigns a loading to each feature within a given signature and identifies the linear combination of loadings (a latent variable, LV) that best separates the categorical groups. A feature with a high loading magnitude indicates greater importance for separating the groups from one another. Each sample was then scored and plotted using their individual response measurements expressed through the LVs. The scores and loadings can then be cross-referenced to determine which features are loaded in association with which categorical groups (positively loaded features are higher in positively scoring groups, etc.). All models were orthogonalized to enable clear visualization of the results.

#### Evaluation of the models’ performance, calibration and risk for overfitting

All models go through 10-fold cross-validation, where iteratively 10% of the data is left out as the test set, and the rest is used to train the model. Model performance is measured through calibration error (average error in the training set) as well as cross-validation error (average error in the test set), with values near 0 being best. Moreover, models’ probabilistic outputs were evaluated by plotting the observed status of each individual against their predicted probability of being classified as non-SjS sicca syndrome or SjS patients. Finally, regarding the risk for overfitting, a permutation analysis was performed, by randomly and repeatedly shuffling the class labels and rerunning the total pipeline analysis (1000 iterations per model), to assess whether the identified features were selected beyond chance levels.

## Supplementary information

Peer Review File

Source data Fig. 2

Source data Fig. 3

Source data Fig. 4

Source data Fig. 5

Source data Fig. 6

Source data Fig. 7

Expanded View Figures

## Data Availability

This study includes no data deposited in external repositories. The source data of this paper are collected in the following database record: biostudies:S-SCDT-10_1038-S44321-026-00458-w.
