# Supplementary material for: Systems serology identifies FcR-related autoantibody signatures and functions for Sjögren’s syndrome
Source: EMBO Mol Med. 2026 Jun 17;18(7):2808–37. doi: 10.1038/s44321-026-00458-w (PMC13365422; doi:10.1038/s44321-026-00458-w)
Supplement: Supplementary file 8 — Expanded View Figures [file 44321_2026_458_MOESM8_ESM.pdf]

Expanded View Figures

Figure EV1. Anti-Ro52 and anti-La specific responses.

Univariate analysis across groups (40 healthy controls in blue, 16 non-SjS sicca syndrome in green, and 58 Sjögren's syndrome [SjS] patients in orange) were performed with multiplex for (**A**) anti-Ro52 and (**B**) anti-La specific responses. Anti-Ro52 features were higher in SjS patients than in non-SjS sicca syndrome patients and healthy controls: anti-Ro52 IgG ($P = 0.0001$ and $P < 0.0001$ respectively), FcGR2a-R131 Ro52 ($P = 0.0004$ and $P < 0.0001$ respectively), FcGR2a-H131 Ro52 ($p = 0.0007$ and $p < 0.0001$ respectively), FcGR3a-V158 Ro52 ($P = 0.0018$ and $P < 0.0001$ respectively), FcGR3a-F158 Ro52 ($P = 0.0099$ and $P = 0.0003$ respectively) and FcGR2b Ro52 ($P = 0.0018$ and $P < 0.0001$ respectively) engagements. Anti-La features were higher in SjS patients than in non-SjS sicca syndrome patients and healthy controls: anti-La IgG ($P = 0.0020$ and $P < 0.0001$, respectively), FcGR2a-R131 La ($P = 0.0077$ and $P = 0.0002$, respectively), FcGR2a-H131 La ($P = 0.0133$ and $P = 0.0004$, respectively), FcGR3a-V158 La ($P = 0.0146$ and $P = 0.0004$, respectively), FcGR3a-F158 La ($P = 0.0293$ and $P = 0.0011$, respectively) and FcGR2b La ($P = 0.0164$ and $P = 0.0006$, respectively) engagements. Multiplex assays were repeated in duplicates, statistical comparisons were performed using one-way ANOVA (Kruskal–Wallis with Dunn's multiple comparisons) and significant differences denoted with asterisks (ns = non-significant; *$P < 0.05$; **$P < 0.01$; ***$P < 0.001$; ****$P < 0.0001$).

© The Author(s)

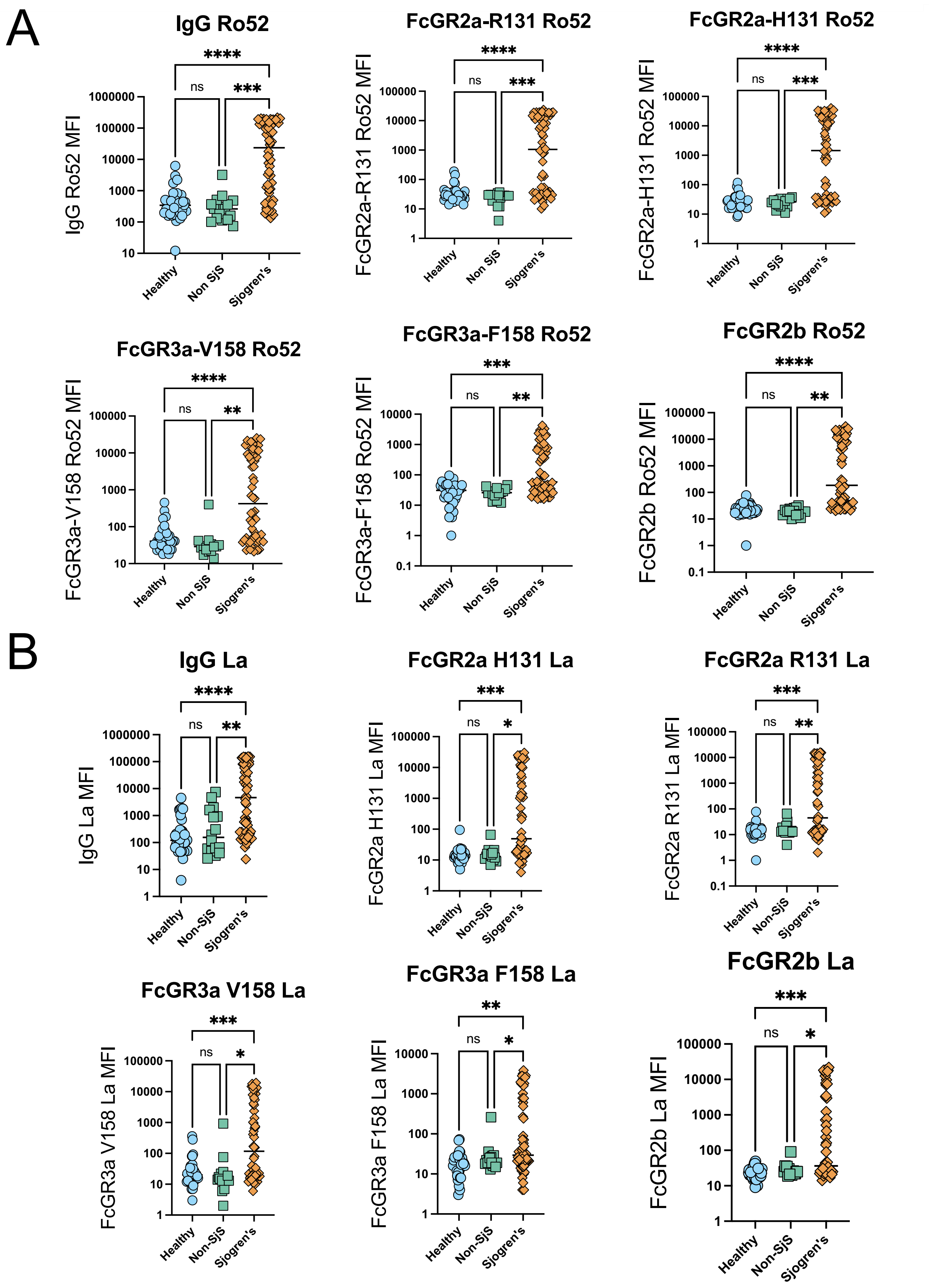
A
IgG Ro52
FcGR2a-R131 Ro52
FcGR2a-H131 Ro52
FcGR3a-V158 Ro52
FcGR3a-F158 Ro52
FcGR2b Ro52
IgG Ro52 MFI
FcGR2a-R131 Ro52 MFI
FcGR2a-H131 Ro52 MFI
FcGR3a-V158 Ro52 MFI
FcGR3a-F158 Ro52 MFI
FcGR2b Ro52 MFI
Healthy
Non SjS
Sjogren's
ns
B
IgG La
FcGR2a H131 La
FcGR2a R131 La
FcGR3a V158 La
FcGR3a F158 La
FcGR2b La
IgG La MFI
FcGR2a H131 La MFI
FcGR2a R131 La MFI
FcGR3a V158 La MFI
FcGR3a F158 La MFI
FcGR2b La MFI
Healthy
Non-SjS
Sjogren's

 © The Author(s)

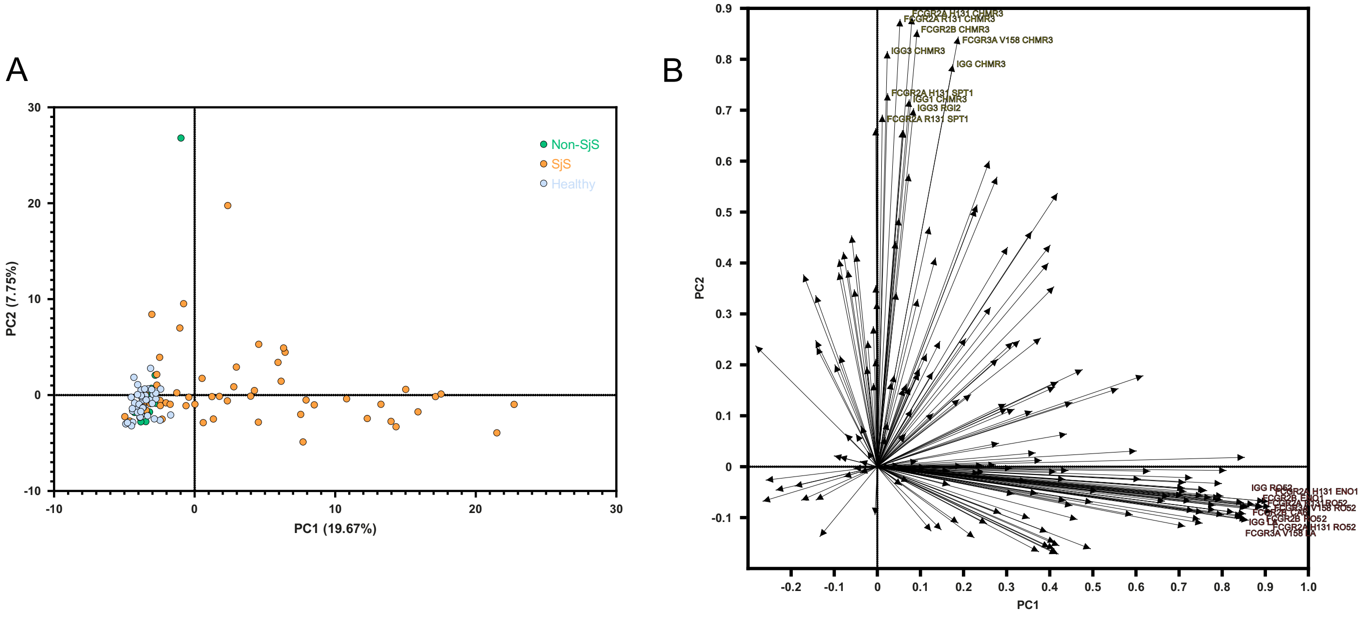

Figure EV2. Principal component analysis using the 196 autoantibodies-related features.

(**A**) The projection of samples from SjS patients ($n = 58$; orange), healthy controls ($n = 40$; blue), and non-SjS sicca syndrome patients ($n = 16$; green) are presented, and variance explained by each principal component (PC) in parenthesis. SjS patients are correctly separated from healthy controls and non-SjS sicca syndrome patients, mainly across LV1 (top 10 loadings including anti-La FcγRIIa H131, R131, IgG, FcγRIIIa V158, and FcγRIIb; anti-Eno1 FcγRIIa H131 and FcγRIIb; anti- FcγRIIb Ca6; anti-La FcγRIIIa V158 and IgG) and a subset of SjS patients is distinguishable across LV2 (top 10 loadings including anti-Chmr3 FcγRIIa H131, R131, FcγRIIb, FcγRIIIa V158, IgG3, IgG1 and IgG; anti-Rgi2 IgG3; anti-Spt1 FcγRIIa R131, and IgG3). (**B**) The corresponding loadings plot is shown, with the top 10 contributors to PC1 labelled in red, and top 10 contributors to PC2 in yellow. Multiplex assays were repeated in duplicates

© The Author(s)

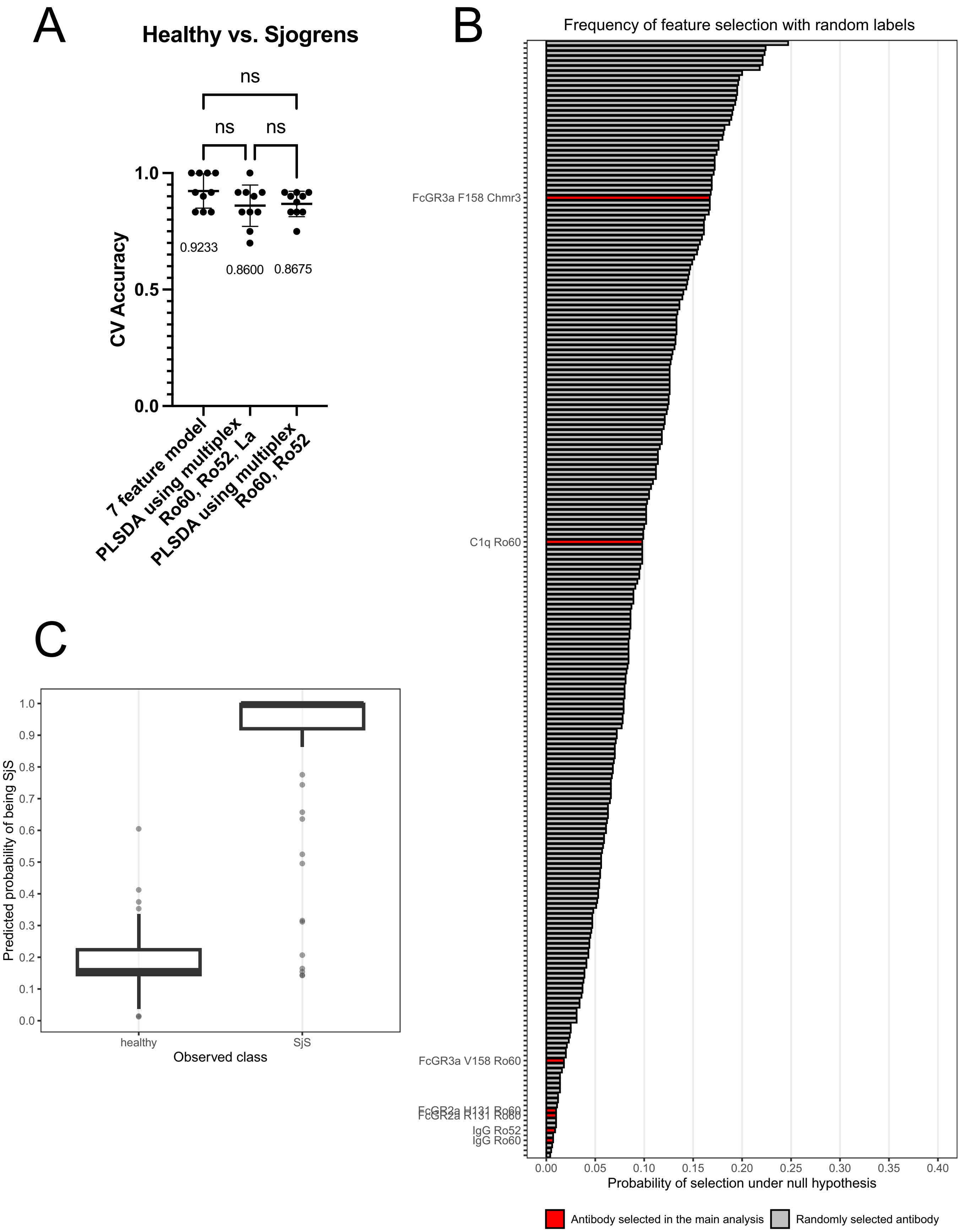
A
Healthy vs. Sjogrens
ns
ns
ns
1.0
0.5
0.0
CV Accuracy
0.9233
0.8600
0.8675
7 feature model
PLSDA using multiplex Ro60, Ro52, La
PLSDA using multiplex Ro60, Ro52
B
Frequency of feature selection with random labels
FcGR3a F158 Chmr3
C1q Ro60
FcGR3a V158 Ro60
FcGR2a H131 Ro60
FcGR2a R131 Ro60
IgG Ro52
IgG Ro60
0.00 0.05 0.10 0.15 0.20 0.25 0.30 0.35 0.40
Probability of selection under null hypothesis
Antibody selected in the main analysis
Randomly selected antibody
C
Predicted probability of being SjS
1.0 0.9 0.8 0.7 0.6 0.5 0.4 0.3 0.2 0.1 0.0
healthy
SjS
Observed class

Figure EV3. Performances of the 7-feature signature in Sjögren's syndrome.

(**A**) Comparison of cross-validation errors for the Elastic-Net-selected 7-feature antibody signature versus a 3-feature antibody signature (limited to the classical multiplex-based anti-Ro60, anti-La and anti-La IgG responses) versus a signature including only multiplex-based anti-Ro60 and anti-La IgG responses for Sjögren's syndrome (SjS; $n = 58$) diagnosis compared to healthy controls ($n = 40$). Results are presented as means ± standard deviations. Statistical comparisons were performed using one-way ANOVA (Kruskal–Wallis with Dunn's multiple comparisons), and significant differences denoted with asterisks (ns = non-significant; *$P < 0.05$; **$P < 0.01$; ***$P < 0.001$; ****$P < 0.0001$). (**B**) Representation of the selection probabilities for each autoantibody-related feature under the null hypothesis (i.e., after permutation of class labels), to evaluate the performance of the 7-feature model. (**C**) Model calibration analysis evaluating concordance between predicted probabilities of being a SjS patient, and observed frequencies for healthy controls and SjS patients. Multiplex assays were repeated in duplicates.

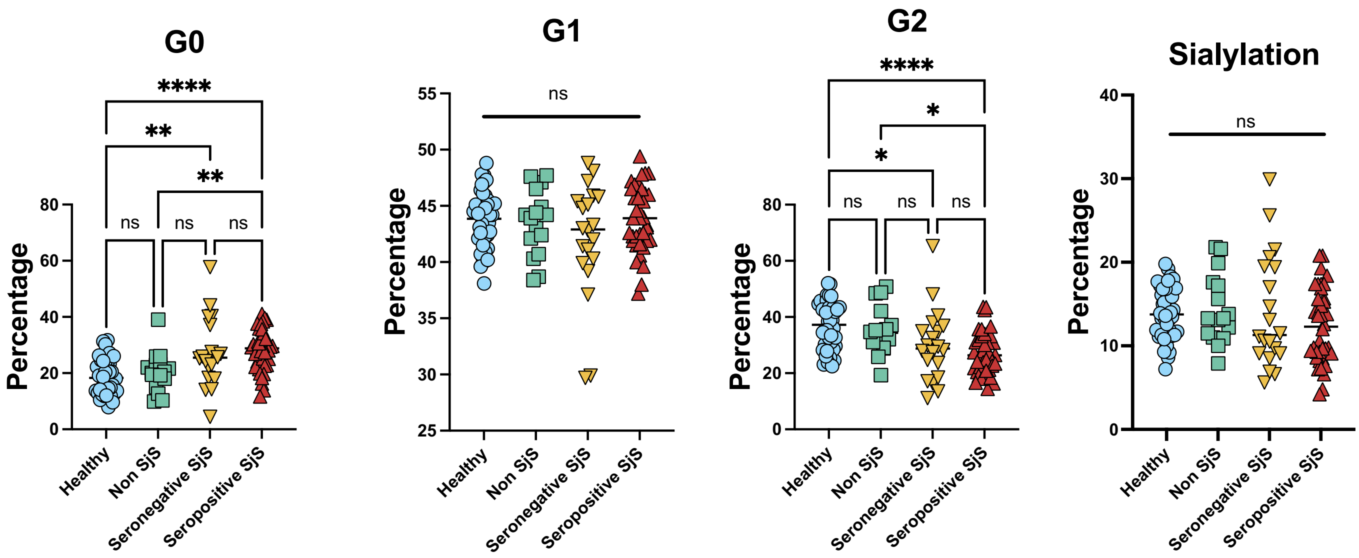

Figure EV4. Total IgG galactosylation and sialylation profiling.

Comparisons of the relative abundance of the different galactosylation profiles (G0 = no galactose residue; G1 = 1 galactose residue; G2 = 2 galactose residues) and sialylation of total IgG were performed between seropositive (i.e., positive for anti-Ro/SSA and/or anti-La/SSB according to clinically-used ELISA-like assay results) Sjögren's syndrome (SjS) patients ($n = 40$; red), seronegative SjS patients ($n = 18$; yellow), non-SjS sicca syndrome patients ($n = 16$; green) and healthy controls ($n = 40$; blue). Total IgG G0 residue relative abundance was higher in seropositive SjS patients compared to non-SjS sicca syndrome patients ($P = 0.0075$) and healthy controls ($P < 0.0001$). Total IgG G0 residue relative abundance was also higher in seronegative SjS patients compared to healthy controls ($P = 0.0012$). Total IgG G2 residue relative abundance was lower in seropositive SjS patients compared to non-SjS sicca syndrome patients ($P = 0.0172$) and healthy controls ($P < 0.0001$). Total IgG G2 residue relative abundance was also lower in seronegative SjS patients compared to healthy controls ($P = 0.0307$). Glycosylation profiling were repeated in duplicates, statistical comparisons were performed using one-way ANOVA (Kruskal–Wallis with Dunn's multiple comparisons) and significant differences denoted with asterisks (ns = non-significant; *$P < 0.05$; **$P < 0.01$; ***$P < 0.001$; ****$P < 0.0001$).

 © The Author(s)

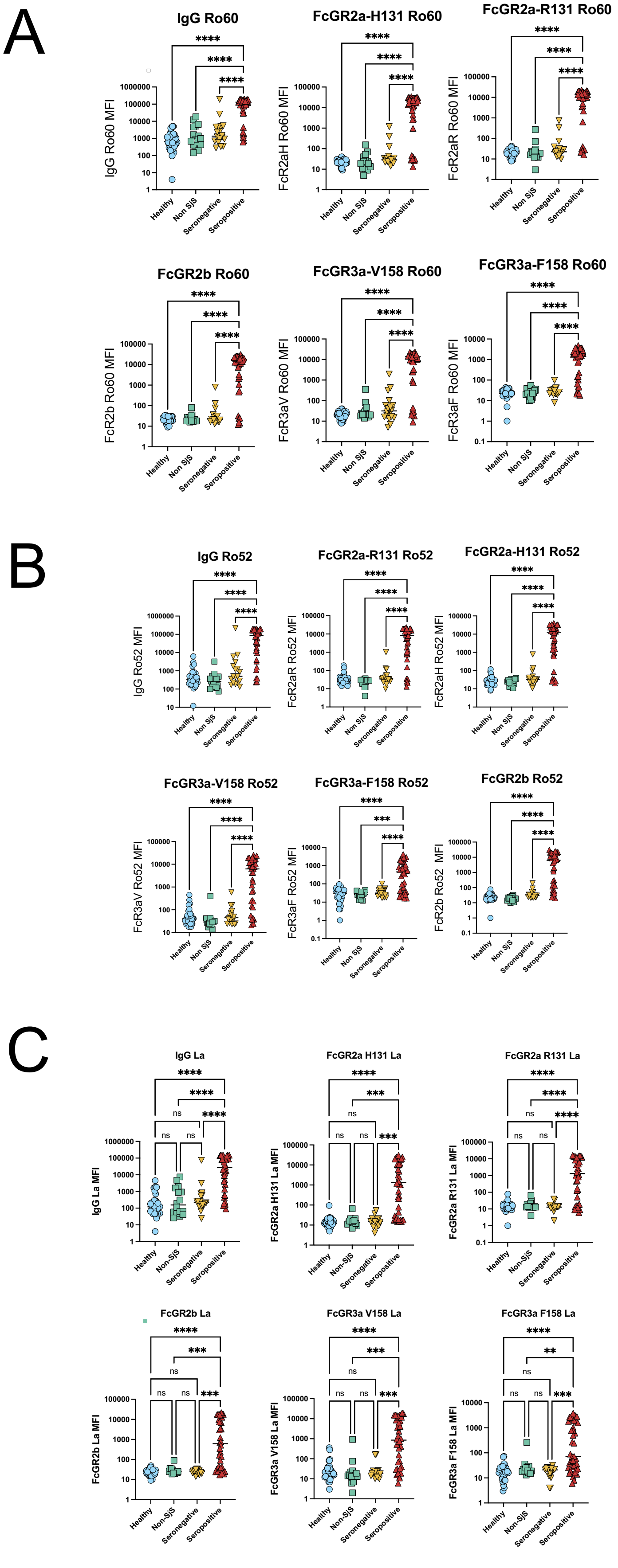
A
IgG Ro60
FcGR2a-H131 Ro60
FcGR2a-R131 Ro60
IgG Ro60 MFI
FcR2aH Ro60 MFI
FcR2aR Ro60 MFI
FcGR2b Ro60
FcGR3a-V158 Ro60
FcGR3a-F158 Ro60
FcR2b Ro60 MFI
FcR3aV Ro60 MFI
FcR3aF Ro60 MFI
Healthy
Non SjS
Seronegative
Seropositive
B
IgG Ro52
FcGR2a-R131 Ro52
FcGR2a-H131 Ro52
IgG Ro52 MFI
FcR2aR Ro52 MFI
FcR2aH Ro52 MFI
FcGR3a-V158 Ro52
FcGR3a-F158 Ro52
FcGR2b Ro52
FcR3aV Ro52 MFI
FcR3aF Ro52 MFI
FcR2b Ro52 MFI
C
IgG La
FcGR2a H131 La
FcGR2a R131 La
IgG La MFI
FcGR2a H131 La MFI
FcGR2a R131 La MFI
FcGR2b La
FcGR3a V158 La
FcGR3a F158 La
FcGR2b La MFI
FcGR3a V158 La MFI
FcGR3a F158 La MFI
Non-SjS

Figure EV5. Anti-Ro60, anti-Ro52 and anti-La specific responses depending on the serostatus in Sjögren's syndrome.

Univariate analysis across seropositive (i.e., positive for anti-Ro/SSA and/or anti-La/SSB according to clinically-used ELISA-like assay results) Sjögren's syndrome (SjS) patients ($n = 40$; red), seronegative SjS patients ($n = 18$; yellow), non-SjS sicca syndrome patients ($n = 16$; green) and healthy control ($n = 40$; blue) groups were performed with multiplex for (**A**) anti-Ro60, (**B**) anti-Ro52 and (**C**) anti-La specific responses. All anti-Ro60 and anti-Ro52 features (specific IgG, and specific engagements of FcGR2a-R131, FcGR2a-H131, FcGR3a-V158, FcGR3a-F158 and FcGR2b) were higher in seropositive SjS patients than in seronegative SjS, non-SjS sicca syndrome patients and healthy controls (all $P < 0.0001$). Anti-La features were higher in seronegative SjS patients than in seronegative SjS, non-SjS sicca syndrome patients and healthy controls: anti-La IgG ($P < 0.0001$ for the 3 comparisons), FcGR2a-R131 La ($P < 0.0001$ for the 3 comparisons), FcGR2a-H131 La ($P = 0.0001$, $P = 0.0002$, and $P < 0.0001$ respectively), FcGR3a-V158 La ($P = 0.0001$, $P = 0.0002$, and $P < 0.0001$, respectively), FcGR3a-F158 La ($P = 0.0004$, $P = 0.0010$, and $P < 0.0001$, respectively) and FcGR2b La ($P = 0.0002$, $P = 0.0003$, and $P < 0.0001$, respectively) engagements. Multiplex assays were repeated in duplicates, statistical comparisons were performed using one-way ANOVA (Kruskal–Wallis with Dunn's multiple comparisons) and significant differences denoted with asterisks (ns = non-significant; *$P < 0.05$; **$P < 0.01$; ***$P < 0.001$; ****$P < 0.0001$).

 © The Author(s)

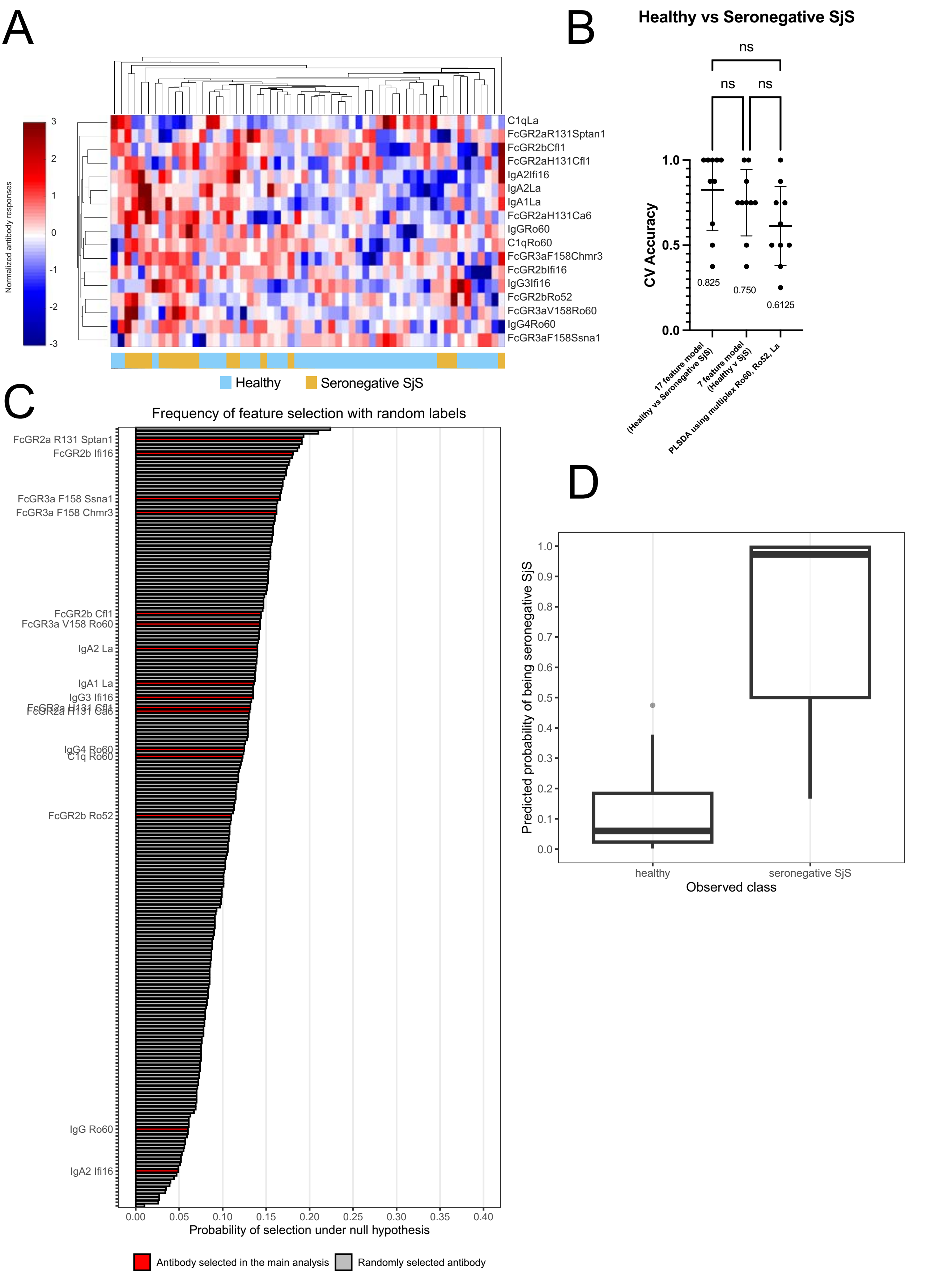
A
Normalized antibody responses
3
2
1
0
-1
-2
-3
C1qLa
FcGR2aR131Sptan1
FcGR2bCfl1
FcGR2aH131Cfl1
IgA2Ifi16
IgA2La
IgA1La
FcGR2aH131Ca6
IgGRo60
C1qRo60
FcGR3aF158Chmr3
FcGR2bIfi16
IgG3Ifi16
FcGR2bRo52
FcGR3aV158Ro60
IgG4Ro60
FcGR3aF158Ssna1
Healthy
Seronegative SjS
B
Healthy vs Seronegative SjS
ns
ns
ns
CV Accuracy
1.0
0.5
0.0
0.825
0.750
0.6125
17 feature model (Healthy vs Seronegative SjS)
7 feature model (Healthy v SjS)
PLSDA using multiplex Ro60, Ro52, La
C
Frequency of feature selection with random labels
FcGR2a R131 Sptan1
FcGR2b Ifi16
FcGR3a F158 Ssna1
FcGR3a F158 Chmr3
FcGR2b Cfl1
FcGR3a V158 Ro60
IgA2 La
IgA1 La
IgG3 Ifi16
IgG4 Ro60
C1q Ro60
FcGR2b Ro52
IgG Ro60
IgA2 Ifi16
0.00
0.05
0.10
0.15
0.20
0.25
0.30
0.35
0.40
Probability of selection under null hypothesis
Antibody selected in the main analysis
Randomly selected antibody
D
Predicted probability of being seronegative SjS
1.0
0.9
0.8
0.7
0.6
0.5
0.4
0.3
0.2
0.1
0.0
healthy
seronegative SjS
Observed class

Figure EV6. Performances of the 17-feature signature in seronegative Sjögren's syndrome.

(**A**) Hierarchical clustering was performed using the Elastic-Net-selected 17-feature signature identified by comparing seronegative (i.e., negative for anti-Ro/SSA and anti-La/SSB according to clinically-used ELISA-like assay results) Sjögren's syndrome (SjS) patients ($n = 18$; light orange) versus healthy controls ($n = 40$; blue). Data were z-scored prior to analysis. (**B**) Comparison of cross-validation errors for the Elastic-Net-selected 17-feature "seronegative SjS" antibody signature versus the 7-feature signature, versus a 3-feature antibody signature (limited to the classical multiplex-based anti-Ro60, anti-La and anti-La IgG responses) for SjS diagnosis in seronegative SjS. Results are presented as means ± standard deviations. Statistical comparisons were performed using one-way ANOVA (Kruskal–Wallis with Dunn's multiple comparisons), and significant differences denoted with asterisks (ns = non-significant; *$P < 0.05$; **$P < 0.01$; ***$P < 0.001$; ****$P < 0.0001$). (**C**) Representation of the selection probabilities for each autoantibody-related feature under the null hypothesis (i.e., after permutation of class labels), to evaluate the performance of the 17-feature model. (**D**) Model calibration analysis evaluating concordance between predicted probabilities of being a seronegative SjS patient, and observed frequencies for healthy controls and seronegative SjS patients. Multiplex assays were repeated in duplicates.

 © The Author(s)

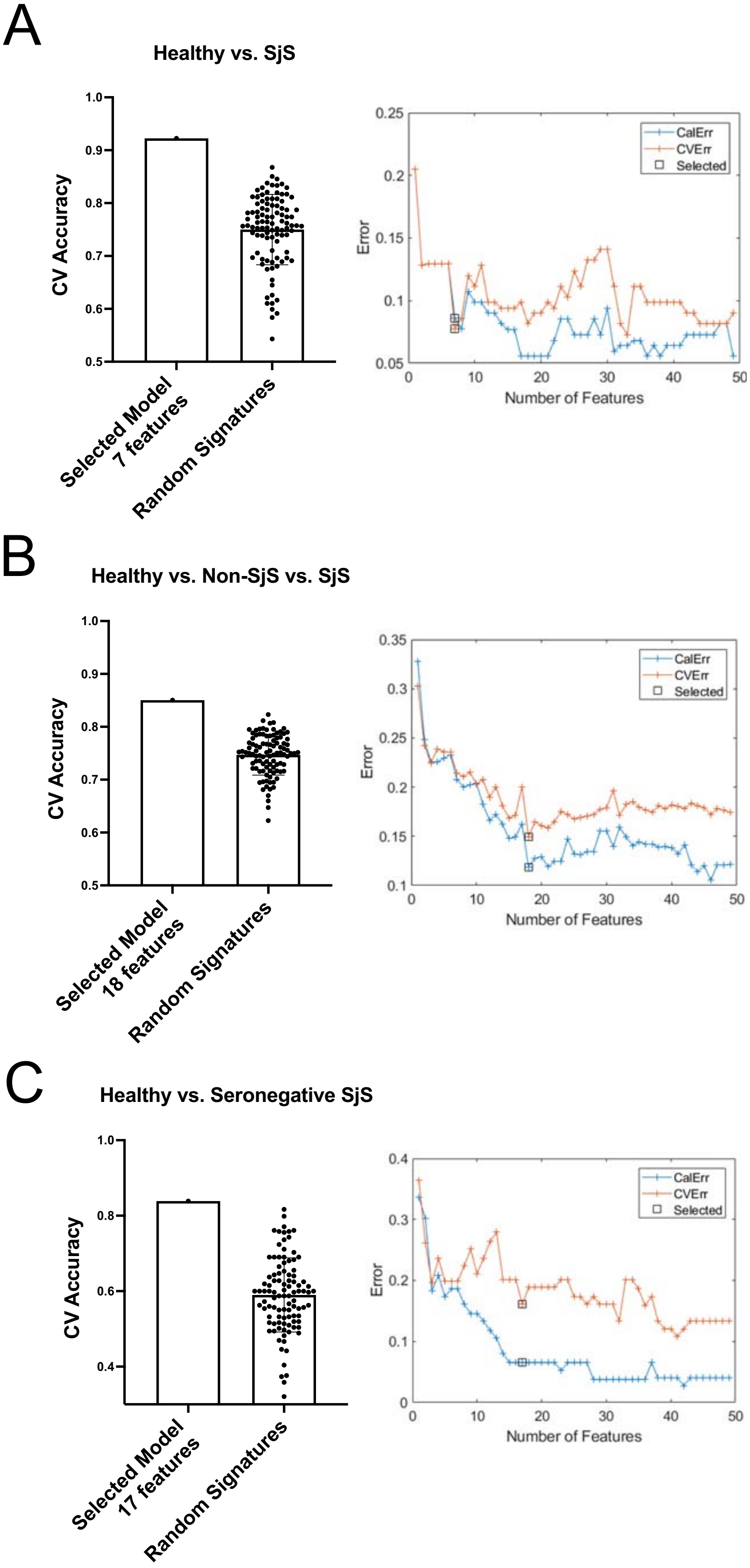
A
Healthy vs. SjS
CV Accuracy
1.0
0.9
0.8
0.7
0.6
0.5
Selected Model
7 features
Random Signatures
Error
CalErr
CVErr
Selected
Number of Features
B
Healthy vs. Non-SjS vs. SjS
CV Accuracy
Selected Model
18 features
Random Signatures
Error
CalErr
CVErr
Selected
Number of Features
C
Healthy vs. Seronegative SjS
CV Accuracy
Selected Model
17 features
Random Signatures
Error
CalErr
CVErr
Selected
Number of Features

◀ **Figure EV7. Performances of the 3 main selected signatures.**

Presentation of the calibration and cross-validation accuracy for the three identified autoantibody signatures (left) in (**A**) Sjögren's syndrome (SjS) patients and healthy donors, (**B**) SjS patients, non-SjS sicca syndrome patients and healthy donors, or (**C**) seronegative SjS patients and healthy donors to 100 randomly generated signatures with the same number of features, and the respective charts (right) showing the selected features.

 © The Author(s)
