# Supplementary material for: Systems serology identifies FcR-related autoantibody signatures and functions for Sjögren’s syndrome
Source: EMBO Mol Med. 2026 Jun 17;18(7):2808–37. doi: 10.1038/s44321-026-00458-w (PMC13365422; doi:10.1038/s44321-026-00458-w)
Supplement: Supplementary file 1 — Peer Review File [file 44321_2026_458_MOESM1_ESM.pdf]

EMBO Molecular Medicine

Systems serology identifies FcR-related autoantibody signatures and functions for Sjögren's syndrome

Martin Killian, Suzanne Shoffner-Beck, Timon Damelang, Kevin Selva, Kade Wong, Samantha Davis, Ebene Haycroft, Bruce Wines, P Hogarth, Stephen John Kent, Lucile Grange, Baptiste Gramont, Flora Schein, Héloise Munoz-Pons, Isabelle Guichard, Jean-Baptiste Gaultier, Anne-Emmanuelle Berger, Alice Haccourt, Blandine Chanut, Fabienne Jospin, Alexis Bocquet, Laurence Bouillet, Marc Ruivard, Yvan Jamilloux, Pascal Seve, Isabelle Durieu, Quitterie Reynaud, Jean-Christophe Lega, Amélie Servettaz, Hubert Marotte, Pascal Cathébras, Rémy Harlé, Edouard Ollier, Stéphane Paul, Kelly Arnold, and Amy Chung

Corresponding author(s): Martin Killian (martin.killian@chu-st-etienne.fr) , Amy Chung (awchung@unimelb.edu.au)

Review Timeline:

Submission Date:	23rd Feb 25
Editorial Decision:	26th Mar 25
Revision Received:	28th Mar 26
Editorial Decision:	17th Apr 26
Revision Received:	17th May 26
Accepted:	22nd May 26

Editor: Zeljko Durdevic

Transaction Report:

(Note: With the exception of the correction of typographical or spelling errors that could be a source of ambiguity, letters and reports are not edited. Depending on transfer agreements, referee reports obtained elsewhere may or may not be included in this compilation. Referee reports are anonymous unless the Referee chooses to sign their reports.)

26th Mar 2025

Dear Dr. Killian,

Thank you for the submission of your manuscript to EMBO Molecular Medicine. We have now received feedback from the three reviewers who agreed to evaluate your manuscript. All three referees recognize interest of the study but also raise important concerns that should be addressed in a major revision. If you would like to discuss further the points raised by the referees, I am available to do so via email or video. Let me know if you are interested in this option.

Further consideration of a revision that addresses reviewers' concerns in full will entail a second round of review. EMBO Molecular Medicine encourages a single round of revision only and therefore, acceptance or rejection of the manuscript will depend on the completeness of your responses included in the next, final version of the manuscript. For this reason, and to save you from any frustrations in the end, I would strongly advise against returning an incomplete revision.

We would welcome the submission of a revised version within three months for further consideration. Please let us know if you require longer to complete the revision.

I look forward to receiving your revised manuscript.

Yours sincerely,

Zeljko Durdevic

Zeljko Durdevic
Editor
EMBO Molecular Medicine

*****

When submitting your revised manuscript, please carefully review the instructions that follow below. We perform an initial quality control of all revised manuscripts before re-review; failure to include requested items will delay the evaluation of your revision.

We require:

1) A .docx formatted version of the manuscript text (including legends for main figures, EV figures and tables). Please make sure that the changes are highlighted to be clearly visible.

2) Individual production quality figure files as .eps, .tif, .jpg (one file per figure). For guidance, download the 'Figure Guide PDF': (https://www.embopress.org/page/journal/17574684/authorguide#figureformat).

3) A .docx formatted letter INCLUDING the reviewers' reports and your detailed point-by-point responses to their comments. As part of the EMBO Press transparent editorial process, the point-by-point response is part of the Review Process File (RPF), which will be published alongside your paper.

4) A complete author checklist, which you can download from our author guidelines (https://www.embopress.org/page/journal/17574684/authorguide#submissionofrevisions). Please insert information in the checklist that is also reflected in the manuscript. The completed author checklist will also be part of the RPF.

5) Please note that all corresponding authors are required to supply an ORCID ID for their name upon submission of a revised manuscript.

6) It is mandatory to include a 'Data Availability' section after the Materials and Methods. Before submitting your revision, primary datasets produced in this study need to be deposited in an appropriate public database, and the accession numbers and database listed under 'Data Availability'. Please remember to provide a reviewer password if the datasets are not yet public (see

https://www.embopress.org/page/journal/17574684/authorguide#dataavailability).

In case you have no data that requires deposition in a public database, please state so in this section. Note that the Data Availability Section is restricted to new primary data that are part of this study.

7) For data quantification: please specify the name of the statistical test used to generate error bars and P values, the number (n) of independent experiments (specify technical or biological replicates) underlying each data point and the test used to calculate p-values in each figure legend. The figure legends should contain a basic description of n, P and the test applied. Graphs must include a description of the bars and the error bars (s.d., s.e.m.). See also 'Figure Legend' guidelines: https://www.embopress.org/page/journal/17574684/authorguide#figureformat

8) At EMBO Press we ask authors to provide source data for the main manuscript figures. Our source data coordinator will contact you to discuss which figure panels we would need source data for and will also provide you with helpful tips on how to upload and organize the files.

9) Our journal encourages inclusion of *data citations in the reference list* to directly cite datasets that were re-used and obtained from public databases. Data citations in the article text are distinct from normal bibliographical citations and should directly link to the database records from which the data can be accessed. In the main text, data citations are formatted as follows: "Data ref: Smith et al, 2001" or "Data ref: NCBI Sequence Read Archive PRJNA342805, 2017". In the Reference list, data citations must be labeled with "[DATASET]". A data reference must provide the database name, accession number/identifiers and a resolvable link to the landing page from which the data can be accessed at the end of the reference. Further instructions are available at .

10) We replaced Supplementary Information with Expanded View (EV) Figures and Tables that are collapsible/expandable online. A maximum of 5 EV Figures can be typeset. EV Figures should be cited as 'Figure EV1, Figure EV2" etc... in the text and their respective legends should be included in the main text after the legends of regular figures.

- For the figures that you do NOT wish to display as Expanded View figures, they should be bundled together with their legends in a single PDF file called *Appendix*, which should start with a short Table of Content. Appendix figures should be referred to in the main text as: "Appendix Figure S1, Appendix Figure S2" etc.

- Additional Tables/Datasets should be labeled and referred to as Table EV1, Dataset EV1, etc. Legends have to be provided in a separate tab in case of .xls files. Alternatively, the legend can be supplied as a separate text file (README) and zipped together with the Table/Dataset file.

See detailed instructions here:

.

11) The paper explained: EMBO Molecular Medicine articles are accompanied by a summary of the articles to emphasize the major findings in the paper and their medical implications for the non-specialist reader. Please provide a draft summary of your article highlighting

- the medical issue you are addressing,

- the results obtained and

- their clinical impact.

This may be edited to ensure that readers understand the significance and context of the research. Please refer to any of our published articles for an example.

12) Author contributions: You will be asked to provide CRediT (Contributor Role Taxonomy) terms in the submission system. These replace a narrative author contribution section in the manuscript.

13) A Conflict of Interest statement should be provided in the main text.

14) Every published paper now includes a 'Synopsis' to further enhance discoverability. Synopses are displayed on the journal webpage and are freely accessible to all readers. They include a short stand first (maximum of 300 characters, including space) as well as 2-5 one-sentences bullet points that summarizes the paper. Please write the bullet points to summarize the key NEW findings. They should be designed to be complementary to the abstract - i.e. not repeat the same text. We encourage inclusion of key acronyms and quantitative information (maximum of 30 words / bullet point). Please use the passive voice. Please attach these in a separate file or send them by email, we will incorporate them accordingly.

Please also suggest a striking image or visual abstract to illustrate your article as a PNG file 550 px wide x 300-600 px high.

15) Include a Reagents and Tools Table as part of the Methods section, which can be downloaded from our author guidelines (https://www.embopress.org/page/journal/17574684/authorguide#structuredmethods)

EMBO Molecular Medicine has a "scooping protection" policy, whereby similar findings that are published by others during review or revision are not a criterion for rejection. Should you decide to submit a revised version, I do ask that you get in touch after three months if you have not completed it, to update us on the status.

***** Reviewer's comments *****

Referee #1 (Comments on Novelty/Model System for Author):

N/A

Referee #1 (Remarks for Author):

Killian et al perform systems serology analysis of patients with Sjögren's Syndrome (SjS), patients with a related syndrome, and healthy individuals. They find that the various systems serology read-outs complement analysis of a broad range of autoantigens, and that they are better able to classify the patient groups. This is an interesting study with clear conclusions and analysis. I have several major concerns that must be addressed before this study would be suitable for publication. However, should these be adequately addressed, this study is a reasonable advancement both in our understanding of SjS and in the application of systems serology to study autoimmune conditions.

Major concerns

If the authors wish to claim that the systems serology data is an important aspect here, they need to present direct evidence indicating so. For instance, how well do their classifiers compare to ones using just the antibody titer data across antigens? Given that they have this data collected, this should be a simple argument to make.

While I appreciate the supervised analysis to identify signatures of the specific patient groups, some unsupervised analysis of the data would be greatly helpful in understanding the study and cohort. The data collected is rich, and it would be helpful to understand, for instance, whether the Fc properties are similar across antigens, which autoantigens tend to appear together, etc. A simple PCA analysis of the collected data should provide much of this information.

I could not find much information on how the patients were diagnosed. Especially given that Sjögren's syndrome is diagnosed in part through an autoantibody test, I would appreciate a detailed description of these procedures. Furthermore, the authors should directly address how their reasoning is not cyclical here; that they are confident they are developing a signature of disease, and not of the diagnostic technique itself.

The manuscript seems to be missing several requisite sections, like a data availability statement and competing interests statement, but I figure these will come in revisions.

Referee #2 (Comments on Novelty/Model System for Author):

The manuscript by Killian et al. applies a Systems Serology platform, previously used in infectious disease research, to identify autoantigen-specific antibody and FcγR signatures associated with Sjögren's Syndrome (SjS), an autoimmune disorder. In addition to detecting known classical autoantibodies, the authors identify multiple novel non-classical autoantibodies associated with SjS, which may help bridge the "seronegative gap" in diagnosis and treatment. These antibodies, along with IgG, FcγR, and C1q engagement, as well as IgG N-linked glycosylation, are linked to ADCP functionality.

Overall, the manuscript is well-written, but several areas require clarification to improve the reader's understanding of the work.

1. Lines 95-114 should be condensed. The authors devote substantial space to describing general antibody functions and FcγR polymorphisms, though the latter does not appear to play a central role in the study. Some of the discussion could be moved to the introduction to describe relevant previous work done in autoantigen screening (for example, lines 379-401).
2. Reference 40 (line 162) does not seem to contain a description of the ADCP assay. Could this be an incorrect citation?

3. Reference 42 (line 167) does not appear to include the referenced "three-step process." Could this be an incorrect citation? Similarly, the same reference is cited in the Supplementary Data under "Feature selection using Elastic Net/PLSR and Elastic Net/PLSDA," which should be verified.
4. In the "Feature selection using Elastic Net/PLSR and Elastic Net/PLSDA" section of the Supplementary Data, the authors state that they generated 2000 subsets by randomly sampling without replacement. Given the sample size of the data, this seems unlikely Should this instead be "sampling with replacement"?
5. Given the observed high activation of FcγRIIIa in SjS patients, why was ADCC not performed as well?
6. The Ro/La-only PLSDA model is not described in the text. Was this model generated using Luminex data? How many features does it include?
7. Lines 214-217 require proper citation for the data being referenced.
8. Supplementary Figure 2 compares the 7-signature model to PLSDA 3 classical antigens and Ro60+Ro52 models in (A) and Ro/La PLSAD and Multiplex. However, these models are not clearly described in the manuscript and should be explained in more detail.
9. Figure legends should include the number of samples per group, as some analyses combine seronegative and seropositive SjS patients, while others separate these groups.
10. Figures 3C and 3D should be swapped to align with the text, as they are currently miscited.
11. Lines 270-279 should include proper citations to support the results described.
12. In Figure 5A, it is unclear which groups the asterisks are comparing. This should be clarified in the figure legend.
13. In Figure 5C, the label "DB" should be corrected to "Healthy."
14. Lines 298- show sample numbers lower than the reported group sizes. Was ADCP not measured for all participants? If so, this should be explicitly stated in the Methods section.

Referee #3 (Comments on Novelty/Model System for Author):

no model system is used in this study

Referee #3 (Remarks for Author):

Killian et al., report application of systems serology tools in the form of multiplex antibody profiling and machine learning to define markers of Sjogren's syndrome. They find that multiplex assay data on a rich number of measures are able to discriminate individuals with different disease status similarly well as simpler feature sets.

Major comments.
1. The modeling approaches are insufficiently well described. For example, the authors describe application of "a range of machine learning models" at line 127, but only reports results from PLSDA with elastic net. Is this the only approach applied? If so, please eliminate referring to this as a range. If not, please provide information on what additional approaches were applied, their performance, and why results only from this approach were presented.
2. It is necessary for greater rigor to be applied to the modeling. Applying the same modeling approach on permuted disease class labels in advance of feature selection and class modeling and doing do repeatedly is recommended to aid in interpretation of the quality of performance observed. There is particular concern that 17 feature models learned from 25 seronegative and 40 healthy donors is at risk of being overfit/not performing better on the actual as compared to permuted group labels. Inclusion of results from randomly selected size-matched feature sets is appreciated, but addresses a different question (how useful was feature selection) than permutation testing (how often would a model perform similarly well by random chance). Comparison with 100 randomly selected size-matched feature sets seems a difficult to interpret bar as a negative analytical control as these features were all only tested b/c of their possible diagnostic utility. If many features are different between groups, then there is a high probability of a good model based on features selected by chance. Because CV accuracy is greater than 0.7 for many of the random feature sets, would be good to bolster the methodologic approach here through permutation up front and complete model rebuilding, or to temper claims.
3. Figure 3C is mis-called out in the discussion where figure 3D is meant (and it may be considered unusual to call out figure panels in discussion). This mistake is easily corrected, however, the authors claim that the 7 feature model significantly improved SjS diagnostic performance. This reviewer could find no evidence to support this claim.
4. Do the authors have an insight into degree of confusion in misclassified samples? Is the model equally or not equally confident about samples it mis as corrected classifies?
5. The authors should better explain the relevance of total sersum IgG glycans to antigen specific antibody profiles or to disease states. There is literature on pathogenic alloimmune responses that may be of interest to discuss-especially given fucosylation differences discussed at lines 415-417. Further, the effect of galactose on FcR binding has been rigorously characterized by Dekkers/Vidarsson et al. and ...Barb et al.,. It would seem be better to summarize this highly well-controlled work rather than cop out on the basis of studies of associations in polyclonal sera where many other factors are likely to co-vary.
6. The text should make clear that different Abs are being profiled in different parts of Fig 5. The legend did a better job of this,

but this reviewer nonetheless finds it misleading to lump these panels together.
7. The manuscript would benefit from better justification of focus on one function.
8. The manuscript insufficiently describes its limitations. These include lack of a validation cohort and lack of clear (statistically significant) improvements over current diagnostic methods, among others.

Minor comments.
1. As someone unfamiliar to SjS notation, I found supplementary table 1 to be highly helpful, especially as antigens were introduced and referred to with different subsets of their names over time. It may add sufficient value to merit inclusion as a main table or to provide additional nomenclature information (e.g. SSA, SSB) in figure 1.
2. Since Trim21 is an Fc binding protein, how do the authors exclude interactions with this protein though parts of IgG other than CDRs/Fv/Fab regions?
3. Unclear what "dedicated" means in the context of its use on line 63.
4. "findings provides" needs grammatical correction on line 66.
5. "Antibody associated" on line 124 should be hyphenated.
6. At line 157 the application of IgG glycan profiling should be specified to have been applied to total serum IgG.
7. Supplementary Figure 2 panel B is not noted (but is described) in the legend.
8. It would be useful to include the number of folds used in cross-validation outside of the supplemental.
9. It feels potentially misleading to refer to the hierarchical clustering of features identified in the supervised setting as "unsupervised" (line 219).
10. Figure 3 panel D would benefit from inclusion of information across replicates and folds for each modeling approach.
11. Figure 3 panels C and D are mis-called out in the text.
12. It would be helpful to identify the "reclassified" Sicca patients in the heatmap or perhaps graphically represent the responses being described in these individuals elsewhere. Otherwise, the readers are really left to take the word of the authors on what is described from line 255 to 261.
13. It is a meaningless detail to indicate that there were 3 steps in a process if none of them are described (line 166).
14. The results section should explicitly note that total serum IgG glycans were profiled - meaning not only antigen-specific, and not only the Fc domain glycan whose importance to FcR binding affinity is described. The relevance of this data to the Ro60-specific data in the second half of Figure 5 is unclear and their combination threatens to mislead reviewers.
15. Figure 5 - last word ("assay") missing?
16. Figure 5 - units in C and D/E do not appear consistent. (One adds 10^-5.)
17. The authors should note whether other FcgR or total/IgG subclass measures were equally or less well correlated with ADCP function to either avoid over- or under-interpretation of the results in Figure 5D/E.
18. Panel 5C - is "BD" healthy? Suggest to make it clear whatever this means or correct the label.
19. New results (supplemental Figure 6) should not be introduced in the discussion section.
20. Citations would benefit from better author diversification. Referencing original methods papers rather than later works by the authors might help address this in some cases.
21. Grammar correction at line 394-395.
22. Tense correction at line 420.
23. Statistical tests in Supplemental Figure 6b. Shouldn't all p value be p<.01, b/c there were 100 replicates and there is a single CV accuracy for the selected feature model. (What is actually being compared? Best, median, mean across the 10-folds?).
24. Figure 2A axis should be clarified as fold-change (as opposed to mathematical difference or some other sort). The authors should also not whether there is a log transformation

Point by Point Response

We want to thanks all referees for their insightful comments, and for helping us improve our manuscript. We have done our best to address all comments, and modified the manuscript accordingly (modification highlighted in yellow in the manuscript). We hope you will see these modifications fit.

Martin KILLIAN
On behalf of all the co-authors

Referee #1 (Comments on Novelty/Model System for Author):

N/A

Referee #1 (Remarks for Author):

Killian et al perform systems serology analysis of patients with Sjögren's Syndrome (SjS), patients with a related syndrome, and healthy individuals. They find that the various systems serology read-outs complement analysis of a broad range of autoantigens, and that they are better able to classify the patient groups. This is an interesting study with clear conclusions and analysis. I have several major concerns that must be addressed before this study would be suitable for publication. However, should these be adequately addressed, this study is a reasonable advancement both in our understanding of SjS and in the application of systems serology to study autoimmune conditions.

Major concerns

If the authors wish to claim that the systems serology data is an important aspect here, they need to present direct evidence indicating so. For instance, how well do their classifiers compare to ones using just the antibody titer data across antigens? Given that they have this data collected, this should be a simple argument to make.

Response: We want to thank Referee #1 for this first comment. We do think that a systems serology-based approach can be interesting for multiple reason in such an heterogeneous and complex disease as Sjogren's syndrome, and we imagined this study as a proof-of-concept study combining machine learning approaches and more "classical" approaches (i.e., one parameter at a time – such as the phagocytosis assay or the glycosylation profiling part), to highlight/illustrate the fact that the identified signatures or possibly disease-driving factors (e.g. Fc/FcR interaction) are not just due to the use of this "unusual" statistical approach.

Hence, we think our data supports the interest of such approach, however, due to the limited size of the cohort (but we are currently working on a bigger scale study), we agree with Referee # about the fact that drawing formal conclusions, at this stage, about the superiority of the systems serology approach would not be reasonable (as we are not bringing sufficiently strong elements to the table to say so). In line with this, our claim was tempered trough the whole manuscript (see also our response to Referee #3's comment #2), especially in the Discussion section (see changes highlighted in yellow in the manuscript) and paid attention not to talk about superiority/inferiority when we were just presenting descriptive data.

That being said, we are reporting the results in terms of sensibility and specificity for our models versus the currently routinely used ELISA-like tests in the text (current lines 166-169 for the SjS vs Healthy controls, and 306-318 for the seronegative SjS vs Healthy controls) and Figure 3D & 7D.

While I appreciate the supervised analysis to identify signatures of the specific patient

<u>groups, some unsupervised analysis of the data would be greatly helpful in understanding the study and cohort. The data collected is rich, and it would be helpful to understand, for instance, whether the Fc properties are similar across antigens, which autoantigens tend to appear together, etc. A simple PCA analysis of the collected data should provide much of this information.</u>

Response: We want to thank Referee #1 for this first comment, and for making us understand that the current version of our manuscript can be improved/better explained for the broader audience of EMBO Molecular Medicine (as some part are of the manuscript are not clear enough even for an expert, familiar with machine learning approaches, like him/her, so it's clearly not clear enough for a broader readership). We have performed PCA analysis in the cohort before running the supervised analysis, but did not include them as they were mostly redundant with the PLSDA. However, Referee #1 is right, including PCA in the manuscript will help, and it was included as Figure EV2. We have also added a text a subsection in the results about this (lines 144-153).
Moreover, we have added multiple subsections in the Methods to make everything clearer, and we hope Referee #1 will see these additions as improvements of the whole manuscript.

<u>I could not find much information on how the patients were diagnosed. Especially given that Sjögren's syndrome is diagnosed in part through an autoantibody test, I would appreciate a detailed description of these procedures. Furthermore, the authors should directly address how their reasoning is not cyclical here; that they are confident they are developing a signature of disease, and not of the diagnostic technique itself.</u>

Response: Patients in our cohort have been diagnosed according to the most recognized classification criteria for SjS, as mentioned in lines 405-410.
Both sets of criteria are available online on multiple websites (including the Sjögren's foundation's and the Rheumatology Scientific Societies') and the original related publications are cited in the manuscript, but here are is a summarized table of the different items (PMID: 29208023)

	2002 AECG criteria	2016 ACR/EULAR criteria	Weight
Items	1. Ocular dryness symptoms<br>2. Oral dryness symptoms<br>3. Ocular signs: Schirmer's test ≤ 5 mm/5 min or van Bijsterveld score ≥ 4<br>4. Focus score ≥ 1 foci/4 mm^2 on minor salivary gland biopsy<br>5. Salivary gland involvement: unstimulated whole salivary flow ≤ 0.1 mL/min<br>6. Positive anti-SSA or SSB antibodies	1. Labial salivary gland with focal lymphocytic sialadenitis and focus score of ≥ 1 foci/4 mm^2<br>2. Anti-SSA/Ro-positive<br>3. Ocular Staining Score ≥ 5 (or van Bijsterveld score ≥ 4) in at least one eye<br>4. Schirmer's test ≤ 5 mm/5 min in at least one eye<br>5. Unstimulated whole saliva flow rate ≤ 0.1 mL/min	3<br>3<br>1<br>1<br>1
Rules for classification	– Absence of exclusion criteria[a]<br>– Presence of any 4 of the 6 items with at least item 4 or 6, or<br>– presence of any 3 of the 4 objective items (3, 4, 5, and 6)	Applies to any individual<br>– who meets the inclusion criteria[b] with at least one symptom of ocular or oral dryness or ESSDAI ≥ 1<br>– does not have any of the conditions listed as exclusion criteria[c]<br>– and has a score of ≥ 4 when the weights from the 5 criteria items are summed	

Plus, we have included the details about the main items (presence/absence of sialadenitis with a focus score ≥1, seropositivity/seronegativity for one of the classical autoantibody anti-Ro/SSA and/or La/SSB) in Table 2 about the demographics.
Interestingly, almost 100% of our cohort had a focus score ≥1 (see Table 2; which is the strongest item in both sets of criteria along with the autoantibodies), but we do understand the remark about the possibility of cyclical reasoning, which is why we have also specifically explored our novel approach in the "seronegative group" (i.e. seronegative for the classical

autoantibodies) and included multiple “non-classical” auto-antigens in the panel (which are not routinely tested, and “don’t count” in the seropositivity/seronegativity assessment).

Moreover, these classification criteria are considered the gold standards for any SjS-related study (including clinical trials and studies evaluating new diagnosis methods such as ours, e.g. PMID: 37147113)

The manuscript seems to be missing several requisite sections, like a data availability statement and competing interests statement, but I figure these will come in revisions.

Response: Thanks for the reminder. The initial manuscript has been sent as a single PDF with text and figures combined, as authorized by EMBO Press. However, we have indeed formatted the revised version of the manuscript as recommended by EMBO Press, and included all the required sections.

Referee #2 (Comments on Novelty/Model System for Author):

The manuscript by Killian et al. applies a Systems Serology platform, previously used in infectious disease research, to identify autoantigen-specific antibody and FcγR signatures associated with Sjögren's Syndrome (SjS), an autoimmune disorder. In addition to detecting known classical autoantibodies, the authors identify multiple novel non-classical autoantibodies associated with SjS, which may help bridge the "seronegative gap" in diagnosis and treatment. These antibodies, along with IgG, FcγR, and C1q engagement, as well as IgG N-linked glycosylation, are linked to ADCP functionality.

Overall, the manuscript is well-written, but several areas require clarification to improve the reader's understanding of the work.

<u>**1. Lines 95-114 should be condensed. The authors devote substantial space to describing general antibody functions and FcγR polymorphisms, though the latter does not appear to play a central role in the study. Some of the discussion could be moved to the introduction to describe relevant previous work done in autoantigen screening (for example, lines 379-401).**</u>

Response: Referee #2 is right. We think FcγR polymorphisms' analysis are interesting as the results are consistent between the high and low affinity polymorphisms for FcγRIIA and FcγRIIIA, plus one of the main findings of the study is the possible novel role of autoantibodies' Fc/FcR engagement, however expanding further on the polymorphism is probably not necessary, and some parts can be condensed.
Hence, we have condensed the lines about FcR polymorphisms (which are now lines 107-114) *"Aberrant expression or allelic variants of FcγRs have been previously associated with autoimmune diseases (Tsang-A-Sjoe et al, 2016), possibly via impaired effector functions (Takai, 2002) or impaired clearance of immune complexes (Koene et al, 1998), but their role in SjS is not yet fully understood (Ben Mkaddem et al, 2019). One study found FcγR-mediated clearance to be abnormal in SjS' patients (Hamburger et al, 1979), though others have not found SjS-linked FcγR-coding gene variants (Haldorsen et al, 2013). Further evaluation of SjS-associated autoantibodies and related Fc effector activity may lead to the identification of novel specific therapeutic strategies or support the repurposing of currently developed autoantibody-targeting drugs."*

The previous part about general antibody functions and their FcR has also been condensed as such (now lines 101-106): *"There are multiple antibody isotypes (i.e., IgG, IgA, IgM, IgD, IgE) and subclasses (IgG1-4, IgA1-2), with specific activating (e.g., FcγRIIa for IgG, which is important for ADCP (Richards et al, 2008)) and inhibitory FcRs (e.g. FcγRIIb for IgG, whose expression is down-regulated in active SLE and SjS (Su et al, 2007; Chen et al, 2013)), that balance pro-inflammatory and anti-inflammatory effector responses to provide protection against pathogens, though when dysregulated can lead to disease (Chalayer et al, 2022)."*

2. Reference 40 (line 162) does not seem to contain a description of the ADCP assay. Could this be an incorrect citation?

Response: Referee #2 is right, previous Ref 40 (by Haycroft ER et al) was incorrectly cited here. "Ackerman ME, Moldt B, Wyatt RT, et al. A robust, high-throughput assay to determine the phagocytic activity of clinical antibody samples. J Immunol Methods. 2011;366:8–19" is the only correct reference (which was previously identified as Ref 41 along with ref 40) for the ADCP assay. The extraneous reference was removed (and the main manuscript's methods have been updated and the detailed methods are still in the Appendix): sorry about this error.

3. Reference 42 (line 167) does not appear to include the referenced "three-step process." Could this be an incorrect citation? Similarly, the same reference is cited in the Supplementary Data under "Feature selection using Elastic Net/PLSR and Elastic Net/PLSDA," which should be verified.

Response: Again, Referee #2 is right, sorry about this error. It is another manuscript by Gunn BM et al which should be cited: "Gunn BM, et al. A role for Fc function in therapeutic monoclonal antibody-mediated protection against Ebola virus. Cell Host Microbe. 2018;24:221–233 e225. doi: 10.1016/j.chom.2018.07.009.". This 2018 manuscript by Gunn BM et al included a 2-step process, and the additional one (which is the 1st in our method), could be summarized like this:
Two thousand smaller data sets of the original cohort were randomly generated, each data set covering at least 80% of the entire cohort, being adjusted to include all categories and fill the minimum of each category. "Randomly sampling without replacement" means that there won't be a duplicate sample selected in the process. Then we use the 2 other steps, first features' preselection with elastic net
This has been corrected, and all the machine-learning-related methods have been moved to the main manuscript for more clarity, plus we have added 2 more recent publications from our team, in Nature Communication, whose studies have used this 3-step process: PMID: 33795692 (which was already included in our reference list) and PMID: 35589689

4. In the "Feature selection using Elastic Net/PLSR and Elastic Net/PLSDA" section of the Supplementary Data, the authors state that they generated 2000 subsets by randomly sampling without replacement. Given the sample size of the data, this seems unlikely Should this instead be "sampling with replacement"?

Response: Thanks for this comment. As responded to comment 3, this is the method and explanations we have used for previous manuscripts from our group (the ones aforementioned PMID: 33795692 ; PMID: 35589689), "without replacement" meaning that each sample unit of the population had only one chance to be selected during the random sampling.
We understand that additional explanations about this are necessary and have included more detailed methods in the corresponding section of the main manuscript (instead of the appendix). See lines 467-502.

5. Given the observed high activation of FcγRIIIa in SjS patients, why was ADCC not performed as well?

Response: We do understand Referee #2's question, and ideally we would have indeed performed ADCC assays, and also wanted to extend these cell-based assays for all the 3 classical autoantibodies, and for some of the non-classical autoantibodies. However, due to the limited amount of serum available, we had to make choices for this proof-of-concept study, and decided to prioritize the ADCP assay with the main autoantibody (i.e., anti-Ro60) and total IgG glycosylation profiling. Moreover, as aforementioned, we are currently working on a bigger scale study, with more patients, and including multiple Fc-related effector functions including ADCC.

6. The Ro/La-only PLSDA model is not described in the text. Was this model generated using Luminex data? How many features does it include?

Response: Referee #2 is right, this model was not properly described in the text, but it was indeed generated with Luminex data (precisions were made about this wherever necessary, i.e., being related to ELISA-like assay data or multiplex assay data), and included "just" anti-Ro60/SSA, anti-Ro52/SSA and anti-La/SSB IgG responses.
Hence, we have removed the "Ro/La-only PLSDA" term across the board, to use a more descriptive term in all the Figures (Fig. 3D, EV3, 7C, 7D, EV6) i.e. "PLSDA using multiplex Ro60, Ro52, La", and updated all the corresponding legends accordingly, plus we have added explanations about this in the main text:

- Lines 164-165: *"(…) for a PLSDA model including only multiplex-based IgG responses to all 3 classical antigens (i.e., Ro60, Ro52 and La)"*
- Lines 173-175: *"(…) for the PLSDA model including only multiplex-based IgG responses to Ro60, Ro52 and La) and for a PLSDA model including only multiplex-based IgG responses to Ro60 and Ro52 (Fig. EV2)."*
- Lines 272-273: *"(…) to a PLSDA model including only multiplex-based IgG responses to all 3 classical antigens (i.e., Ro60, Ro52 and La) (…)"*
- Lines 277-282: *"(…) using the PLSDA model including only multiplex-based anti-Ro60, Ro52 and La IgG responses (Fig. EV5). The sensitivities and specificities for each model are shown in Fig. 6D, with 80.4% sensitivity and 72.2% specificity for the 17-feature signature, 82.7% sensitivity and 66.7% specificity for the 7-feature signature, and 65% sensitivity and 46.7% specificity for the PLSDA model with only Ro60, Ro52 and La."*

7. Lines 214-217 require proper citation for the data being referenced.

Response: Data presented in this section, now in lines 169-172, *"When using multiplex assay data, 77.6% (n=45) patients with SjS were considered seropositive, with only one (2.5%) healthy individual positive for anti-La and another positive for anti-Ro52 (compared to none with ELISA-like assay)."* are referring to multiplex-based data (presented in another way in Figures 2B and EV1), analysed in the same way as ELISA-like assay data i.e. seropositivity meaning at least one positivity among anti-Ro60, anti-Ro52 and/or anti-La. As Luminex results are in MFI, we have used a classical way to calculate the threshold for positivity (i.e. mean of the MFI for each feature in Healthy Donors + 2 standard deviations) and precisions

about this method were added in the *"Customized Sjögren's syndrome bead-based multiplex assay"* subsection of the Methods. As it is not the main message of our manuscript, we have not presented a specific Figure for this, but if Referee #2 thinks it is important, it is possible to do so.

8. Supplementary Figure 2 compares the 7-signature model to PLSDA 3 classical antigens and Ro60+Ro52 models in (A) and Ro/La PLSAD and Multiplex. However, these models are not clearly described in the manuscript and should be explained in more detail.

Response: Referee #2 is right, as mentioned in our response to comment 6, we have included additional data about these models, and modified the Figure (now Figure EV3) accordingly, as the labelling were indeed not clear/not homogenous.

9. Figure legends should include the number of samples per group, as some analyses combine seronegative and seropositive SjS patients, while others separate these groups.

Response: Agreed, these numbers were missing hence, we have added them for each group, in all Figures and EV Figures.

10. Figures 3C and 3D should be swapped to align with the text, as they are currently miscited.

Response: Sorry for this error. It was corrected, but instead of swapping panels 3C and 3D in the Figure, we decided to move the part about Fig. 3C upwards (now lines 160-162), as it fits well there, plus it is now cited before Fig. 3D in the text.

11. Lines 270-279 should include proper citations to support the results described.

Response: We want to thank Referee #2 for this comment. As in our answer to comment 7, we did not think that making a specific figure for the glycosylation profiling analysis including the SjS group as a whole (i.e., without subdividing it into seropositive and seronegative SjS) would add much to the data presented in this subsection (currently lines 210-227) or to the manuscript's main messages, but we still wanted to present some of the results in the text. However, while working on this revised version of the manuscript, as this section was not adding much, we finally decided to just present the data in the 4 groups (seropositive SjS, seronegative SjS, non-SjS sicca syndrome, healthy controls) in the text, plus in Fig. 5A,B and Fig. EV4 (glycosylation data and figures were split from the ADCP ones, according to a following demand). We think it makes sense to focus on these groups (seropositive / seronegative) among SjS, as this sections is about IgG glycosylation, plus it should make the message clearer.

12. In Figure 5A, it is unclear which groups the asterisks are comparing. This should be clarified in the figure legend.

Response: Thanks for the remark. While looking into this, we have also noticed that current Fig. 5A was not as precise as the corresponding text (lines 210-227), with several significant

differences that were not represented. We have tried multiple different options, but we think this one in the best compromise between details and readability:

- Fig 5A is now only descriptive without asterisks or percentage for galactosyltion (but still includes comparisons for fucosylation as we think it's an important feature)
- Previous Supp Fig 4, now Fig EV4 is instead clearly referenced in the text, and includes all the statistical comparisons about galactosylation and sialylation
- As aforementioned, the whole paragraph about total IgG N-glycosylation profiling has been modified for a clearer understanding and the discussion has been updated as well (lines 345-369)
- We have included clarifications/precisions in Fig 5A's and Fig EV4's Legends accordingly

We hope Referee #2 will see these modifications fit**.**

13. In Figure 5C, the label "DB" should be corrected to "Healthy."

Response: Thanks for noticing this as well. It was corrected.

14. Lines 298- show sample numbers lower than the reported group sizes. Was ADCP not measured for all participants? If so, this should be explicitly stated in the Methods section.

Response: Thanks for the remark. ADCP was indeed measured for all participants (as glycosylation profiling and all the other experiments), i.e. 58 SjS, 16 non-SjS sicca syndrome patients and 40 healthy controls. However, as it was specific anti-Ro60 ADCP which was analysed here, the classification is a bit different, and the considered seropositivity is limited to Ro60, whereas in the rest of the manuscript, seropositivity means seropositivity for anti-Ro60 and/or anti-Ro52 and/or anti-La. As Referee #2 can see, the total numbers are the same (33 anti-Ro60 seropositive SjS + 25 anti-Ro60 seronegative SjS = 58 SjS total).
Nonetheless, it needed to be clearer. Hence, we have modified a bit the corresponding text lines 233-235 *"Anti-Ro60 IgG seropositive (according to ELISA-like assay) SjS patients (n = 33 anti-Ro60 seropositive SjS patients out of the 40 SjS patients who are seropositive for anyone of the 3 classical autoantibodies)".*

To limit the risk of confusion, previous Figure 5 has been split: current Figure 5 is showing the glycosylation profiling (+ Fig EV4 with more details) and Figure 6 is for the ADCP assay, as the populations are different (regarding the definition of seropositivity) for both assays.

Figure 6 's groups labelling, as well as its legend, were updated. We have also added a sentence in the corresponding Methods (lines 443-445) about this *"For this assay, anti-Ro60 IgG seropositive (n = 33) and anti-Ro60 IgG seronegative (n = 25) SjS patients were considered, along with non-SjS sicca syndrome patients and healthy controls."*
Plus, the 7 patients who are considered seropositive, but not for anti-Ro60 IgG are mentioned lines 241-242 *"In comparison, SjS patients who were anti-Ro52 IgG only (n=6) or anti-La IgG only (n=1) positive, without anti-Ro60 IgG positivity (…)"*

,

Referee #3 (Comments on Novelty/Model System for Author):

no model system is used in this study

Referee #3 (Remarks for Author):

Killian et al., report application of systems serology tools in the form of multiplex antibody profiling and machine learning to define markers of Sjogren's syndrome. They find that multiplex assay data on a rich number of measures are able to discriminate individuals with different disease status similarly well as simpler feature sets.

Major comments.

1. The modeling approaches are insufficiently well described. For example, the authors describe application of "a range of machine learning models" at line 127, but only reports results from PLSDA with elastic net. Is this the only approach applied? If so, please eliminate referring to this as a range. If not, please provide information on what additional approaches were applied, their performance, and why results only from this approach were presented.

Response: We want to thank Referee #3 for this remark, and for the suggestions to improve the overall clarity of our manuscript. First of all, we have removed the words “a range of” (current line 122) as the main machine learning approach which was used is presented in the manuscript, and is indeed the PLSDA with elastic net approach. In line with this, we have tempered the last sentence of our introduction (i.e., *“Thus, here we applied high-throughput assays to assess antibody binding to 14 autoantigens, FcγR engagement, complement activation and glycosylation, in a cohort of SjS, non-SjS sicca syndrome and healthy individuals and applied a machine learning approach that identified interesting autoantigen-specific antibody and FcγR signatures associated with SjS.”*) as this is a proof-of-concept study, and we agree with Referee #3 about the fact that too strong conclusions cannot be drawn for now.
Moreover, we have included a much more detailed version of the Methods (highlighted in yellow in the manuscript) about these approaches, and hope things will be clearer.
Referee #1 asked us to add an additional unsupervised machine learning approach in the manuscript, so we have added a PCA including the 196 autoantibody-related features in the manuscript, as Figure EV2, and the corresponding text is line 144-153.

2. It is necessary for greater rigor to be applied to the modeling. Applying the same modeling approach on permuted disease class labels in advance of feature selection and class modeling and doing do repeatedly is recommended to aid in interpretation of the quality of performance observed. There is particular concern that 17 feature models learned from 25 seronegative and 40 healthy donors is at risk of being overfit/not performing better on the actual as compared to permuted group labels. Inclusion of

results from randomly selected size-matched feature sets is appreciated, but addresses a different question (how useful was feature selection) than permutation testing (how often would a model perform similarly well by random chance).
Comparison with 100 randomly selected size-matched feature sets seems a difficult to interpret bar as a negative analytical control as these features were all only tested b/c of their possible diagnostic utility. If many features are different between groups, then there is a high probability of a good model based on features selected by chance. Because CV accuracy is greater than 0.7 for many of the random feature sets, would be good to bolster the methodologic approach here through permutation up front and complete model rebuilding, or to temper claims.

Response: Thanks for all these insightful remarks about our approach and its limits. As detailed now in the Methods, we hope our 3-step approach helps in reducing the risk of overfit (and has been used in previous studies, e.g., PMID: 33795692, PMID: 35589689), but we agree with Referee #3 about the fact that the size of our cohort (and the lack of validation cohort) is a significant limit nonetheless, especially for this 17-feature signature which was determined in only a part of the cohort.
As Referee #3 can probably understand, performing a complete model rebuilding would have been more than difficult for this proof-of-concept study (but we are working on a bigger one), so we have indeed tempered our claims/used less strong terms, throughout the whole manuscript.
Here are some examples:

- In the Abstract: *"These findings provide **interesting** insights into the autoantibody responses in SjS, **possibly** paving the way for improved diagnostics, especially in difficult-to-diagnose patients (e.g., seronegative SjS and non-SjS sicca syndrome patients), and novel therapeutic options targeting autoantibody-specific Fc/Fc Receptor-related effector functions"*
- At the end of the introduction *"(…) applied a machine learning approach that identified **interesting** autoantigen-specific antibody and FcγR signatures associated with SjS."*
- Line 270 (we have removed terms suggesting comparison and significant differences, as these are just descriptive) : *"By plotting ROC curves (Fig. 6C), we were able to show the **good** sensitivity and specificity of the 17-feature autoantibody signature in comparison to a PLSDA model including only multiplex-based IgG responses to all 3 classical antigens (i.e., Ro60, Ro52 and La), and the seven-feature signature previously identified by comparing healthy donors versus SjS patients (Fig. 3)."*
- Multiple parts of the Discussion were tempered as well (changes being highlighted in yellow)

Moreover, even if we understand this is not what Referee #3 is asking for, we have kept in the Appendix the supplemental Figure (previously Supplemental Figure 6) including the comparison between the signatures which are highlighted in the manuscript to randomly selected size-matched feature sets, and have added the corresponding charts including their performances, showing calibration errors (average error in the training set) as well as cross-validation error (average error in the test set).

<u>3. Figure 3C is mis-called out in the discussion where figure 3D is meant (and it may be considered unusual to call out figure panels in discussion). This mistake is easily corrected, however, the authors claim that the 7 feature model significantly improved SjS diagnostic performance. This reviewer could find no evidence to support this claim.</u>

Response: Thanks for this comment, and sorry for this error. We have gotten rid of all the citations referring to Figures in the Discussion section, plus this claim has been tempered as part of the modifications related to the previous comment. We think that our approach is interesting for a better understanding of the autoantibodies' role (especially their Fc / FcR interactions), and could also pave the wave for new diagnosis tools including the evaluation of FcGR enagement along with IgG measurement, however, we do agree about the fact these data cannot support a formal claim about improved diagnostic performance.

Here is the new sentence (lines 297-301): "The inclusion of FcγR and C1q engagement, along with IgG measurement, as in the identified SjS-associated seven-feature autoantibody signature, **seemed to have a higher sensitivity for SjS diagnosis compared to the routinely used ELISA-like assay, but it will need to be confirmed in dedicated studies**."

<u>4. Do the authors have an insight into degree of confusion in misclassified samples? Is the model equally or not equally confident about samples it mis as corrected classifies?</u>

Response: We agree with Referee #3 that assessing classification confidence is very important. Despite the limits already mentioned (especially regarding the size of our population, particularly for the 17-feature model) which have lead to our claims' tempering, we think that our 3-step approach involving resampling (2000 iterations), feature selection by stability frequency and validation by cross-validation, helps us say that misclassification here is most probably a reflect of biological overlap between clinical categories in a complex disease such as SjS (i.e., clinically heterogeneous, with just classification and not diagnostic criteria, and the particular difficulties in the forms without anti-Ro60/Ro52/La autoantibodies…), and less likely to be mainly driven by model instability. This is in line with multiple "omics" studies (e.g., PMID: 34112769) who have identified "Healthy-like" clusters among patients classifying as having SjS according to the international criteria.

<u>5. The authors should better explain the relevance of total sersum IgG glycans to antigen specific antibody profiles or to disease states. There is literature on pathogenic alloimmune responses that may be of interest to discuss-especially given fucosylation differences discussed at lines 415-417. Further, the effect of galactose on FcR binding has been rigorously characterized by Dekkers/Vidarsson et al. and ...Barb et al.,. It would seem be better to summarize this highly well-controlled work rather than cop out on the basis of studies of associations in polyclonal sera where many other factors are likely to co-vary.</u>

Response: We want to thank Referee #3's for these suggestions to improve the manuscript, and we have added more precise elements in this subsection of the Discussion from Vidarsson G and Barb AW's high quality work.

Our intention was not to cop out, but to mention the complexity of interpreting these data about glycosylation (especially whereas well-controlled studies are lacking is SjS), as discussed by Dekkers/Viddarson in their 2018 Review (PMID: 29616041) as such:
"Furthermore, in autoimmune diseases, changes in total IgG have been detected in, for example, rheumatoid arthritis (RA) (20, 21), inflammatory bowel disease (22), multiple sclerosis (23), myasthenic gravis (24), ankylosing spondylitis, primary Sjögren's syndrome, psoriatic arthritis (25), and systemic lupus erythematosus (SLE) (26). In all these diseases, a lower degree of total IgG galactosylation is associated with disease progression and flare (20–28). However, the relevance of galactosylation of total IgG, which by definition are not causing the disease, on the disease severity is unknown. In inflammatory autoimmune disorders, such as RA and SLE, IgG autoantibodies presumably play a role in initiating or perpetuating the inflammatory condition (29, 30). In particular in RA, several types of autoantibodies have been identified that target a range of subtle chemical modifications of autologous proteins, including anti-citrullinated protein antibodies (ACPA) and anti-carbamylated protein antibodies—which may be referred to collectively as anti-modified protein response (31). The exact role of the autoantibodies in these diseases has not been fully elucidated as yet, although certain passive transfer mouse models suggest a pathogenic role for ACPAs (32). To our knowledge, the IgG Fc glycosylation patterns have only been determined for disease-associated autoantibodies of a few antigen-specific IgG, including ACPAs and anti-RBC autoantibodies (11, 33–37). In all these diseases, fucosylation is not lowered, even increased for ACPA in RA (11, 33, 34). Several studies suggest similarly low, but variable, levels of galactosylation in the antigen-specific IgG as found in the total IgG (11, 34, 35, 38). However, in two studies, differential changes in glycosylation patterns have been observed between total IgG and specific IgG1 (11, 34), observed in PR3-ACPA and anti-RBC autoantibodies. In both these studies, the galactosylation was variable between the patients, with total IgG galactosylation often diverging from antigen-specific IgG galactosylation"

Of course, antigen-specific antibody glycosylation profiling brings much more precise data about specific antibodies' role and function, however as Referee #3 knows:

- such analysis is sample consuming using the high-throughput techniques we have used (multiple tests have been performed in our group to reach the sensitivity threshold of the Labchip we have used, and it would require starting material of 0.5-1mL of serum, which was not feasible here
- and/or very difficult and time-consuming to perform in cohorts using more sensitive techniques such as mass spec (usually used for smaller groups)

Nevertheless, we think total serum IgG glycosylation profiling still brings interesting data here, but it is important to highlight the limits of this approach.

Here is the updated version of this glycosylation-dedicated section of the Discussion (lines 345-369):

"As FcγR engagement was associated with all the identified autoantibodies and as an important feature in all the selected signature in our cohort, we investigated the potential role of IgG N-linked glycosylation, a well-known factor in modulating IgG Fc binding to their receptors (Chung et al, 2014; Damelang et al, 2024; Dekkers et al, 2018; Yamaguchi & Barb,

2020). Previous SjS-dedicated total antibody glycosylation studies are more than 30-year-old and few, but have observed decreases in sialylation (Youinou et al, 1992), which was not observed in our cohort, and decreases in galactosylation (Parekh et al, 1989). Reduced levels of total IgG galactosylation in both seronegative and seropositive SjS patients in this study is in line with previous publications, along with a range of autoimmune diseases and thought to be reflective of their common proinflammatory state (Seeling et al, 2017; Dekkers et al, 2018; Flevaris & Kontoravdi, 2022). Counterintuitively, it has been experimentally demonstrated that antibody galactosylation, was significantly increasing the affinity for FcγRIIIa (at least in afucosylated antibodies) (Dekkers et al, 2018; Damelang et al, 2024) and C1q activation, hence the in vivo real impact of antibody galactosylaton through FcγR/IgG binding, is impossible to settle without comprehensive data about antigen-specific antibody glycosylation (which could not be performed here due to limited available sample volumes), along with total IgG data which can compete for the same receptors (Dekkers et al, 2018). Increased binding of afucosylated IgG to FcγR (especially FcγRIIIa) has also been well demonstrated, leading to beneficial or detrimental effects (particularly in autoimmune haemolytic and thrombocytopenic diseases) depending on the setting (Oosterhoff et al, 2022), and interestingly fucosylation of total IgG was the lowest in seropositive SjS patients in our cohort. Moreover, seronegative SjS and non-SjS sicca syndrome patients also had lower levels of IgG fucosylation than healthy controls. This is in line with our observation of higher FcγR engagement to autoantibodies in both groups, but further studies including multiple Fc-related effector function assays and antigen-specific autoantibody glycan profiling will be necessary in SjS to address this complex question."

6. The text should make clear that different Abs are being profiled in different parts of Fig

Response: We understand Referee #3's remark, which is in line with Referee #2's comment #14. The text has been updated with precisions about the fact that these individuals are anti-Ro60 seropositive patients (and not anti-Ro60 and/or Ro52 and/or La seropositive patients as in the rest of the manuscript). Plus the Figure legend was updated as well.

5. The legend did a better job of this, but this reviewer nonetheless finds it misleading to lump these panels together.

Response: We do understand Referee #3's remark: the figures have been split to limit confusion. Figure 5 for the glycosylation profiling (+ Fig EV4 with more details) and Figure 6 for the ADCP assay.

7. The manuscript would benefit from better justification of focus on one function.

Response: We do understand Referee #3's remark, which is in line with Referee #2' remark #5. Ideally, we would have indeed performed ADCC assays and other antibody-dependent effector functions' assay + test other autoantigen apart from Ro60, however, due to the limited amount of serum available, we had to make choices for this proof-of-concept study, and decided to prioritize the ADCP assay with the main autoantibody (i.e., anti-Ro60), and also to perform total IgG glycosylation profiling. However, we are currently working on a bigger scale study, with more patients, and the evaluation of multiple Fc-related effector functions.

8. The manuscript insufficiently describes its limitations. These include lack of a validation cohort and lack of clear (statistically significant) improvements over current diagnostic methods, among others.

Response: We agree with Referee #3's comment, which is in line with previous comments which have led to the tempering of some of our claims in different sections of the manuscript and mentioning limits throughout the discussion (and we hope Referee #3 will see these fit). It is indeed important to expand more on our study's limits, especially regarding these elements.

We have modified the second-to-last paragraph of the Discussion section accordingly (changes were highlighted in yellow in the main text – as in the rest of the manuscript): *"An important limitation of this proof-of-concept study is the relatively limited number of individuals in each group, especially for the subgroup analysis, preventing us to formally conclude about a statistically significant improvement of our approach compared to the currently used diagnostic methods. Although the association of FcγR engagement and related effector functions were consistently identified for classical and non-classical autoantibodies in SjS, and via different technical approaches including total IgG glycosylation profiling and an ADCP functional assay, additional studies in bigger independent cohorts, including a validation one, are needed to deepend and confirm our findings, better understand their underlying mechanisms, and determine their possible clinical applications."*

Minor comments.

1. As someone unfamiliar to SjS notation, I found supplementary table 1 to be highly helpful, especially as antigens were introduced and referred to with different subsets of their names over time. It may add sufficient value to merit inclusion as a main table or to provide additional nomenclature information (e.g. SSA, SSB) in figure 1.

Response: As suggested by Referee#3, we have included this Table as Table 1, in the main manuscript.

2. Since Trim21 is an Fc binding protein, how do the authors exclude interactions with this protein though parts of IgG other than CDRs/Fv/Fab regions?

Response: This was indeed a concern when we have designed the original panel (and by the way, this is why we have not included Rheumatoid Factors – i.e., autoantibodies with anti-IgG specificity), however our experimental data ruled this concern out. Ro52/TRIM21-coupled beads were included in all wells (with patients' serum, as well as pooled serum, and control wells with only beads/buffers and then the secondary antibodies), without any significant background or artefactual positivity in patients or controls (all controls were seronegative for anti-Ro52 and lots of patients as well).

Before validating the panels in multiplex, there is a step where we compare singleplex (i.e., with only one antigen-coupled bead in the well, for example Ro52/TRIM21 here) MFI to multiplex (i.e., with all the antigen-coupled beads of the panel in the well) MFI, to identify possible unespected/cross reactivities, and it was not the case for any antigen finally included in the panel, including Ro52/TRIM21.

Moreover, we have used a clinical-grade recombinant (in SF21 insect cells) Ro52/TRIM21 produced by AROTEC (https://www.arodia.com/product/ro52/) for our multiplex panel, which does not lead to constant background signal when testing patients' sample in ELISA or ELISA-like assays, whereas it would have been the case if TRIM21 was interacting with any unspecific IgG in the sample.

3. Unclear what "dedicated" means in the context of its use on line 63.

Response: Agreed, we have removed the term.

4. "findings provides" needs grammatical correction on line 66.

Response: Sorry for the typo. The extra "s" has been removed.

5. "Antibody associated" on line 124 should be hyphenated.

Response: Thanks for noticing. It was corrected as well.

6. At line 157 the application of IgG glycan profiling should be specified to have been applied to total serum IgG.

Response: Agreed. It was modified in the Methods, as suggested, but also in the rest of the manuscript and detailed methods of the Appendix.

7. Supplementary Figure 2 panel B is not noted (but is described) in the legend.

Response: As this panel was not bringing additional valuable data (ROC curves with somewhat similar AUCs between the 7-feature signature and the lower-amount-of-feature approaches), it was removed, and Fig EV3 is now limited to the comparison of the CV Accuracy.

8. It would be useful to include the number of folds used in cross-validation outside of the supplemental.

Response: Agreed. As aforementioned, all the methods regarding the machine learning approach have been included in the main manuscript, for clarity (and now the supplemental methods in the Appendix are just limited to detailed protocols for the ADCP assay / total IgG glycosylation profiling / bead-coupling for the custom multiplex assay.

9. It feels potentially misleading to refer to the hierarchical clustering of features identified in the supervised setting as "unsupervised" (line 219).

Response: We agree with Referee #3, it is most probably misleading. The term was removed.

10. Figure 3 panel D would benefit from inclusion of information across replicates and folds for each modeling approach.

Response: As aforementioned, all the methods about the machine learning approach have been largely improved/detailed in the manuscript *("All models go through 10-fold cross-validation (…)")*, and the Figures' legend were detailed as well (e.g., about experimental replicates – but the informations about the folds were not included in the legends for now – if Referee #3 thinks it is necessary, it can be done though). Plus, we have kept in the Appendix the supplemental Figure (previously Supp Figure 6) including the comparison between the signatures which are highlighted in the manuscript to randomly selected size-matched feature sets, and have added the corresponding charts including their performances, showing calibration errors (average error in the training set) as well as cross-validation error (average error in the test set).

11. Figure 3 panels C and D are mis-called out in the text.

Response: Thanks, it was corrected.

12. It would be helpful to identify the "reclassified" Sicca patients in the heatmap or perhaps graphically represent the responses being described in these individuals elsewhere. Otherwise, the readers are really left to take the word of the authors on what is described from line 255 to 261.

Response: We understand this remark from Referee #3, but we had decided not to add an additional Figure to a manuscript already rich in terms of data, and thought that just describing it would be sufficient, especially as the data from the cohort will be openly accessible, but if the Referee thinks it's important to do, we will be producing a new Figure for this.

13. It is a meaningless detail to indicate that there were 3 steps in a process if none of them are described (line 166).

Response: We could not agree more, sorry about this. As mentioned, all the methods have been greatly detailed, including the part about this 3-step process (lines 467-487).

14. The results section should explicitly note that total serum IgG glycans were profiled - meaning not only antigen-specific, and not only the Fc domain glycan whose importance to FcR binding affinity is described. The relevance of this data to the Ro60-specific data in the second half of Figure 5 is unclear and their combination threatens to mislead reviewers.

Response: As mentioned earlier (see Referee #3 main comment #5 for example), we do agree with the Referee. The terms "Total IgG" were added throughout the manuscript (and the methods + Appendix) to prevent any confusion about this, plus, we have indeed split former Figure 5, into Figure 5 (about glycans) and Figure 6 (about anti-Ro60 ADCP assay).

15. Figure 5 - last word ("assay") missing?

Response: Thanks for noticing. Current Figure 6's legend was globally updated, and corrected regarding this error.

16. Figure 5 - units in C and D/E do not appear consistent.

Response: Sorry about this inconsistency. The method to calculate the "phagocytosis scores" (i.e. %bead-positive cells × median fluorescent intensity) leads to numbers in the hundred thousand, which is not convenient for a Figure, which is why we had added "10^5" (in order to only show 1-2 digt numbers), but nonetheless, it should be consistent within now Figure 6. It was corrected.

17. The authors should note whether other FcgR or total/IgG subclass measures were equally or less well correlated with ADCP function to either avoid over- or under-interpretation of the results in Figure 5D/E.

Response: We want to thank Referee #3 for this suggestion. We have added a sentence in the corresponding subsection of the Results, about this:
"Finally, phagocytosis scores from the whole cohort were significantly correlated with FcγRIIa-H131 (Pearson correlation coefficient r = 0.93; p < 0.0001) and FcγRIIa-R131 (r = 0.92; p < 0.0001) specific engagement of anti-Ro60 autoantibodies (Fig. 6B,C), but also with anti-Ro60 total IgG (r = 0.91), and FcγRIIIa-V158 (r = 0.93), FcγRIIIa-F158 (r = 0.87) and FcγRIIb (r = 0.91) specific engagement (all p < 0.0001). The correlation was not as strong for anti-Ro60 IgG2 (r = 0.42) and IgG4 (r = 0.38; both p < 0.0001), known for their limited ability to engage effector functions.".

18. Panel 5C - is "BD" healthy? Suggest to make it clear whatever this means or correct the label.

Response: Sorry, BD meant Blood Donors, and means Healthy/ Healthy controls. It has been corrected.

19. New results (supplemental Figure 6) should not be introduced in the discussion section.

Response: Agreed, sorry about this. As mentioned in previous responses, this Supplemental Figure 6 has been moved (and expanded) to the Appendix, hence it is no longer introduced in the discussion. We have added this comment at the end of the Results' section:
"Description of the performance (calibration and cross-validation accuracies) of the 3 main identified autoantibody signatures are presented in the Appendix."

20. Citations would benefit from better author diversification. Referencing original methods papers rather than later works by the authors might help address this in some cases.

Response: Thanks for the suggestion. We have added additional references in the Methods, Introduction and Discussion as well, but are open to other suggestions of course.

21. Grammar correction at line 394-395.
Response: Thanks, it has been corrected.

22. Tense correction at line 420.

Response: Thanks, it has been corrected as well.

23. Statistical tests in Supplemental Figure 6b. Shouldn't all p value be p<.01, b/c there were 100 replicates and there is a single CV accuracy for the selected feature model. (What is actually being compared? Best, median, mean across the 10-folds?).

Response: Referee #3 is light, it is misleading to include statistical comparisons in this Supplemental Figure. Hence, it was removed, and the figures are just descriptive.

24. Figure 2A axis should be clarified as fold-change (as opposed to mathematical difference or some other sort). The authors should also not whether there is a log transformation

Response: Agreed, sorry for this imprecision. The labelling of the X axis as been changed to “Fold-change”.

17th Apr 2026

Dear Dr. Killian,

Thank you for the submission of your revised manuscript to EMBO Molecular Medicine. We have heard back from one referee who agreed to re-evaluate your manuscript. This referee also assessed author responses to concerns raised by other referees as they were not available to look at the revised manuscript. As you will see from the report below, referee #1 recognizes the improvements in the revised manuscript but also raises concerns about two methodological concerns not being adequately resolved. After a consultation with my colleagues here, we concluded that raised concerns are justified and should be addressed in an additional and final round of major revision.

Further consideration of a revision that addresses reviewer's concerns in full will entail an additional round of review. Acceptance or rejection of the manuscript will depend on the completeness of your responses included in the next, final version of the manuscript. For this reason, and to save you from any frustrations in the end, I would strongly advise against returning an incomplete revision.

Please also amend following points:
1) Please adhere to the journal's formatting requirements. Please check our Author Guidelines for more information https://www.embopress.org/page/journal/17574684/authorguide#manuscriptpreparation
2) Please correct the order of the manuscript sections to: Abstract / The Paper Explained / Introduction / Results / Discussion / Methods / Data Availability / Acknowledgements / Disclosure and Competing Interests Statement / References / Figure Legends / Tables / Expanded View Figure Legends
3) Please address all comments suggested by our data editors listed below:
- Please note that the exact p values are not provided in the legends of figures 2B, 5B, 6A-C; EV1 A, B; EV4, EV5 A-C.
- Please indicate the statistical test used for data analysis in the legends of figures 2A, EV3 B, EV6 B.
- Please note that information related to n is missing in the legends of figures 5B, EV6 B.
- Please note that the error bars are not defined in the legends of figures EV3 B, EV6 B.
4) Add callouts for Fig 7 and include callouts for individual panels for the EV figures that have panels.
5) Move all Appendix methods and references to the main manuscript file.
6) Please upload Appendix figure as Figure EV7.
7) Please provide: The Paper Explained, Synopsys image, Synopsis text, Reagent Table (as separate .doc file).
8) Please remove "Author contributions" from the manuscript. CRediT has replaced the traditional author contributions section because it offers a systematic machine-readable author contributions format that allows for more effective research assessment. You are encouraged to use the free text boxes beneath each contributing author's name to add specific details on the author's contribution. More information is available in our guide to authors: https://www.embopress.org/page/journal/17574684/authorguide#authorshipguidelines

We would welcome the submission of a revised version within three months for further consideration. Please let us know if you require longer to complete the revision.

I look forward to receiving your revised manuscript.

Yours sincerely,

Zeljko Durdevic

Zeljko Durdevic
Senior Editor
EMBO Molecular Medicine

*****
When preparing your revised manuscript, please refer to our guidelines: https://link.springer.com/journal/44321/submission-guidelines#cms-Revised-submissions. We perform an initial quality control of all revised manuscripts before re-review; failure to

include requested items will delay the evaluation of your revision.

We require:

1) A .docx formatted version of the manuscript text (including legends for main figures, EV figures and tables). Please make sure that the changes are highlighted to be clearly visible.

2) Individual production quality figure files as .eps, .tif, .jpg (one file per figure). For guidance, download the 'Figure Guide PDF': https://media.springernature.com/original/springer-cms/rest/v1/content/27825798/data/v1.

3) A .docx formatted letter INCLUDING the reviewers' reports and your detailed point-by-point responses to their comments. As part of the EMBO Press transparent editorial process, the point-by-point response is part of the Review Process File (RPF), which will be published alongside your paper.

4) A complete author checklist, which you can download from our author guidelines. Please insert information in the checklist that is also reflected in the manuscript. The completed author checklist will also be part of the RPF.

5) Please note that all corresponding authors are required to supply an ORCID ID for their name upon submission of a revised manuscript.

6) It is mandatory to include a 'Data Availability' section after the Materials and Methods. Before submitting your revision, primary datasets produced in this study need to be deposited in an appropriate public database, and the accession numbers and database listed under 'Data Availability'. Please remember to provide a reviewer password if the datasets are not yet public.

In case you have no data that requires deposition in a public database, please state so in this section. Note that the Data Availability Section is restricted to new primary data that are part of this study.

7) For data quantification: please specify the name of the statistical test used to generate error bars and P values, the number (n) of independent experiments (specify technical or biological replicates) underlying each data point and the test used to calculate p-values in each figure legend. The figure legends should contain a basic description of n, P and the test applied. Graphs must include a description of the bars and the error bars (s.d., s.e.m.).

8) At EMBO Press we ask authors to provide source data for the main manuscript figures. You will receive a separate email with instructions for providing source data with your revised manuscript, including how to upload and organize the files.

9) Our journal encourages inclusion of *data citations in the reference list* to directly cite datasets that were re-used and obtained from public databases. Data citations in the article text are distinct from normal bibliographical citations and should directly link to the database records from which the data can be accessed. In the main text, data citations are formatted as follows: "Data ref: Smith et al, 2001" or "Data ref: NCBI Sequence Read Archive PRJNA342805, 2017". In the Reference list, data citations must be labeled with "[DATASET]". A data reference must provide the database name, accession number/identifiers and a resolvable link to the landing page from which the data can be accessed at the end of the reference.

10) We replaced Supplementary Information with Expanded View (EV) Figures and Tables that are collapsible/expandable online. A maximum of 5 EV Figures can be typeset. EV Figures should be cited as 'Figure EV1, Figure EV2" etc... in the text and their respective legends should be included in the main text after the legends of regular figures.

- For the figures that you do NOT wish to display as Expanded View figures, they should be bundled together with their legends in a single PDF file called *Appendix*, which should start with a short Table of Content. Appendix figures should be referred to in the main text as: "Appendix Figure S1, Appendix Figure S2" etc.

- Additional Tables/Datasets should be labeled and referred to as Table EV1, Dataset EV1, etc. Legends have to be provided in a separate tab in case of .xls files. Alternatively, the legend can be supplied as a separate text file (README) and zipped together with the Table/Dataset file.

11) The paper explained: EMBO Molecular Medicine articles are accompanied by a summary of the articles to emphasize the major findings in the paper and their medical implications for the non-specialist reader. Please provide a draft summary of your article highlighting

- the medical issue you are addressing,

- the results obtained and

- their clinical impact.

This may be edited to ensure that readers understand the significance and context of the research. Please refer to any of our published articles for an example.

12) Author contributions: You will be asked to provide CRediT (Contributor Role Taxonomy) terms in the submission system. These replace a narrative author contribution section in the manuscript.

13) A Conflict of Interest statement should be provided in the main text.

14) Every published paper includes a 'Synopsis' to further enhance discoverability. Synopses are displayed on the journal webpage and are freely accessible to all readers. They include a short stand first (maximum of 300 characters, including space) as well as 2-5 one-sentences bullet points that summarizes the paper. Please write the bullet points to summarize the key NEW findings. They should be designed to be complementary to the abstract - i.e. not repeat the same text. We encourage inclusion of key acronyms and quantitative information (maximum of 30 words / bullet point). Please use the passive voice. Please attach these in a separate file or send them by email, we will incorporate them accordingly.

Please also suggest a striking image or visual abstract to illustrate your article as a PNG file 550 px wide x 300-600 px high.

15) Include a Reagents and Tools Table as part of the Methods section, which can be downloaded from our author guidelines.

EMBO Molecular Medicine has a "scooping protection" policy, whereby similar findings that are published by others during review or revision are not a criterion for rejection. Should you decide to submit a revised version, I do ask that you get in touch after three months if you have not completed it, to update us on the status.

***** Reviewer's comments *****

Referee #1 (Remarks for Author):

The authors handled most of referees' comments satisfactorily. However, I have significant concerns that two of the most substantive methodological points were not adequately resolved.

First, regarding referee #3 point 2 (overfitting/permutation testing): the authors defend their resampling strategy by citing its use in prior publications, but this does not address the statistical argument the reviewer was making. While elastic net feature selection is applied to resampled subsets, across the resampled subsets the entire dataset is used to select the variables. Thus, the reported cross-validation accuracies are optimistic by construction. This concern is most acute for the 17-feature seronegative model, which was derived from only 18 seronegative SjS patients and 40 healthy controls. A permutation test, shuffling class labels and rerunning the full pipeline repeatedly, would directly address whether performance exceeds chance, and is not an unreasonable request. The authors acknowledge the small cohort as a limitation but do not engage with the underlying statistical issue.

Second, regarding referee #3 point 3 (model confidence in misclassified samples): the authors appear to have misunderstood the reviewer's question. They interpreted it as asking about the degree of error in misclassified samples and attributed misclassification to biological heterogeneity. However, the reviewer was most likely asking about model calibration-whether the model's predicted probabilities correspond to observed frequencies. This is a distinct and important concept, especially given the class imbalance in the seronegative analysis. It could be addressed straightforwardly with a calibration curve or Brier score and was not addressed at all.

Additionally, a minor issue regarding the PCA plot, I was actually more interested in what the loadings plot would look like. It looks like the authors only included the scores plot. The loadings plot would show the relationships between the serologic variables.

In summary, I would recommend that the manuscript not be accepted in its current form without either a permutation analysis to support the validity of the feature selection pipeline, or substantially more tempered claims regarding the seronegative model specifically. The calibration question should also be addressed directly.

Point by Point Response

We want to thanks all reviewers (especially the last one) for their insightful comments, and for helping us improve our manuscript. We have done our best to address these last 3 comments, and modified the manuscript (last modifications highlighted in yellow in the manuscript, according to the reviewer's last comments and the editorial comments as well) and the figures accordingly. We hope you will see these modifications fit.

Martin KILLIAN
On behalf of all the co-authors

Referee #1 (Remarks for Author):

The authors handled most of referees' comments satisfactorily. However, I have significant concerns that two of the most substantive methodological points were not adequately resolved.

1) First, regarding referee #3 point 2 (overfitting/permutation testing): the authors defend their resampling strategy by citing its use in prior publications, but this does not address the statistical argument the reviewer was making. While elastic net feature selection is applied to resampled subsets, across the resampled subsets the entire dataset is used to select the variables. Thus, the reported cross-validation accuracies are optimistic by construction. This concern is most acute for the 17-feature seronegative model, which was derived from only 18 seronegative SjS patients and 40 healthy controls. **A permutation test, shuffling class labels and rerunning the full pipeline repeatedly, would directly address whether performance exceeds chance, and is not an unreasonable request. The authors acknowledge the small cohort as a limitation but do not engage with the underlying statistical issue.**

Response: We thank the reviewer for this important and insightful comment regarding the risk of overfitting and the need for permutation-based validation.
In response to this concern, we have now performed the requested permutation analysis. Specifically, we repeatedly shuffled the class labels and reran the entire modelling pipeline (n=1000 per model), in order to estimate model performance under the null hypothesis. We have also clarified this point in the Methods and Results sections.
The results of this analysis are now presented in the revised manuscript as additional panels within the relevant Figures (+ their legends), i.e. panel B within Fig EV3 (related to Fig. 3 and already including Cross-Validation Accuracy Data); panel D within Fig 4; panel C within Fig EV6 (related to Fig. 7 and already including Cross-Validation Accuracy Data). We have provided a graphical representation of the selection probabilities for each antibody-related feature under the null hypothesis (i.e., after permutation of class labels). This helps to assess whether the identified features are selected beyond chance levels.
Importantly, the permutation results confirm that the observed model performance and feature selection patterns are unlikely to arise under the null hypothesis regarding the main models, supporting the robustness of our findings despite the overall limited sample size. Regarding the 17-feature seronegative model, which derives from a relatively small number of seronegative SjS patients (n=18) and 40 healthy controls, the permutation tests reveal some degree of overfitting, however the results seem consistent to some degree with the other models (e.g., including FcGR-related data) and with the biological data hinting towards a role for Fc/FcR engagement in SjS. We have further emphasized these elements in the revised Discussion, and also tempered the claims regarding the 17-feature seronegative model at this stage as suggested by the reviewer (+ in line with the permutation test), especially claiming for the need of larger studies dedicated to seronegative SjS in the future.
We thank the reviewer again for this valuable suggestion, which, we think, has strengthened the methodological rigor of our study, and hope she/he will find that the addition of the permutation testing analysis directly addresses the cited concerns.

2) Second, regarding referee #3 point 3 (model confidence in misclassified samples): the authors appear to have misunderstood the reviewer's question. They interpreted it as asking

about the degree of error in misclassified samples and attributed misclassification to biological heterogeneity. However, **the reviewer was most likely asking about model calibration-whether the model's predicted probabilities correspond to observed frequencies**. This is a distinct and important concept, especially given the class imbalance in the seronegative analysis. **It could be addressed straightforwardly with a calibration curve or Brier score and was not addressed at all.**

Response: We thank the reviewer for this important clarification and apologize for our initial misunderstanding of the question.
We recognize that the reviewer was referring to model calibration, i.e., the agreement between predicted probabilities and observed outcome, rather than the magnitude of error in misclassified samples.
To address this point, we have added additional panels within the relevant Figures (and their legends), i.e. panel C within Fig EV3 (related to Fig. 3 and already including Cross-Validation Accuracy Data); panel E & F within Fig 4; panel D within Fig EV6 (related to Fig. 7 and already including Cross-Validation Accuracy Data) showing the relationship between predicted probabilities and observed class labels. Specifically, we plotted the observed status (e.g., seronegative SjS or Healthy control for the data presented in Fig 7 and Fig EV6) of each individual against their predicted probability of being classified as SjS or non-SjS sicca syndrome. This visualization allows assessment of whether higher predicted probabilities correspond to a higher proportion of true cases.
This addition provides an intuitive evaluation of the model's probabilistic outputs and complements the performance metrics already reported. We have also clarified this point in the Methods and Results sections.

3) Additionally, **a minor issue regarding the PCA plot, I was actually more interested in what the loadings plot would look like. It looks like the authors only included the scores plot. The loadings plot would show the relationships between the serologic variables.**

Response: We apologize for not adding the loadings plot in the first place. It has been included in Fig EV2 (as panel B), as the reviewer mentioned he was interested in seeing the relationships between all the variables, however despite our efforts, we have the feeling the current version of the loadings plot is a bit difficult to read, especially as a lot of variables contributing to PC1 were almost overlapping, but we did our best to make things as clear as possible, labelling only the top 10 contributors for each PC (in red for PC1, in yellow for PC2).
Alternatively, we could provide the contribution of the top 10-20 variables for each PC in bars, as in Fig 3B for example.

4) In summary, I would recommend that the manuscript not be accepted in its current form without either a permutation analysis to support the validity of the feature selection pipeline, or substantially more tempered claims regarding the seronegative model specifically. The calibration question should also be addressed directly.

Response: As we have tried our best to follow the reviewer's recommendations for improvement, we hope he/she will find its current form suitable for publication.

22nd May 2026

Dear Dr. Killian,

Please find enclosed the final reports on your manuscript. We are pleased to inform you that your manuscript is accepted for publication and is now being sent to our publisher to be included in the next available issue of EMBO Molecular Medicine.

Your manuscript will be processed for publication by EMBO Press. It will be copy edited and you will receive page proofs prior to publication. Please note that you will be contacted by Springer Nature Author Services to complete licensing and payment information.

You may qualify for financial assistance for your publication charges - either via a Springer Nature fully open access agreement or an EMBO initiative. Check your eligibility: https://link.springer.com/journal/44321/how-to-publish-with-us

Should you be planning a Press Release on your article, please notify the production team via this form in order to coordinate publication and release dates: https://forms.cloud.microsoft/e/eH4A8JVP6g

If you have any questions, please do not hesitate to contact the Editorial Office. Thank you for your contribution to EMBO Molecular Medicine.

Yours sincerely,
Zeljko Durdevic

Zeljko Durdevic
Senior Editor
EMBO Molecular Medicine

Referee #1 (Comments on Novelty/Model System for Author):

While this is a relatively small cohort, the analysis is high quality and provides an advancement in our understanding of Sjogrens.

Referee #1 (Remarks for Author):

The authors have fully addressed all concerns.

>>> Please note that it is EMBO Molecular Medicine policy for the transcript of the editorial process (containing referee reports and your response letter) to be published as an online supplement to each paper. If you do NOT want this, you will need to inform the Editorial Office via email immediately. More information is available here: https://link.springer.com/partners/embo-press/editorial-policies#Peer%20review

EMBO Press Author Checklist

Corresponding Author Name: Martin KILLIAN	
Journal Submitted to: EMBO Molecular Medicine	
Manuscript Number: EMM-2025-21501-V2	

USEFUL LINKS FOR COMPLETING THIS FORM
The EMBO Journal - Author Guidelines
EMBO Reports - Author Guidelines
Molecular Systems Biology - Author Guidelines
EMBO Molecular Medicine - Author Guidelines

Reporting Checklist for Life Science Articles (updated January
This checklist is adapted from Materials Design Analysis Reporting (MDAR) Checklist for Authors. MDAR establishes a minimum set of requirements in transparent reporting in the life sciences (see Statement of Task: 10.31222/osf.io/9sm4x). Please follow the journal's guidelines in preparing your
Please note that a copy of this checklist will be published alongside your article.

Abridged guidelines for figures
1. Data
The data shown in figures should satisfy the following conditions:
- ➞ the data were obtained and processed according to the field's best practice and are presented to reflect the results of the experiments in an accurate and unbiased manner.
- ➞ ideally, figure panels should include only measurements that are directly comparable to each other and obtained with the same assay.
- ➞ plots include clearly labeled error bars for independent experiments and sample sizes. Unless justified, error bars should not be shown for technical
- ➞ if n<5, the individual data points from each experiment should be plotted. Any statistical test employed should be justified.
- ➞ Source Data should be included to report the data underlying figures according to the guidelines set out in the authorship guidelines on Data

2. Captions
Each figure caption should contain the following information, for each panel where they are relevant:
- ➞ a specification of the experimental system investigated (eg cell line, species name).
- ➞ the assay(s) and method(s) used to carry out the reported observations and measurements.
- ➞ an explicit mention of the biological and chemical entity(ies) that are being measured.
- ➞ an explicit mention of the biological and chemical entity(ies) that are altered/varied/perturbed in a controlled manner.
- ➞ the exact sample size (n) for each experimental group/condition, given as a number, not a range;
- ➞ a description of the sample collection allowing the reader to understand whether the samples represent technical or biological replicates (including how many animals, litters, cultures, etc.).
- ➞ a statement of how many times the experiment shown was independently replicated in the laboratory.
- ➞ definitions of statistical methods and measures:
  - common tests, such as t-test (please specify whether paired vs. unpaired), simple χ2 tests, Wilcoxon and Mann-Whitney tests, can be unambiguously identified by name only, but more complex techniques should be described in the methods section;
  - are tests one-sided or two-sided?
  - are there adjustments for multiple comparisons?
  - exact statistical test results, e.g., P values = x but not P values < x;
  - definition of 'center values' as median or average;
  - definition of error bars as s.d. or s.e.m.

Please complete ALL of the questions below.
Select "Not Applicable" only when the requested information is not relevant for your study.

Materials

Newly Created Materials	Information included in the manuscript?	In which section is the information available? (Reagents and Tools Table, Materials and Methods, Figures, Data Availability Section)
New materials and reagents need to be available; do any restrictions apply?	Not Applicable	

Antibodies	Information included in the manuscript?	In which section is the information available? (Reagents and Tools Table, Materials and Methods, Figures, Data Availability Section)
For **antibodies** provide the following information:<br>- Commercial antibodies: RRID (if possible) or supplier name, catalogue number and or/clone number<br>- Non-commercial: RRID or citation	Yes	Reagents and Tools Table

DNA and RNA sequences	Information included in the manuscript?	In which section is the information available? (Reagents and Tools Table, Materials and Methods, Figures, Data Availability Section)
**Short novel DNA or RNA including primers, probes**: provide the sequences.	Not Applicable	

Cell materials	Information included in the manuscript?	In which section is the information available? (Reagents and Tools Table, Materials and Methods, Figures, Data Availability Section)
**Cell lines:** Provide species information, strain. Provide accession number in repository **OR** supplier name, catalog number, clone number, and/**OR** RRID.	Yes	Reagents and Tools Table
**Primary cultures:** Provide species, strain, sex of origin, genetic modification status.	Not Applicable	
Report if the cell lines were recently **authenticated** (e.g., by STR profiling) and tested for mycoplasma contamination.	Not Applicable	

Experimental animals	Information included in the manuscript?	In which section is the information available? (Reagents and Tools Table, Materials and Methods, Figures, Data Availability Section)
**Laboratory animals or Model organisms:** Provide species, strain, sex, age, genetic modification status. Provide accession number in repository **OR** supplier name, catalog number, clone number, **OR** RRID.	Not Applicable	
**Animal observed in or captured from the field:** Provide species, sex, and age where possible.	Not Applicable	
Please detail **housing and husbandry conditions**.	Not Applicable	

Plants and microbes	Information included in the manuscript?	In which section is the information available? (Reagents and Tools Table, Materials and Methods, Figures, Data Availability Section)
**Plants:** provide species and strain, ecotype and cultivar where relevant, unique accession number if available, and source (including location for collected wild specimens).	Not Applicable	
**Microbes:** provide species and strain, unique accession number if available, and source.	Not Applicable	

Human research participants	Information included in the manuscript?	In which section is the information available? (Reagents and Tools Table, Materials and Methods, Figures, Data Availability Section)
If collected and within the bounds of privacy constraints report on age, sex and gender or ethnicity for all study participants.	Yes	Materials and Methods + Table 2

Core facilities	Information included in the manuscript?	In which section is the information available? (Reagents and Tools Table, Materials and Methods, Figures, Data Availability Section)
If your work benefited from core facilities, was their service mentioned in the acknowledgments section?	Not Applicable	

Design

Study protocol	Information included in the manuscript?	In which section is the information available? (Reagents and Tools Table, Materials and Methods, Figures, Data Availability Section)
If study protocol has been **pre-registered, provide DOI in the manuscript**. For clinical trials, provide the trial registration number **OR** cite DOI.	Yes	Materials and Methods
Report the **clinical trial registration number** (at ClinicalTrials.gov or equivalent), where applicable.	Yes	Materials and Methods

Laboratory protocol	Information included in the manuscript?	In which section is the information available? (Reagents and Tools Table, Materials and Methods, Figures, Data Availability Section)
Provide DOI OR other citation details if **external detailed step-by-step protocols** are available.	Not Applicable	

Experimental study design and statistics	Information included in the manuscript?	In which section is the information available? (Reagents and Tools Table, Materials and Methods, Figures, Data Availability Section)
Include a statement about **sample size** estimate even if no statistical methods were used.	Not Applicable	
Were any steps taken to minimize the effects of subjective bias when allocating animals/samples to treatment (e.g. **randomization procedure**)? If yes, have they been described?	Not Applicable	
Include a statement about **blinding** even if no blinding was done.	Not Applicable	
Describe **inclusion/exclusion criteria** if samples or animals were excluded from the analysis. Were the criteria pre-established?<br><br>If sample or data points were omitted from analysis, report if this was due to attrition or intentional exclusion and provide justification.	Yes	Materials and Methods
For every figure, are **statistical tests** justified as appropriate? Do the data meet the assumptions of the tests (e.g., normal distribution)? Describe any methods used to assess it. Is there an estimate of variation within each group of data? Is the variance similar between the groups that are being statistically compared?	Yes	Materials and Methods

Sample definition and in-laboratory replication	Information included in the manuscript?	In which section is the information available? (Reagents and Tools Table, Materials and Methods, Figures, Data Availability Section)
In the figure legends: state number of times the experiment was **replicated** in laboratory.	Yes	Figures
In the figure legends: define whether data describe **technical or biological replicates**.	Yes	Figures

Ethics

Ethics	Information included in the manuscript?	In which section is the information available? (Reagents and Tools Table, Materials and Methods, Figures, Data Availability Section)
Studies involving **human participants**: State details of **authority granting ethics approval** (IRB or equivalent committee(s), provide reference number for approval.	Yes	Materials and Methods
Studies involving **human participants**: Include a statement confirming that **informed consent** was obtained from all subjects and that the experiments conformed to the principles set out in the WMA Declaration of Helsinki and the Department of Health and Human Services Belmont Report.	Yes	Materials and Methods
Studies involving **human participants:** For publication of **patient photos**, include a statement confirming that consent to publish was obtained.	Not Applicable	
Studies involving experimental **animals**: State details of **authority granting ethics approval** (IRB or equivalent committee(s), provide reference number for approval. Include a statement of compliance with ethical regulations.	Not Applicable	
Studies involving **specimen and field samples:** State if relevant **permits** obtained, provide details of authority approving study; if none were required, explain why.	Not Applicable	

Dual Use Research of Concern (DURC)	Information included in the manuscript?	In which section is the information available? (Reagents and Tools Table, Materials and Methods, Figures, Data Availability Section)
Could your study fall under dual use research restrictions? Please check biosecurity documents and list of **select agents and toxins** (CDC): https://www.selectagents.gov/sat/list.htm	Not Applicable	
If you used a select agent, is the security level of the lab appropriate and reported in the manuscript?	Not Applicable	
If a study is subject to dual use research of concern regulations, is the name of the **authority granting approval and reference number** for the regulatory approval provided in the manuscript?	Not Applicable	

Reporting

The MDAR framework recommends adoption of discipline-specific guidelines, established and endorsed through community initiatives. Journals have their own policy about requiring specific guidelines and recommendations to complement MDAR.

Adherence to community standards	Information included in the manuscript?	In which section is the information available? (Reagents and Tools Table, Materials and Methods, Figures, Data Availability Section)
State if relevant guidelines or checklists (e.g., **ICMJE, MIBBI, ARRIVE, PRISMA**) have been followed or provided.	Not Applicable	
For **tumor marker prognostic studies,** we recommend that you follow the **REMARK** reporting guidelines (see link list at top right). See author guidelines, under 'Reporting Guidelines'. Please confirm you have followed these guidelines.	Not Applicable	
For **phase II and III randomized controlled trials**, please refer to the **CONSORT** flow diagram (see link list at top right) and submit the CONSORT checklist (see link list at top right) with your submission. See author guidelines, under 'Reporting Guidelines'. Please confirm you have submitted this list.	Not Applicable	

Data Availability

Data availability	Information included in the manuscript?	In which section is the information available? (Reagents and Tools Table, Materials and Methods, Figures, Data Availability Section)
Have **primary datasets** been deposited according to the journal's guidelines (see 'Data Deposition' section) and the respective accession numbers provided in the Data Availability Section?	Yes	Data Availability Section
Were **human clinical and genomic datasets** deposited in a public access-controlled repository in accordance to ethical obligations to the patients and to the applicable consent agreement?	Not Applicable	
Are **computational models** that are central and integral to a study available without restrictions in a machine-readable form? Were the relevant accession numbers or links provided?	Not Applicable	
If publicly available data were reused, provide the respective **data citations in the reference list**.	Not Applicable	
